# Patient-Reported Outcomes of Omission of Breast Surgery Following Neoadjuvant Systemic Therapy: A Nonrandomized Clinical Trial

**DOI:** 10.1001/jamanetworkopen.2023.33933

**Published:** 2023-09-14

**Authors:** Helen M. Johnson, Heather Lin, Yu Shen, Emilia J. Diego, Savitri Krishnamurthy, Wei T. Yang, Benjamin D. Smith, Vicente Valero, Anthony Lucci, Susie X. Sun, Simona F. Shaitelman, Melissa P. Mitchell, Judy C. Boughey, Richard L. White, Gaiane M. Rauch, Henry M. Kuerer

**Affiliations:** 1 Department of Breast Surgical Oncology, The University of Texas MD Anderson Cancer Center, Houston; 2 Department of Biostatistics, The University of Texas MD Anderson Cancer Center, Houston; 3 Division of Breast Surgery, University of Pittsburgh Medical Center Magee-Womens Hospital, Pittsburgh, Pennsylvania; 4 Department of Pathology, The University of Texas MD Anderson Cancer Center, Houston; 5 Department of Breast Imaging, The University of Texas MD Anderson Cancer Center, Houston; 6 Department of Breast Radiation Oncology, The University of Texas MD Anderson Cancer Center, Houston; 7 Department of Breast Medical Oncology, The University of Texas MD Anderson Cancer Center, Houston; 8 Division of Breast and Melanoma Surgical Oncology, Department of Surgery, Mayo Clinic, Rochester, Minnesota; 9 Division of Surgical Oncology, Department of Surgery, Carolinas Medical Center, Levine Cancer Institute, Atrium Health, Charlotte, North Carolina; 10 Department of Abdominal Imaging, The University of Texas MD Anderson Cancer Center, Houston

## Abstract

**Importance:**

Patients should have an active role in decisions about pursuing or forgoing specific therapies in treatment de-escalation trials.

**Objective:**

To evaluate longitudinal patient-reported outcomes (PROs) encompassing decisional comfort and health-related quality of life (HRQOL) among patients who elected to enroll in a clinical trial evaluating radiotherapy alone, without breast surgery, for invasive breast cancers with exceptional response to neoadjuvant systemic therapy (NST).

**Design, Setting, and Participants:**

Prospective, single-group, phase 2 clinical trial at 7 US medical centers. Women aged 40 years or older with invasive cT1-2 N0-1 M0 triple-negative or human epidermal growth factor receptor 2 (*ERBB2*)–positive breast cancer with no pathologic evidence of residual disease following standard NST enrolled from March 6, 2017, to November 9, 2021. Validated PRO measures were administered at baseline and 6, 12, and 36 months post-radiotherapy. Data were analyzed from January to February 2023.

**Interventions:**

PRO measures included the Decision Regret Scale (DRS), Functional Assessment of Cancer Therapy—Lymphedema (FACT-B+4), and Breast Cancer Treatment Outcomes Scale (BCTOS).

**Main Outcomes and Measures:**

Changes in PRO measure scores and subscores over time.

**Results:**

Among 31 patients, the median (IQR) age was 61 (56-66) years, 26 (84%) were White, and 26 (84%) were non-Hispanic. A total of 15 (48%) had triple-negative disease and 16 (52%) had *ERBB2*-positive disease. Decisional comfort was high at baseline (median [IQR] DRS score 10 [0-25] on a 0-100 scale, with higher scores indicating higher decisional regret) and significantly increased over time (median [IQR] DRS score at 36 months, 0 [0-20]; *P* < .001). HRQOL was relatively high at baseline (median [IQR] FACT-B composite score 121 [111-134] on a 0-148 scale, with higher scores indicating higher HRQOL) and significantly increased over time (median [IQR] FACT-B score at 36 months, 128 [116-137]; *P* = .04). Perceived differences between the affected breast and contralateral breast were minimal at baseline (median [IQR] BCTOS score 1.05 [1.00-1.23] on a 1-4 scale, with higher scores indicating greater differences) and increased significantly over time (median [IQR] BCTOS score at 36 months, 1.36 [1.18-1.64]; *P* < .001). At 36 months postradiotherapy, the cosmetic subscore was 0.45 points higher than baseline (95% CI, 0.16-0.74; *P* = .001), whereas function, pain, and edema subscores were not significantly different than baseline.

**Conclusions and Relevance:**

In this nonrandomized phase 2 clinical trial, analysis of PROs demonstrated an overall positive experience for trial participants, with longitudinal improvements in decisional comfort and overall HRQOL over time and minimal lasting adverse effects of therapy.

**Trial Registration:**

ClinicalTrials.gov Identifier: NCT02945579

## Key Points

**Question:**

How do patients describe their experiences participating in a clinical trial evaluating omission of breast surgery for invasive cancers with exceptional response to neoadjuvant systemic therapy?

**Findings:**

In this nonrandomized phase 2 clinical trial of 31 patients, decisional comfort and health-related qualify of life (HRQOL) were high at baseline and increased significantly during postradiotherapy surveillance (median follow-up 32.4 months). Patients reported a slight increase in cosmetic asymmetry after radiotherapy, but no persistent changes in function, pain, or edema.

**Meaning:**

This analysis of patient-reported outcomes demonstrates an overall positive experience for trial participants, with longitudinal improvements in decisional comfort and HRQOL and minimal lasting adverse effects of therapy.

## Teaser

This nonrandomized clinical trial evaluates patient-reported outcomes in a clinical trial evaluating omission of breast surgery for invasive cancers with exceptional response to neoadjuvant systemic therapy.

## Introduction

Patient-reported outcomes (PROs) are increasingly recognized as essential components of clinical research^1^ and clinical trials.^2^ Collection of self-reported data about patients’ symptoms, adverse effects, and well-being avoids the risk of observer bias^3^ and provides an opportunity to explore whether statistically significant outcomes are clinically meaningful.^4^ International, consensus-based guidelines exist regarding optimal collection,^5^ reporting,^6^ and ethical inclusion^7^ of PROs in clinical trials. For breast cancer specifically, the Translational Breast Cancer Research Consortium offers recommendations for preferred, validated PRO measures (PROMs).^8^

PROs are particularly important in cancer therapy de-escalation trials for several reasons. As these trials can rarely be blinded, substantive patient buy-in and shared decision-making are essential. Patients may have second thoughts about forgoing standard therapies and this uncertainty may influence future treatment decisions. Fears about oncologic safety may also impact well-being and, in turn, clinical outcomes. When oncologic outcomes are noninferior to the standard of care, the impact of de-escalation on health-related quality of life (HRQOL) is particularly meaningful.

This article reports the first planned analysis of longitudinal PROs for a prospective, multicenter clinical trial evaluating the omission of breast surgery for invasive cancers with exceptional response to neoadjuvant systemic therapy (NST). Trial feasibility has been described previously,^9^ and the first planned primary end point analysis demonstrated no ipsilateral breast tumor recurrences (IBTRs) at a median follow-up of 26.4 months.^10^

## Methods

### Trial Design and Participants

This was a prospective, multicenter, single-group, phase 2 clinical trial. Patients were enrolled from March 6, 2017, through November 9, 2021. All participating centers received approval from their institutional review boards. All patients provided written informed consent. This study followed the Transparent Reporting of Evaluations With Nonrandomized Designs (TREND) reporting guideline. The trial protocol appears in Supplement 1.

The trial design and patient selection criteria were detailed previously.^10^ In brief, adult women with early-stage invasive triple-negative or human epidermal growth factor receptor 2 (*ERBB2*)-positive breast cancer treated with standard NST were eligible. After completing NST, participants underwent image-guided, vacuum–assisted core biopsy of the tumor bed to evaluate for breast pathologic complete response (pCR). Individuals with cN1 disease also underwent targeted axillary dissection to evaluate for axillary pCR. Patients without pathologic evidence of residual disease in the breast and axilla continued in the study with omission of breast surgery. Locoregional therapy consisted of standard whole-breast radiotherapy for all patients and regional nodal irradiation for those with cN1 disease. Adjuvant anti-*ERBB2* therapy and endocrine therapy were administered according to biologic subtype. Race and ethnicity were self-reported by the participants and were assessed to obtain the demographic background of the study population. Race options were American Indian or Alaska Native, Asian, Black or African American, Native Hawaiian or Other Pacific Islander, White, and decline to answer. Ethnicity options were Hispanic or Latino, not Hispanic or Latino, and decline to answer.

### End Points

The primary end point was IBTR, which was evaluated at 6-month intervals over 5 years by history and physical examination and diagnostic breast imaging. In cases of abnormal imaging findings or discordance, biopsies were performed to evaluate for recurrence.

Secondary end points included PROs from 3 validated PROMs: (1) the Decision Regret Scale (DRS), a 5-item questionnaire that assesses the extent of regret and comfort associated with a specific decision^11^; (2) the Breast Cancer Treatment Outcomes Scale (BCTOS), a 22-item questionnaire that assesses differences in function, cosmesis, pain, and edema between the affected breast and the contralateral breast^12^; and (3) the Functional Assessment of Cancer Therapy—Lymphedema (FACT-B+4), a 42-item questionnaire that measures HRQOL in breast cancer patients across multiple domains of well-being.^13^ These PROMs were selected by consensus among the multidisciplinary trial group, which included patient advocates, with consideration given to validity, reliability, ease of use, and patient burden. PROMs were administered via secure email, telephone, or US mail. Baseline PROs were collected between the post-NST biopsy and the start of radiotherapy, and follow-up PROs were collected at 6, 12, and 36 months after radiotherapy completion.

### Statistical Analysis

PROMs were scored according to standardized instruments and established precedent.^14,15,16^ The DRS^14^ is scored on a 0 to 100 scale, with higher scores indicating greater regret and lower scores indicating greater decisional comfort. The BCTOS^15^ asks respondents to quantify differences between breasts on a 1 to 4 scale, with 1 indicating no difference; 2, a slight difference; 3, a moderate difference; and 4, a large difference. Results are reported as mean overall scores and means for 4 subscores: function, cosmesis, pain, and edema. The FACT-B+4^16^ is scored such that higher scores indicate higher HRQOL. This questionnaire includes 6 subscales, each scored summatively: physical well-being, social/family well-being, emotional well-being, functional well-being, breast cancer subscale (BCS), and arm subscale (ARM). The FACT-B composite score includes all subscales except ARM and ranges from 0 to 148.

For each PROM, scores were summarized at each time point using descriptive statistics. Linear mixed-effects models were applied to evaluate longitudinal changes in scores, taking intrapatient correlation into account. Univariate models were used to assess associations between PROs and time as well as demographic, tumor, and treatment characteristics. Covariates included PRO time point (baseline, 6 months, 12 months, and 36 months), age at diagnosis (continuous variable), race, ethnicity, cancer subtype (hormone–receptor positive/*ERBB2*-positive, hormone–receptor negative/*ERBB2*-positive, or triple-negative), receipt of axillary surgery, receipt of regional nodal irradiation, receipt of endocrine therapy, and receipt of biopsy during surveillance. Patients with missing or unknown data were excluded from models using the pertinent covariate.

For each PRO score, a multivariable linear mixed-effects model was developed including PRO time point and all covariates with *P* ≤ .10 in the univariate model. After stepwise regression, only PRO time point and covariates with *P* ≤ .05 remained in the final model. All tests were 2-sided. In cases of collinearity, separate final models were created with each collinear covariate. Receipt of biopsies was evaluated over time; the other covariates were treated as baseline variables. Analyses were performed using SAS, version 9.4 (SAS Institute), R, version 3.6.1 (R Project for Statistical Computing), and S-PLUS, version 8.2 (TIBCO Software Inc). Data were analyzed from January to February 2023.

## Results

### Baseline Covariates

**Figure 1.**
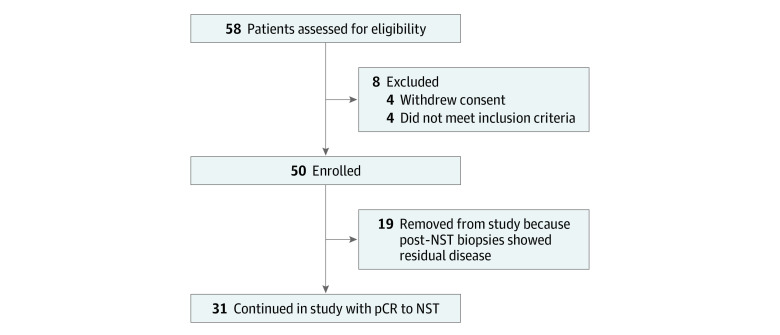
Patient Selection Abbreviations: NST, neoadjuvant systemic therapy; pCR, pathologic complete response.

Fifty-eight patients were assessed for eligibility, 50 were enrolled, and 31 experienced pCR and composed the final cohort (Figure 1). The median (IQR) age was 61 (56-66) years. One patient (3%) was Asian, 4 (13%) were Black, and 26 (84%) were White. Three patients (10%) were Hispanic, 26 (84%) were non-Hispanic, and 2 (6%) did not report ethnicity. Fifteen patients (48%) had triple-negative disease. Sixteen (52%) had *ERBB2*-positive disease, of whom 9 had hormone receptor–negative disease and 7 had hormone receptor–positive disease. Eight patients (26%) had cN1 disease and underwent targeted axillary dissection; 6 also received axillary radiation. Three patients underwent biopsies at 6 months after radiotherapy, 4 underwent biopsies at 12 months, and 3 underwent biopsies at 24 months to rule out IBTR owing to abnormal imaging findings. All biopsies demonstrated benign pathology, most commonly fibrosis, and were deemed concordant with imaging.

### Follow-Up and PROM Completion

At the time of writing in March 2023, the median (IQR) follow-up of the cohort was 32.4 (20.3-40.0) months, and there were no IBTRs. Twenty-eight patients (90%) have been followed up for at least 12 months after radiotherapy. Fifteen (48%) have at least 36 months of follow-up data. All patients completed all 3 PROMs at each administration, except 1 patient completed only the FACT-B+4 at 12 months.

### DRS Scores

**Figure 2.**
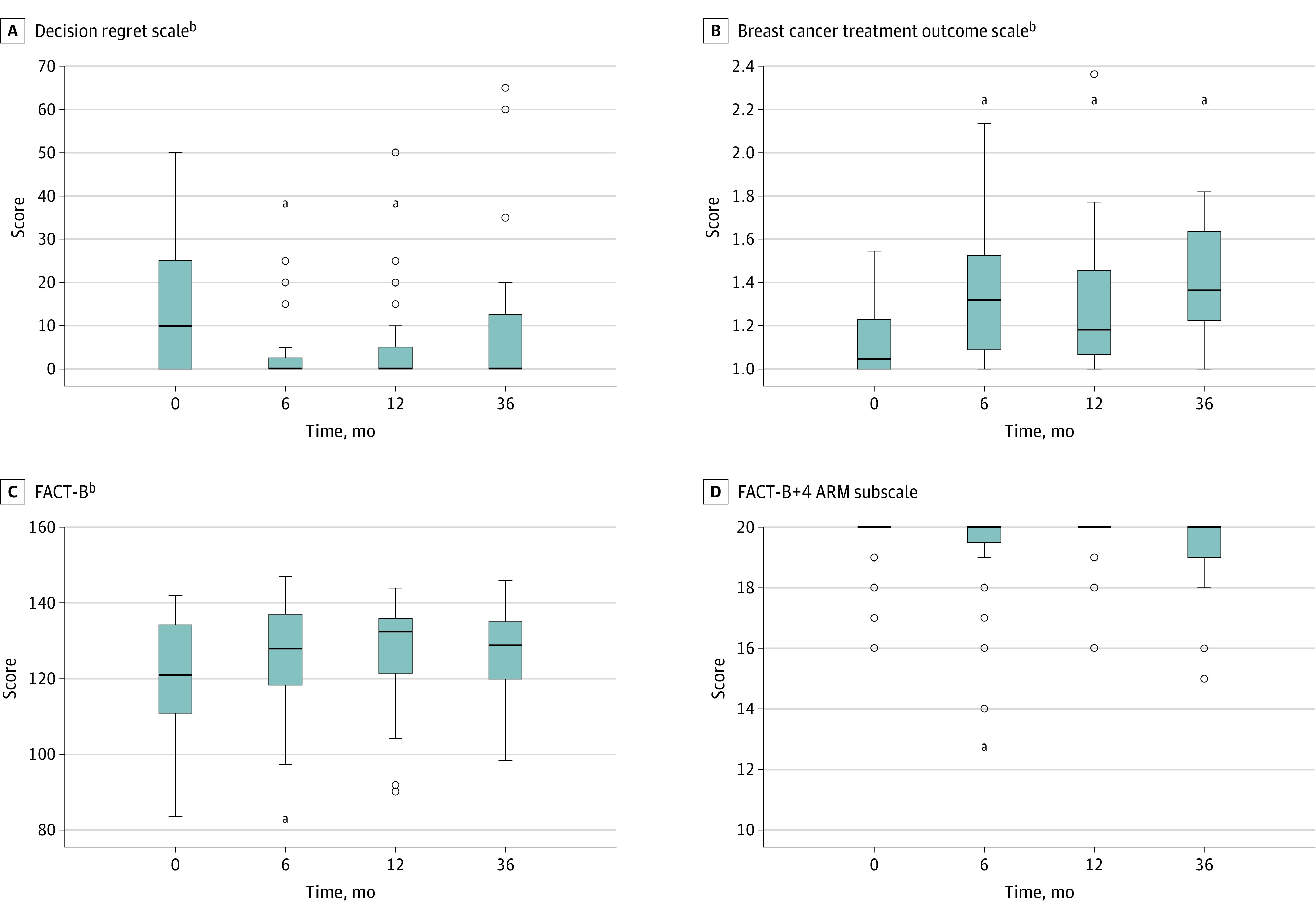
Box-and-Whisker Plots for Patient-Reported Outcome Measure (PROM) Scores Across Time Points ^a^*P* value ≤0.05 for comparisons with baseline. Values that are more than 1.5 times the IQR away from the box are considered to be outliers and shown as circles. The whiskers that extend from the box show the minimum and maximum of the remaining, nonoutlier values. FACT-B indicates Functional Assessment of Cancer Therapy—Lymphedema.

The median (IQR) DRS score was 10 (0-25) at baseline, indicating high decisional comfort; it declined to 0 (0-5) at 6 months, indicating maximal decisional comfort; and it remained stable thereafter (Figure 2A). This overall decrease in DRS scores was significant on univariate analysis (*P* = .004) and was primarily driven by differences over the first 12 months (eTable 1 in Supplement 2).

Besides PRO time point, the only other covariates with *P* ≤ .10 in the univariate model for DRS (eTable 1 in Supplement 2) were age, race, and receipt of axillary surgery. In the final multivariable model (Table 1), age and race remained significantly associated with DRS scores at all time points. Time was independently associated with an overall longitudinal decrease in scores (χ^2^_3 = _18.6; *P* < .001), indicating an increase in decisional comfort over time. This change was primarily driven by differences between baseline and the first 12 months. Older patients had significantly higher decisional comfort than younger patients at baseline and all time points, by approximately −0.3 (95% CI, −0.6 to −0.04) points (*P* = .03) per year of increasing age. White patients had significantly lower decisional comfort than Black or Asian patients, by approximately 7.0 (95% CI, 1.1 to 12.8) points (*P* = .02).

**Table 1.** Final Multivariable Model for Decision Regret Scale Scores^a^

Covariate	Estimate	Standard Error	95% CI	*P* value	Overall *P* value
Time point					
36 mo	−2.3650	6.6868	−15.4710 to 10.7409	.72	<.001
12 mo	−10.1048	2.7144	−15.4250 to −4.7847	<.001	
6 mo	−10.4839	2.7119	−15.7992 to −5.1686	<.001	
Baseline	1 [Reference]	1 [Reference]	1 [Reference]	NA	
Age, per-year increase	−0.3351	0.1506	−0.6303 to −0.0400	.03	NA
Race					
Black or Asian	1 [Reference]	1 [Reference]	1 [Reference]	NA	NA
White	6.9825	6.9825	1.1346 to 12.8304	.02	NA

Abbreviation: NA, not applicable.

^a^ Lower scores (range 0-100) indicate higher decisional comfort.

### BCTOS Scores

The median (IQR) BCTOS score was 1.05 (1.00-1.23) at baseline, indicating minimal between-breast differences; the score fluctuated slightly over time and gradually increased to 1.36 (1.18-1.64) at 36 months (Figure 2B). This overall increase was significant on univariate analysis (χ^2^_3 = _23.8; *P* < .001), with significant differences between baseline and all time points (eTable 2 in Supplement 2).

Besides PRO time point, the only other covariate with *P* ≤ .10 in the univariate models for BCTOS overall score (eTable 2 in Supplement 2) was race. In the final multivariable model (Table 2), both covariates remained significantly associated with BCTOS overall scores at all time points. Time was independently associated with an overall longitudinal increase in scores (χ^2^_3 = _23.7; *P* < .001), indicating increase in between-breast differences over time. White patients reported lesser between-breast differences than Black or Asian patients at baseline and across all time points (*P* = .04), by approximately −0.14 (95% CI, −1.64 to 11.70) points. Univariate models for each BCTOS subscore are available in eTable 3, eTable 4, eTable 5, and eTable 6 in Supplement 2.

**Table 2.** Final Multivariable Models for Breast Cancer Treatment Outcomes Scale Overall Score and Subscores^a^

Covariate	Estimate	Standard Error	95% CI	*P* value	Overall *P* value
Overall score					
Time point					
36 mo	0.1996	0.0658	0.0705 to 0.3286	.002	<.001
12 mo	0.1819	0.0617	0.0609 to 0.3029	.003	
6 mo	0.2317	0.0503	0.1330 to 0.3303	<.001	
Baseline	1 [Reference]	1 [Reference]	1 [Reference]	NA	NA
Race					
Black or Asian	1 [Reference]	1 [Reference]	1 [Reference]	NA	NA
White	−0.1395	0.0676	−0.2719 to 0.0071	.04	NA
Functional subscore					
Time point					
36 mo	0.0691	0.0767	−0.0813 to 0.2195	.37	.04
12 mo	0.1208	0.0459	0.0308 to 0.2108	.01	
6 mo	0.1382	0.0551	0.0303 to 0.2462	.01	
Baseline	1 [Reference]	1 [Reference]	1 [Reference]	NA	NA
Cosmetic subscore					
Time point					
36 mo	0.4683	0.1427	0.1886 to 0.7481	.001	<.001
12 mo	0.2211	0.0833	0.0578 to 0.3845	.008	
6 mo	0.3145	0.0650	0.1871 to 0.4419	<.001	
Baseline	1 [Reference]	1 [Reference]	1 [Reference]	NA	NA
Age: per-year increase	−0.0128	0.0055	−0.0236 to 0.0019	.02	NA
Pain subscore					
Time point					
36 mo	0.1725	0.124	−0.0705 to 0.4155	.16	.01
12 mo	0.3251	0.1424	0.0459 to 0.6042	.02	
6 mo	0.3441	0.1091	0.1302 to 0.5579	.001	
Baseline	1 [Reference]	1 [Reference]	1 [Reference]	NA	NA
Subtype					
Triple negative	0.3709	0.1522	0.0726 to 0.6693	.01	<.001
HR-positive/*ERBB2*-positive	0.5468	0.1554	0.2421 to 0.8515	<.001	
HR-negative/*ERBB2*-positive	1 [Reference]	1 [Reference]	1 [Reference]	NA	NA
Receipt of biopsies during surveillance					
Yes	−0.5077	0.1282	−0.7589 to −0.2565	<.001	NA
No	1 [Reference]	1 [Reference]	1 [Reference]	NA	NA
Edema subscore					
Time point					
36 mo	0.0087	0.0737	−0.1357 to 0.1531	.91	.048
12 mo	0.1108	0.0673	−0.0212 to 0.2428	.09	
6 mo	0.1452	0.0682	0.0115 to 0.2789	.03	
Baseline	1 [Reference]	1 [Reference]	1 [Reference]	NA	NA
Race					
Black or Asian	1 [Reference]	1 [Reference]	1 [Reference]	NA	NA
White	−0.1636	0.0820	−0.3243 to −0.0030	.045	NA

Abbreviations: *ERBB2*, human epidermal growth factor receptor 2; HR, hormone receptor; NA, not applicable.

^a^ Higher scores (range 1-4) indicate greater differences between the affected breast and the contralateral breast.

Results of the final multivariable models for each BCTOS subscore are shown in Table 2. Time was independently associated with overall longitudinal increases in all 4 subscores (*P* ≤ .05), and these increases were primarily driven by differences between baseline and the first 12 months. Only the cosmetic subscore increased from baseline to 36 months (*P* = .001), by approximately 0.5 (95% CI, 0.2 to 0.7) points, indicating slight asymmetry. Age was significantly associated with cosmetic subscores at all time points, with older patients reporting less asymmetry than younger patients (*P* = .02), by approximately −0.01 (95% CI, −0.02 to 0.001) points per year of increasing age. Race was significantly associated with edema subscores, with White patients reporting lesser between-breast differences in edema than Black or Asian patients (*P* = .05), by approximately −0.2 (95% CI, −0.3 to 0.003) points. Tumor subtype was significantly associated with pain subscores at baseline and across all time points (*P* < .001); compared with patients with hormone-receptor-negative/*ERBB2*-positive disease, those with hormone-receptor-positive/*ERBB2*-positive or triple-negative disease reported greater between-breast differences in pain (*P* < .001, *P* = .01, respectively), by approximately 0.5 (95% CI, 0.2 to 0.9) points and 0.4 (95% CI, 0.1 to 0.7) points, respectively. Interestingly, biopsy during surveillance was associated with lesser between-breast differences in pain (*P* < .001), by approximately −0.5 (95% CI, −0.8 to −0.3) points.

### FACT-B+4 Scores

The median (IQR) FACT-B composite score was 121 (111-134) at baseline, indicating high HRQOL; it increased to a maximum of 133 (122-136) at 12 months and declined slightly to 129 (116-137) at 36 months (Figure 2C). This overall increase was significant on univariate analysis (χ^2^_3 = _8.4; *P* = .04), and was primarily driven by differences over the first 6 months (eTable 7 in Supplement 2).

Besides PRO time point, the only other covariate with *P* ≤ .10 in the univariate models for FACT-B composite score (eTable 7 in Supplement 2) was ethnicity. In the final multivariable model (Table 3), both covariates remained significantly associated with FACT-B scores at all time points. Time was independently associated with an overall longitudinal increase in scores (*P* = .04), indicating improvement in HRQOL over time, which was primarily driven by changes in the first 6 months (increase by 6.4 [95% CI, 1.8 to 11.0] points; *P* = .006). Non-Hispanic patients reported lower HRQOL than Hispanic patients at baseline and across all time points (by approximately −10.1 [95% CI, −15.9 to −4.3] points; *P* < .001). Two patients were not included in this model because of nonreporting of ethnicity. Univariate and multivariate models for each of the 5 subscores included in the FACT-B composite score are available in eTable 8, eTable 9, eTable 10, eTable 11, eTable 12, eTable 13, eTable 14, eTable 15, eTable 16, and eTable 17 in Supplement 2.

**Table 3.** Final Multivariable Models for Functional Assessment of Cancer Therapy—Lymphedema Subscores^**a**^

Covariate	Estimate	Standard Error	95% CI	*P* value	Overall *P* value
FACT-B composite score					
Time point					
36 mo	5.1644	3.1992	−1.1060 to 11.4348	.11	.04
12 mo	5.0322	3.4043	−1.6402 to 11.7046	.14	
6 mo	6.3923	2.3365	1.8128 to 10.9719	.01	
Baseline	1 [Reference]	1 [Reference]	1 [Reference]	NA	NA
Ethnicity					
Hispanic	−10.1311	2.9564	−15.9255 to −4.3368	<.001	NA
Non-Hispanic	1 [Reference]	1 [Reference]	1 [Reference]	NA	NA
ARM subscore					
Time point					
36 mo	−0.3461	0.4479	−1.2238 to 0.5317	.44	.10
12 mo	0.0095	0.2356	−0.4522 to 0.4713	.97	
6 mo	−0.6552	0.3245	−1.2912 to −0.0192	.04	
Baseline	1 [Reference]	1 [Reference]	1 [Reference]	NA	NA
Ethnicity					
Hispanic	−0.6850	0.1835	−1.0448 to −0.3253	<.001	NA
Non-Hispanic	1 [Reference]	1 [Reference]	1 [Reference]	NA	NA

Abbreviation: NA, not applicable.

^a^ Higher scores (range 0-148 for FACT-B composite score, range 0-20 for ARM subscore) indicate higher health-related quality of life.

The ARM subscore, the only subscore not included in the FACT-B composite score, was fairly stable over time (Figure 2D). The median (IQR) ARM subscore was 20 (20-20) at baseline and across all time points. Linear mixed-effects models demonstrated that time was not significantly associated with ARM subscores (χ^2^_3 = _6.1; univariate *P* = .11; χ^2^_3 = _6.3; multivariate *P* = .10). Of note, neither axillary surgery nor axillary irradiation was significantly associated with ARM subscores in univariate models (χ^2^_1 = _1.68; *P* = .19; χ^2^_1 = _2.56; and *P* = .11, respectively; eTable 18 in Supplement 2). Ethnicity was the only covariate with *P* ≤ .10 in the bivariate models for ARM subscore (eTable 18 in Supplement 2) and remained independently associated with ARM subscore in the final multivariable model (Table 3). Similar to the results for FACT-B composite score, Non-Hispanic patients reported lower arm-specific HRQOL than Hispanic patients at baseline and across all time points (*P* < .001), by approximately −0.7 (95% CI, −1.04 to −0.33) points.

## Discussion

This is the first report of longitudinal PROs in this emerging field of omission of breast surgery for highly selected patients whose invasive breast cancers have a pCR to NST. The results of this analysis demonstrate an overall positive experience for trial participants, with longitudinal improvements in decisional comfort and overall HRQOL, and minimal lasting adverse effects of therapy. Coupled with the 100% IBTR–free survival rate to date,^10^ these PRO data suggest that omission of breast surgery in highly selected patients may not only be oncologically sound but also offer HRQOL benefits.

Although changes in PRO scores overall were significantly associated with time, most were driven by differences over the first 6 to 12 months. These analyses are limited by the fact that only 15 patients (48%) had 36–month PRO data. It remains to be seen whether short-term trends will persist with longer follow-up and greater numbers of patients. We anticipate complete 5-year data for the entire cohort within the next 3 years.

Our finding that decisional comfort increased over time is unique. A systematic review^17^ found that no prior studies using the DRS for health care decisions reported longitudinal increases in decisional comfort. A study^18^ of women with early-stage breast cancer found that decisional regret significantly increased, albeit slightly, over 4 years of follow-up. Our results may be partly due to the de-escalation nature of our study, as patients who are forgoing a treatment may experience maximal decisional conflict at enrollment. Few prior studies have identified significant associations between decisional comfort and age or race.^17^ One study^19^ of older women with breast cancer showed greater regret about local therapy decisions among Black patients, in contrast to our findings. These disparate results highlight the need for more longitudinal data about the decision-making experiences of breast cancer patients.

Our finding that all BCTOS subscores except pain significantly increased over the first 12 months and then returned to near-baseline values by 36 months is in keeping with findings from a trial^20^ evaluating once-weekly hypofractionated radiotherapy following breast conservation surgery. In their study, overall scores, functional subscores, and pain subscores peaked 6 months after radiotherapy and declined to near-baseline values by 36 months, whereas pain subscores increased from baseline to 6 months and remained persistently elevated over the duration of follow-up.^20^ Raw baseline scores and interval changes in that study were also relatively similar to those in our study. A randomized trial^21^ evaluating conventional vs hypofractionated whole-breast radiotherapy following breast-conserving surgery also showed similar trends and raw values. Multivariable analysis of 5-year longitudinal PRO data from that trial demonstrated no significant durable changes in cosmesis and slight but significant improvements in function and pain over time.^21^ That study also found that older patients reported better cosmetic results over time than younger patients,^21^ similar to our findings.

Our unexpected finding that biopsy during surveillance was associated with lower BCTOS pain subscores is difficult to explain. Biopsy-associated pain may have been less impactful than pain secondary to other interventions, particularly given that chemotherapy and hormone therapy are associated with chronic pain in breast cancer survivors.^22^ Another possible explanation is the phenomenon termed response shift, in which a respondent’s internal frame of reference changes between time points.^23^ It is unclear why receipt of biopsy was not associated with other changes in PROs, and whether results would have been different if any of the biopsies revealed atypical or malignant results.

Prior studies on HRQOL in breast cancer patients have had mixed results, possibly because of differences in PROMs used to measure well-being. For example, a registry study^24^ found that breast cancer survivors had poorer HRQOL than the general population across all domains, with the lowest HRQOL scores generally occurring either 1 year or 3 years after diagnosis and trending upwards at 5 years after diagnosis. In the aforementioned trial^21^ of conventional vs hypofractionated radiotherapy, a composite score for HRQOL comprising multiple FACT-B+4 subscores significantly improved across the 5 years of follow-up.

Few prior studies have examined ARM subscores from the FACT-B+4 questionnaire, and to our knowledge this is the first study of longitudinal changes in arm-specific PROs. Our finding that neither axillary surgery nor axillary radiation was significantly associated with ARM subscores is reassuring. Although breast pCR and axillary pCR are highly correlated,^25^ pathologic evaluation of the lymph nodes was essential for ruling out residual disease before offering local therapy de-escalation. Targeted axillary dissection has low rates of lymphedema and postoperative complications,^26^ and scars are generally small and easily hidden, affording opportunities for minimal impact on HRQOL.

The observed associations between PRO scores and race or ethnicity must be interpreted with caution given the low number of patients in some categories and missing ethnicity data for 2 patients precluding their inclusion in some models. Our observation that Black patients experienced more edema-related asymmetry than White patients is congruent with a prior report^27^ of greater lymphedema symptoms among Black women than women of other races. Other studies showed lower HRQOL across multiple domains of well-being for Black breast cancer survivors compared with White survivors.^28,29^ The sparse PRO data for underrepresented racial and ethnic groups highlights the importance of recruiting diverse participants to clinical trials.^30^

As this is the first trial we know of to evaluate omission of breast surgery for invasive cancer based on image-guided biopsy after NST, there are few data with which to compare our results. PROs for cryoablation, a minimally invasive procedure, might be similar to PROs for a nonoperative approach. Indeed, in a nonrandomized study^31^ of 34 patients with small, low–risk invasive breast cancer undergoing either standard surgery or cryoablation, cryoablation was associated with higher physical, sexual, and cosmetic well-being subscores.

### Limitations

Limitations of our study include the lack of a comparison group consistent with a single-group multicenter phase 2 clinical trial design. This design minimized the risks of dropout and disappointment bias associated with randomized de-escalation trials in which participants may have strong treatment preferences.^3^ Another limitation is the inability to discern whether statistically significant longitudinal changes in PROs were meaningful given the lack of consensus about definitions for minimally important differences.^17,32,33^ Additionally, given the relatively short follow-up, the results of this first planned analysis must be interpreted with this limitation.

## Conclusions

In this nonrandomized phase 2 clinical trial, analysis of PROs demonstrated an overall positive experience for trial participants, with longitudinal improvements in decisional comfort and overall HRQOL over time and minimal lasting adverse effects of therapy. Longer follow-up in this trial and forthcoming results of similar trials will provide additional insight into the potential HRQOL benefits of omitting breast surgery for highly selected patients whose breast cancers have a pCR to NST.

## Notes

Supplement 1.
Trial Protocol

Supplement 2. **eTable 1. **Associations Between Decision Regret Scale (DRS) Score and Patient Factors, Bivariate Analyses (Time Indicator Is Included in All Models) **eTable 2. **Associations Between Breast Cancer Treatment Outcomes Scale (BCTOS) Score and Patient Factors, Bivariate Analyses (Time Indicator Is Included in All Models) **eTable 3. **Associations Between Breast Cancer Treatment Outcomes Scale (BCTOS) Cosmetic Subscore and Patient Factors, Bivariate Analyses (Time Indicator Is Included in All Models) **eTable 4. **Associations Between Breast Cancer Treatment Outcomes Scale (BCTOS) Pain Subscore and Patient Factors, Bivariate Analyses (Time Indicator Is Included in All Models) **eTable 5. **Associations Between Breast Cancer Treatment Outcomes Scale (BCTOS) Edema Subscore and Patient Factors, Bivariate Analyses (Time Indicator Is Included in All Models) **eTable 6. **Associations Between Breast Cancer Treatment Outcomes Scale (BCTOS) Functional Subscore and Patient Factors, Bivariate Analyses (Time Indicator Is Included in All Models) **eTable 7. **Associations Between Functional Assessment of Cancer Therapy—Lymphedema (FACT-B+4) FACT-B Composite Score and Patient Factors, Bivariate Analyses (Time Indicator Is Included in All Models) **eTable 8. **Associations Between Functional Assessment of Cancer Therapy—Lymphedema (FACT-B+4) Physical Well-Being Subscore and Patient Factors, Bivariate Analyses (Time Indicator Is Included in All Models) **eTable 9. **Associations Between Functional Assessment of Cancer Therapy—Lymphedema (FACT-B+4) Physical Well-Being Subscore and Patient Factors, Multivariable Analyses **eTable 10. **Associations Between Functional Assessment of Cancer Therapy—Lymphedema (FACT-B+4) Social/Family Well-Being Subscore and Patient Factors, Bivariate Analyses (Time Indicator Is Included in All Models) **eTable 11. **Associations Between Functional Assessment of Cancer Therapy—Lymphedema (FACT-B+4) Social/Family Well-Being Subscore and Patient Factors, Multivariable Analysis **eTable 12. **Associations Between Functional Assessment of Cancer Therapy—Lymphedema (FACT-B+4) Emotional Well-Being Subscore and Patient Factors, Bivariate Analyses (Time Indicator Is Included in All Models) **eTable 13. **Associations Between Functional Assessment of Cancer Therapy—Lymphedema (FACT-B+4) Emotional Well-Being Subscore and Patient Factors, Multivariable Analysis **eTable 14. **Associations Between Functional Assessment of Cancer Therapy—Lymphedema (FACT-B+4) Functional Well-Being Subscore and Patient Factors, Bivariate Analyses (Time Indicator Is Included in All Models) **eTable 15. **Associations Between Functional Assessment of Cancer Therapy—Lymphedema (FACT-B+4) Functional Well-Being Subscore and Patient Factors, Multivariable Analysis **eTable 16. **Associations Between Functional Assessment of Cancer Therapy—Lymphedema (FACT-B+4) Breast Cancer Subscore and Patient Factors, Bivariate Analyses (Time Indicator Is Included in All Models) **eTable 17. **Associations Between Functional Assessment of Cancer Therapy—Plus Lymphedema (FACT-B+4) Breast Cancer Subscore and Patient Factors, Multivariable Analysis **eTable 18. **Associations Between Functional Assessment of Cancer Therapy—Plus Lymphedema (FACT-B+4) ARM Subscore and Patient Factors, Bivariate Analyses (Time Indicator Is Included in All Models)

Supplement 3.
Nonauthor Collaborators

Supplement 4.
Data Sharing Statement
