# Supplementary material for: Patient-Reported Outcomes of Omission of Breast Surgery Following Neoadjuvant Systemic Therapy: A Nonrandomized Clinical Trial
Source: JAMA Netw Open. 2023 Sep 14;6(9):e2333933. doi: 10.1001/jamanetworkopen.2023.33933 (PMC10502524; doi:10.1001/jamanetworkopen.2023.33933)
Supplement: Supplement 1. — Trial Protocol [file jamanetwopen-e2333933-s001.pdf]

2016-0046

Multicenter trial for eliminating breast cancer surgery or radiotherapy in exceptional responders to neoadjuvant systemic therapy

Study chair: Henry M. Kuerer, MD, PhD, FACS
Department of Breast Surgical Oncology

Study co-chairs: Savitri Krishnamurthy, MD
Department of Pathology
Benjamin D. Smith, MD
Department of Radiation Oncology
Vicente Valero, MD
Department of Breast Medical Oncology
Wei T. Yang, MD
Department of Diagnostic Radiology
Gaiane M. Rauch, MD
Department of Diagnostic Radiology
Simona Shaitelman, MD
Department of Radiation Oncology
Melissa Mitchell, MD
Department of Radiation Oncology

Multicenter External Participating Investigators:

Mayo Clinic:
Judy Boughey, MD

University of Pittsburgh Medical Center:
Emilia Diego, MD

Levine Cancer Institute, Carolinas Medical Center
Richard White, MD

Protocol Body

1.0 Background Summary

With improvements in chemotherapy regimens, targeted therapies, and our fundamental understanding of the relationship of tumor subtype and pathologic complete response(pCR), there has been dramatic improvement in pCR rates in the past decade, especially among triple negative and HER2-positive breast cancers. Rates of pCR in these groups of patients are in the 30-50% range and thus question the paradigm for the necessity of breast and nodal surgery in all cases. Particularly when they will be receiving adjuvant local therapy with radiotherapy. Current practice for patients who respond well to neoadjuvant systemic therapy (NST) is often to proceed with the same breast and axillary procedures as would have been offered women who had not received NST, regardless of the apparent clinical response. Given these high response rates in defined subgroups among exceptional responders it is appropriate to question whether surgery is now a redundant procedure in their overall management.

The historical rationale for breast conserving therapy has been to use surgery to remove gross disease followed by radiation to remove clinically occult disease. This strategy is highly effective. With advances in NST, chemotherapy now effectively eliminates gross disease in a large proportion of patients. It appears that we can now identify patients who will be found to have a pCR based on post- chemo, pre-surgery large volume, vacuum- assisted core biopsy. We hypothesize that these patients, identified as biopsy negative after chemotherapy, do not need surgery because chemotherapy has already removed the gross disease, effectively obviating the need for surgery. We therefore propose to omit surgery because systemic therapy has removed the gross disease, and treat these patients with standard radiotherapy alone following biopsy-negative response to chemotherapy. Further, definitive radiation without surgical resection with or without systemic therapy has been proven effective for several other malignant disease sites including some stages of esophageal, anal, laryngeal, prostate, and lung carcinoma. The main prior impediments for potential elimination of surgery have been the fact that current standard and functional breast imaging methods are incapable of accurate prediction of residual disease. However, recent data from Germany and MD Anderson Cancer Center have found that integrating percutaneous biopsy of the breast primary following NST may circumvent this issue. Historical attempts at omission of surgery following NST in an earlier era were unsuccessful and associated with higher locoregional recurrences. Now, combining high quality breast imaging together with image-guided percutaneous sampling of the tumor bed appears to be an accurate manner to select patients who would be found to have pCR if surgery were subsequently performed. This protocol describes how this hypothesis will be tested in a local regional control study.

Another approach to de-escalate local therapy in the era of improved systemic treatment is omission of radiation, rather than elimination of surgery. Radiation is associated with a risk of lung damage, heart damage and secondary cancers. There is also financial toxicity from traveling daily to treatments and extended time off work. Lumpectomy alone may be a preferred choice for some patients undergoing breast cancer treatment. Particularly in HER-2 positive patients, the role of radiation has been questioned, as early stage patients have excellent local control rates in the current era. This protocol will offer lumpectomy alone without radiation as an alternative option in HER-2 positive patients who are exceptional responders.

1.1 Historical perspective and results of early studies testing omission of surgery

1.1A Data based on response to NST

Prior studies performed in earlier eras before high quality breast imaging, improved systemic therapy, and our understanding of molecular subtypes and response to systemic therapy seem potentially doomed as clinical response is notoriously inaccurate in determining pathologic residual disease. Further, radiation with systemic therapy as a definitive local treatment has been proven effective for several other malignant disease sites including some stages of esophageal, anal, laryngeal, prostate, and lung carcinoma [1-7]. Early studies evaluated radiotherapy as the definitive local modality for treating the breast in patients who have a clinical complete response to NST, which resulted in unacceptable high locoregional failure rates. Essentially, physical examination and imaging were not able to identify a group of patients without or with only minimal residual disease.

In the study of Touboul et al. [8] performed at Hôpital Tenon in Paris, 97 patients with locally advanced nonmetastatic and noninflammatory breast cancer were treated between 1982 and June 1990. Three different locoregional approaches were proposed, depending on the tumor response. In 37 patients (38%) with residual tumor noted on physical exam to be larger than 3 cm in diameter, located behind the nipple, or with multicentricity, mastectomy and axillary dissection were performed. Sixty other patients (62%) either had breast conserving surgery or no surgery: 33 patients (34%) achieved complete remission and had no surgery with an additional radiation boost, 27 patients (28%) who had a residual mass less than or equal to 3 cm in diameter were treated by wide excision and axillary dissection followed by a boost to the excision site. The 5-year actuarial locoregional relapse rate was 16% after radiotherapy alone, 16% following wide excision and radiotherapy, and 5.4% following mastectomy ($p = 0.04$). Five- and 10-year overall survival rates were not influenced by the local treatment.

The randomized trial reported by Mauriac et al. [9] from Institut Bergonié (Bordeaux, France) included 272 women with operable breast adenocarcinoma larger than 3 cm. Of these, 124 were treated by initial chemotherapy. Forty-four patients had a complete clinical remission and were treated with radiotherapy only. Forty patients with residual tumor (less than 20 mm) were treated with lumpectomy, axillary node dissection and radiotherapy. Forty-nine patients with residual tumors (greater than 20 mm) had mastectomies. After a median follow- up of 34 months there was a slight increase in locoregional recurrence in the group of complete responders to primary chemotherapy that did not have surgery (four of 44) compared with those partial responders who did (two of 40).

In the study of Scholl et al. [10] from Institut Curie in Paris (France) 196 patients received primary chemotherapy for tumors considered too large for BCS. Among these, 45 patients achieved complete clinical response and underwent RT alone. Their 5-year local recurrence free survival was significantly lower than that in the group of 23 patients with partial response (PR) who underwent surgery when there was residual clinical disease after the first 54Gy delivered locoregionally (70% vs. 84%). In neither of these studies nor in other series was there any difference between the two groups with regard to overall survival. Similar findings have been reported in the prospective study of Perloff et al. [11] from the Cancer and Leukemia Group B and in several retrospective series exploring the potential of omitting surgery [10, 12-15].

Even in a more recent large retrospective series of Daveau et al. [12] from Institut Curie in Paris

(France) of patients treated between 1985 and 1999 with NST for early stage breast cancer no significant differences in overall, disease-free and metastasis-free survival rates could be demonstrated between the surgical group (n= 65) and the exclusive radiotherapy group (n=100). However, a nonsignificant trend towards higher locoregional recurrence rates (LRR) was observed in the no- surgery group (31% vs. 17% at 10 years; p = 0.06). They therefore concluded that surgery is a key component of locoregional treatment for breast cancers that achieve clinical complete response to neoadjuvant chemotherapy.

In contrast, the prospective study of DeLena et al. [13] showed no significant difference between the treatment groups with regard to local failure (29.6% vs. 31.1%). Surgical removal of the breast and axillary lymph nodes failed to improve the relapse free and the overall survival rates over the classical radiotherapeutic approach. Notwithstanding, local failure rates in the 30% range are not consistent with modern breast cancer management and would be considered unacceptable.

In a more recent retrospective study of core biopsy alone of the breast primary followed by radiotherapy, Clouth et al. [16] reported a local recurrence of 13% (2 of 16) at a mean of 33.5 months in patients with a pCR. The main issue with this study was that the multiple negative core needle biopsies were performed by random non-image guided biopsy of the quadrant of the breast where the tumor was and under the nipple at the time of surgery for the axillary lymph nodes. This type of procedure would not be consistent with current essential breast imaging guided biopsy procedures and could easily miss the region of known previous carcinoma.

In a meta-analysis of Mauri et al. [17] 9 randomized trials were included with a total 3946 breast cancer patients treated between 1983 and 1999, that compared neoadjuvant systemic treatment to adjuvant systemic treatment. Neoadjuvant therapy was equivalent to adjuvant therapy in terms of survival and overall disease progression. However, NST was associated with a statistically significant increased relative risk for locoregional recurrences (RR 1.22; 95% CI = 1.04 to 1.43, P = .015) when radiotherapy without surgery was adopted.

When interpreting the results of these studies we have to bear in mind several things:
1) locoregional treatment consisted mostly of exclusive radiotherapy before 1991 and breast surgery thereafter; 2) the therapeutic strategies reflect a previous era/ different time periods; and 3) the essential need to utilize state-of- the-art breast imaging with biopsy. Further, with the current improvements in neoadjuvant therapy (e.g. addition of taxanes and targeted treatments), response rates have increased over recent decades, especially in the ER-/HER2+ subgroups and this might be the most appropriate group to select for the potential for eliminating surgery.

1.1B Data Based on Response to Neoadjuvant Endocrine Therapy

For estrogen receptor positive breast cancers, anti-estrogen therapy has been given with the goal of decreasing the size of relatively large breast cancers in the preoperative setting. The primary purpose of these studies was to both increase the change for breast conserving therapy in tumors that were relatively large and historically would have been treated with mastectomy, as well as to study the in vivo effect of anti-estrogen therapy. As such, these studies examined estrogen receptor positive tumors that were larger and of more advanced stage than what is being proposed here, but still provide a foundation of information regarding likely response to

anti-estrogen therapy.

The ACOSOG Z1031 trial of 377 postmenopausal women with stage II-III estrogen receptor positive breast cancer reported on three distinct neoadjuvant anti-endocrine therapy regimens (exemestane, letrozole, or anastrazole) for 16-18 weeks preoperatively [18]. In these relatively large breast tumors, the 74% had a complete or partial response on physical examination with anti-endocrine therapy alone and an additional 19% had stable disease. Only 6.5% of evaluable patients had progressive disease.

Akashi-Tanaka et al. examined the clinical response rate of tumors >3cm in size treated with neoadjuvant tamoxifen or anastrazole for four months preoperatively [19]. Clinical response was defined as complete disappearance or ≥ 50% decrease in tumor area from baseline. The overall clinical response rate to this therapy was 39.5%. However, when examining patients based on the 21-gene expression profile assay recurrence score (RS) (Oncotype) typically used to risk stratify estrogen receptor positive tumors, they authors found a clinical response rate of 64% among patients with a RS <18, compared to a clinical response rate of 31% with either an intermediate (18-30) or high RS (≥31).

A more recent publication by Ueno et al examined clinical response rate by recurrence score among estrogen receptor positive tumors >3cm treated with neoadjuvant exemestane for 24 weeks [20]. Clinical response was defined in this study as complete disappearance or ≥ 30% decrease in tumor area from baseline. These investigators found a significantly higher clinical response rate in those with a low RS (<18) compared to those with a high RS (≥31), 59.4% vs. 20.0%. For those with an intermediate RS (18-30) the clinical response rate was 58.8%.

These studies both point to the fact that neoadjuvant estrogen therapy leads to reduction in the size of large ER positive tumors and that this response varies based on Oncotype Recurrence Score.

1.1C Data Based on Focal and Ablative Dose Radiation to Breast Cancers

With technological advances in radiation therapy, it has become feasible to use image guidance to more focally deliver radiation treatment. As a result of this increased focus in the radiation beam, which minimizes irradiation to normal adjacent tissues, it has become possible to dose escalate radiation to the level of ablating a tumor. These techniques were originally developed been used in the setting of inoperable lung cancer [21] and metastases to the liver [22-23] and spine [24-25]. With the emergence of data supporting high tumor control and an acceptable toxicity profile, stereotactic ablative radiotherapy has increasingly been used as a definitive treatment, particularly in lung cancer, leading to the ability to avoid surgery even in operable patients [26-28].

In the setting of breast cancer, efforts to deliver focal radiation have focused largely on favorable, estrogen receptor positive breast cancers. These approaches have included the use of hypofractionated radiation and stereotactic ablative dose radiation. These studies of delivering radiation to an intact breast tumor have largely been done prior to surgery, with a goal of determining the degree of pathologic complete response that could be achieve. Investigators from the University of Maryland have delivered a dose of 38.5Gy in 10 twice daily fractions to the partial breast, after which surgery was performed at least 3 weeks following the completion of radiation. Using an alpha/beta ratio of 3.0 for breast tumor control, the biologic effective dose

(BED) of this regimen was 87.9. These researchers found a pathologic complete response rate of 15% [29]. The PAPBI Trial Investigators performed a multi-institutional phase II study comparing preoperative to postoperative partial breast irradiation delivering a dose of 4Gy x 10 fractions or 6Gy x 5 fractions, followed by lumpectomy after 6 weeks. A subset of 48 of 70 patients treated on this study found that 12.5% observed a near complete (6.25%) or complete (6.25%) response [30]. The radiation used in this study had a BED of 90-93Gy. Most striking, investigators at the University Medical Center in Utrecht have delivered a dose regimen of 21Gy in 1 fraction to the partial breast followed by surgery 6 months later. In contrast to the aforementioned radiation regimens, this dose regimen has a much higher BED of 168.0. In this setting, investigators found a pathologic complete response rate of 33% [31]. Recently submitted data from this group has shown that in an expanded cohort of an additional 21 patients treated with the same radiation regimen but who waited 8 months until surgery, the pCR rate was 48%. Patients in this study did not receive anti-estrogen therapy. The striking difference in pathologic complete response rates between these studies is likely due to two factors: 1) the significantly higher BED delivered by the investigators at the University Medical Center in Utrecht and 2) the greater time delay allowed between radiation and surgery, enabling the full effect of radiation in what is typically a slowly dividing tumor.

Notably, these studies allowed tumors up to 3cm in size, and it is known that responsiveness to ablative dose radiation is most pronounced the smaller the size of the irradiated tumor. Additionally, fractionation of dose is thought to also increase both tumor control and reduce toxicities to normal adjacent organs.

For those patients who are able to achieve a pCR, it is appropriate to question whether surgery would be a redundant procedure in their overall management. Having an adequate technique to identify pCR without undergoing complete surgery is a critical for this to occur.

1.2 Imaging for primary tumor response monitoring and prediction of pathologic complete response

There has been increasing interest in determining whether negative imaging after systemic therapy might identify a subset of patients that could be treated safely with radiation alone without surgery. Safe omission of surgery in patients who receive neoadjuvant therapy and achieve a radiological complete response (rCR) depends on the ability to accurately estimate pCR preoperatively. However, it is important to keep in mind that even the best imaging of the breast and nodal regions are currently lacking sufficient sensitivity and specificity to select patients who indeed have no residual disease. Further, overall complete radiologic response may occur in a very small minority of patients in the 20% range and despite this, up to 50% of patients will indeed have no residual disease among patients with triple negative and HER2Neu positive breast cancers [32]. Also, there is variation in the definition and assessment methods of a clinical or imaging complete response (cCR) among studies despite the Response Evaluation Criteria In Solid Tumors (RECIST) criteria, since they fail to address breast imaging- specific aspects in detail [33]. The pCR prediction is of high interest because a valid prediction of residual tumor absence could strongly influence the need for and the extent of surgery. With increasing pCR (ypT0) rates the demand to diagnose pCR with noninvasive methods will increase.

Most previous studies evaluated the absolute measurement of residual disease of several imaging modalities and not the attainment of pathologic CR [34-43]. However, only the latter is a

surrogate for meaningful clinical benefit such as disease free or overall survival. To further specify and quantify pCR prediction via breast imaging (MRI, mammography, ultrasound) several studies have been performed to assess false negative rate (FNR) and negative predictive value (NPV), which are expected to be the most important and interpretable measures for addressing the question of diagnostic prediction. The FNR quantifies the number of patients with residual tumor not detected. The NPV quantifies the number of patients correctly identified as pathologic complete responders. The accuracy varied greatly, both among the different imaging modalities and for the breast and axilla, which demonstrates that at present there is no optimal imaging modality for PCR.

Conventional Imaging
Mammography and breast ultrasonography are the most commonly used imaging modalities for estimating primary tumor size at the initial diagnosis. However, their accuracy for assessing residual tumor and tumor changes following NST is variable given the development of tumor fibrosis, fragmentation, remaining intraductal carcinoma after the disappearance of the invasive component, and/or change in tumor density [44, 45].

In a retrospective review of 192 patients no difference was found in the ability of mammography or breast ultrasound to predict pathologic complete response [46]. However, when both mammography and breast ultrasound demonstrated no residual disease, the likelihood of a pathologic complete response was 80%, which is similar to results recently reported by Peintinger et al. from the University of Texas MD Anderson Cancer Center [45]. The use of both imaging modalities improved the accuracy of predicting a pathological complete response to neoadjuvant chemotherapy in a greater percentage of cases than use of either modality alone.

In a prospective study of 43 patients at the University of Michigan it was demonstrated that the accuracy of physical examination, mammography, ultrasound, and MRI in determining pCR was 75, 89, 82, and 89%, respectively. The accuracy of these imaging modalities was not observed to be significantly different. In over half of the cases where pCR was achieved, the imaging modality still suggested residual tumor [47]. It was therefore concluded that biopsy after NST remains absolutely necessary to determine pCR, as the accuracy of current imaging modalities is insufficient to make this determination [47]. A more recent retrospective study confirmed this [48]. The NPV of all methods was less than 50%. The accuracy and NPV were compromised even further in younger patients. MRI was superior with regard to accuracy and positive predictive value (PPV), but the NPV of MRI remained poor at 65%. In their data synthesis, which included 6 other studies to evaluate the accuracy of imaging in determining pCR, all tested modalities had a PPV of 85% or greater and an overall accuracy ranging from 74% for mammography to 84% for MRI. The NPV for mammography, ultrasound and MRI was 41%, 44% and 65% respectively [47].

Schaefen et al. [49] from the University Breast Unit in Heidelberg (Germany) retrospectively evaluated breast imaging procedures for predicting pCR after NST in 150 invasive breast cancers. The NPV of mammography, ultrasound and MRI were 52%, 51% and 60%, respectively. The false negative rates of mammography, ultrasound and MRI were 13%, 24% and 4%, respectively. MRI was the most valid imaging technique for predicting pCR. However, neither MRI nor mammogram or ultrasound was able to predict a pCR (ypT0) with sufficient accuracy to replace the pathologic diagnosis of a surgical excision specimen.

Newer imaging approaches using contrast-enhanced ultrasonography (CEUS), and shear wave

elastography (SWE- which measures pretreatment tumor stiffness) have shown some promise in predicting pathologic response the neoadjuvant setting, although the data are limited [50, 51].

Microcalcifications and predicting residual breast disease
Although residual DCIS after NST does not portray worse prognosis compared to complete eradication of all disease, viable disease must be surgically excised to prevent recurrence [52, 53]. The presence of residual DCIS does not affect long- term outcome, it has clinical implications regarding the surgical management of the patient and may at times lead to the need for more extensive resections, including the need for mastectomy despite excellent response of the invasive component to neoadjuvant chemotherapy. The intraductal component of a tumor can be eradicated with NST as well. In modern day series there is no residual invasive cancer and DCIS in 3.4-19% of cases [52, 54, 55]. Approximately 35% of DCIS cases have overexpression of HER2. Several reports exist on the effect of NST on microcalcifications [56-66]. Microcalcifications can increase, decrease or remain stable after the administration of NST. However, the majority of these reports demonstrate no changes in the malignant appearing microcalcifications after NST. Similar changes in the evolution of microcalcifications on mammography were observed after primary radiation therapy.[67]

Investigators at MD Anderson evaluated patients with pure HER2 amplified DCIS receiving one single dose of trastuzumab followed by surgery with evaluation of histologic changes, measurements of proliferation and apoptosis and the immunologic effects in patients receiving the drug compared with patients not receiving the drug. Compared with patients not receiving drug, there was significant development of antibody-dependent cellular cytotoxicity, which was mediated by natural killer cells among patients receiving trastuzumab. However, no histologic changes were evident even though the patients mounted an immune response. This may have been a result of too few treatments (just one single dose of trastuzumab) or of performing the surgery early after treatment [68].

Functional Imaging
Conventional techniques for assessing response (mammography, ultrasound) rely on macroscopic changes in tumor size, which are often delayed and do not always correlate with the pathologic response. As NST causes an angiogenic response, which occurs prior to a reduction in tumor size, changes in tumor vasculature and metabolism could serve as more sensitive measures of a response to systemic therapy, thus enabling tailoring of treatment. As these changes cannot be assessed by conventional methods, functional imaging techniques such as dynamic contrast- enhanced (DCE)-MRI, diffusion-weighted imaging, and nuclear imaging have been used with more success [69]. Contrast-enhanced MRI depicts changes in morphology and perfusion, whereas PET/CT visualizes changes in glucose metabolism.

Breast MRI

MRI primarily detects breast cancer by contrast enhancement related to tumor angiogenesis. MRI has an increased sensitivity for detecting residual disease in the breast compared with either mammography or ultrasound, making it a potentially useful tool in this setting. In a meta-analysis of Marinovic et al.[70] 44 studies between 1990 and 2008 were included with in total 2050 patients undergoing MRI and/or comparator tests to evaluate residual disease after NST. Studies generally showed high sensitivity (correct detection of residual tumor), with evidence of heterogeneity in the estimates of specificity (correct identification of pCR). The capability of MRI for differentiating the presence of residual malignancy from pCR had an overall AUC of 0.88 and

that overall accuracy differed according to definition of pCR and study timeframe. An increase in accuracy was seen when residual DCIS was excluded in the pCR definition AUC, which is consistent with previous studies that reported lower MRI sensitivity in detecting DCIS relative to invasive cancer [71]. Similarly, MRI has been observed to have limitations in detecting scattered, microscopic tumor foci after NST [72, 73]. Analysis of 7 studies that compared MRI to mammography showed that the latter had lower accuracy than MRI (AUC 0.89 vs 0.95, p = .02). There was only weak evidence that MRI had higher accuracy than clinical examination (p = .10). Accuracy favored MRI in four studies; in the remaining seven studies, MRI was observed to have higher sensitivity but lower specificity than clinical examination. No difference in MRI and ultrasound accuracy was found (p = .15) [70].

As demonstrated in a meta-analysis by Houssami et al.[74] different breast cancer subtypes show different pCR rates. To date, six published studies have shown a correlation between MRI findings and response to NST based on different breast cancer subtypes. Chen et al.[75] evaluated the predictive accuracy of MRI after NST based on overexpression of HER2 and demonstrated that HER2-negative tumors have a high false- negative rate (6 out of 33 patients, 18 %). In the majority of patients (4/6, 66 %), nonmass-like enhancement was found on MRI. HER2-negative and HR-positive cancers and lesions showing nonmass-like enhancement are more likely to show residual disease as small foci or scattered cells after NST, leading to underestimation of the extent of residual disease on MRI, and the diagnostic results of MRI should be used with caution in surgical planning. Hayes et al.[76] investigated the differences in MRI features between estrogen receptor (ER)-negative and ER-positive breast cancers, but they were inconclusive about how accurate MRI is in detecting response of such tumors to NST. Loo et al.[77] found that the changes in MRI during NST correlate well with pathology outcome in triple-negative and HER2-enriched tumors, but not in ER-positive/HER2- negative tumors. McGuire et al.[78] demonstrated that MRI was more accurate in determining the actual pathologic size of residual disease in patients with HER2- enriched and triple negative tumor subtypes (MRI/pathologic size difference < 0.1 cm) than it was for patients with luminal subtypes (MRI/pathologic size difference 1.1 cm) (p= 0.015). Overall, the positive predictive value (PPV) was 91.3% (MRI predicting residual disease), but the negative predictive value (NPV) was only 52.0% (MRI predicting pCR) (p <0.0001). The negative predictive value for pCR was much greater in triple negative and HER2-enriched tumors than in luminal tumors (73.6 vs. 27.3%). A radiologic complete response was seen in 38% (77/203), while a pathologic complete response was achieved in only 25% (51/203). Data from the multi-center De Los Santos et al.[79] study showed similar results. In their study 182 of 746 patients (24%) achieved an rCR, and 179 of 746 patients (24%) achieved a pCR. The negative predictive value was highest for patients who had HR- negative/HER2-positive and triple negative breast cancers. They confirmed that, among patients who achieved an rCR, positive HR status and low tumor grade were most commonly associated with residual disease at surgery, suggesting that an rCR on preoperative MRI in these patient populations should be interpreted with caution. This information underscores the potential importance of repeating vacuum assisted core needle biopsies post-chemotherapy, even with apparent residual disease in HER2 positive and TN cases. Schaefgen et al.[35] demonstrated that the prediction of pCR with MRI depended on the breast cancer subtype with the highest performance of MRI in triple negative cancer with NVPs of 93 and 100 % and FNRs of 5 and 0 % for the near-cCR and cCR categories, respectively. False positive rates also varied widely from 6 % (for TN cancers in MRI) to 60 % (for HER2-/HR+ cancers in US) [49].

In a systematic review of Lobbes et al.[80] based on 34 studies evaluating the diagnostic

accuracy of MRI to detect pCR after NST, the negative predictive value for predicting pCR with MRI ranged from 71–100 %. MRI proved more accurate in determining residual disease than physical examination, mammography and ultrasound. However, both overestimation and underestimation of residual disease were observed. Diagnostic accuracy of MRI after neoadjuvant chemotherapy could be influenced by treatment regimen and breast cancer subtype.

In conclusion, MRI is an effective tool for predicting response to NST. The accuracy of MRI in estimating post chemotherapy tumor size varies with tumor subtype. It is highest in ER- /HER2+ and triple negative tumors and lowest in luminal tumors.

Knowledge of how tumor subtype affects MRI accuracy can guide recommendations for surgery following NST. MRI accuracy for residual DCIS has to be evaluated. This measurement is important for deciding a patient's candidacy for breast conservation and is usually made by mammogram detection.

PET

There is growing evidence that PET imaging early during chemotherapy may help tailor treatment by identifying early non-responders given its pooled sensitivity of 84 % (range 78–88 %) and pooled specificity of 66 % (range 62–70 %) [81-83]. The main limitations of PET and PET/CT for evaluating primary breast lesions are their inability to detect lesions measuring <1 cm reliably and to differentiate benign from malignant pathology, resulting in a relatively high false positive rate [84]. Also, the radioisotope uptake is based on tumor subtype, with more avid isotope uptake in invasive ductal cancers, ER- negative tumors, and triple negative tumors [85].

In a study of 47 women with large, newly diagnosed breast cancers, a marked reduction in fluorodeoxyglucose uptake after one cycle of treatment [50–60 % decrease in the maximum standard uptake value (SUVmax)] was correlated with having a pCR [86]. Pengel et al.[87] evaluated the additional value of FDG PET/CT and dynamic contrast- enhanced MRI in predicting pathological response to NST of breast cancer and the dependency on breast cancer subtype in 93 patients. PET/CT and MRI showed comparable value for monitoring response during NST. The combined use of PET/CT and MRI had complementary potential. There was a higher rate of (near-) pCR in HER2- positive and triple-negative tumors than in ER-positive tumors: 76.0%, 67.9% and 12.5%, respectively (p<0.001).

They also found that in ER-positive/HER2-negative tumors, (near) pCR is never achieved at relative reductions in SUVmax on PET/CT of less than 40%, independent of the reduction in largest tumor diameter on MRI. Conversely, in triple-negative tumors, (near) pCR is related to relative reductions in SUVmax on PET/CT as well as relative reductions in largest diameter of initial and late enhancement on MRI. In multivariate analysis a large relative reduction in SUVmax on PET/CT, a large relative reduction in the largest tumor diameter at late enhancement on MRI, and breast cancer subtype were independent markers for a (near-) pCR. A combination of these features led to an increased AUC, suggesting an improved ability to differentiate between responders and non-responders to NST by applying both modalities in combination with knowledge of the breast cancer subtype.

Koolen et al.[88] of the same group demonstrated that response monitoring with PET/CT during NST in breast cancer seems feasible but is dependent on the breast cancer subtype. PET/CT

may predict response in ER-positive/HER2-negative and triple negative tumors, but seems less accurate in HER2-positive tumors. The same author demonstrated in another study that in triple-negative tumors a PET/CT scan after 6 weeks (three cycles) appears to be optimally predictive of pCR. In HER2-positive tumors neither a PET/CT scan after 3 weeks nor after 8 weeks seems to be useful. The changes in SUVmax of both the tumor and axillary nodes combined correlates best with pCR [89].

In studies comparing PET with MRI in women undergoing NST, PET and PET/CT tend to have higher sensitivity (100 vs. 89 %) but lower specificity (63 vs. 96 %), which can lead to delays in definitive treatment and unnecessary biopsies [90, 91].

1.3 Utilizing image guided percutaneous biopsy to select patients for entry into the study

There has been great international interest in testing the safety of omitting surgery in some breast cancer patients with exceptional response to NST. Recent published results utilizing this approach from Germany, have demonstrated a false negative rate of 0% and a negative predictive value of the 100% among 16 patients where mammographic image guided vacuum assisted biopsy was performed after NST[92]. An update from the Heidelberg University Hospital group presented in abstract form also found that among 38 cases utilizing image guided vacuum assisted biopsy the false negative rate was 4.8% with a negative predictive value of 94.4%[93]. Similarly, preliminary data from MD Anderson protocol 2014–0139 has shown that utilizing percutaneous image guided biopsy among 28 patients receiving NST demonstrated only one false negative result in which there was a region of tissue with 0.6 cm that had a viable cellularity of 2%. This amount of disease can be controlled with standard whole breast radiotherapy with boost. Based on these data, the protocol working group believes that it will be safe to treat these patients with radiation alone in the absence of surgery as it has been shown that patients with an excellent response to NST also tend to demonstrate very low locoregional recurrence rates as well[94].

1.4 Predicting nodal response and axillary imaging

Ultrasound imaging is a fast, noninvasive and inexpensive modality to evaluate the axilla. The presence of cortical thickening is the main feature used to determine malignant axillary lymph nodes (ALN) [95]. In a meta-analysis of Alvarez et al.[96] the accuracy of preoperative US to detect ALN metastases was evaluated. The gold standard in the 9 included studies was either axillary lymph node dissection (ALND) or sentinel node biopsy (SNB). In studies that used lymph node size as the criterion for positive ALN, the overall sensitivity and specificity were 68% (range, 66%-73%) and 88% (range, 44%-97%), respectively. When lymph node morphology was used as the criterion for positivity, the overall sensitivity and specificity were 82% (range, 68%-92%) and 96% (range, 80-97%), respectively [96]. Ultrasound guided biopsy of the sonographically suspicious nodes somewhat increases the specificity, which reaches 100%. They concluded that axillary ultrasound is moderately sensitive and fairly specific in the diagnosis of axillary metastatic involvement. Negative ultrasound results do not exclude axillary lymph node metastases.

Several studies have examined the utility of US alone in patients who have undergone NST. Kuerer et al. studied 147 women with locally advanced breast cancer who underwent physical examination and US at diagnosis and after NST. After NST, the sensitivity, specificity, PPV, and

NPV of US were 62%, 70%, 83%, and 44%, respectively [97]. Similarly, Klauber- Demore et al. [98] observed that the use of US after NST had a PPV of 83% with an NPV of only 52%. Finally, Vlastos et al.[99] studied US in patients with less advanced breast cancer (stage II) who received NST and observed that the PPV and NPV of US were both low at 67% and 49%, respectively, and the overall accuracy of US was 57% for correctly classifying ALN status. They found that 48% of patients who had negative ultrasound and physical examination after neoadjuvant chemotherapy had pathologically positive nodes, and when both physical examination and ultrasound were positive, 20% of patients had pathologically negative nodes [27]. Kuerer et al. [35] found that among 55 patients in whom physical examination and ultrasound of the axilla were negative after neoadjuvant chemotherapy, 53% were found to have axillary lymph node metastases on pathologic examination. The sensitivity of ultrasound was 62%, and the specificity was 80%. These studies demonstrated that US imaging alone is not sufficient to accurately stage the axilla in the NST setting. The recent cooperative group study of Boughey et al. confirms this by showing that ultrasound after chemotherapy of the nodes has only a 50% chance of determining residual disease [100].

Fine needle aspiration (FNA) improves the accuracy of US. The specificity of US-guided FNA is nearly 100%, and its sensitivity ranges from 44% to 95%. The NPV ranges from 66 to 96%.[101] Hieken et al.[102] retrospectively analyzed the performance of axillary US, MRI, and FDG-PET in detecting ypN-positive disease after NST. In their study, 128 of 272 patients had ypN-positive disease. Post-NST imaging included axillary US (146 patients), MRI (139 patients), and FDG-PET (38 patients). Axillary US was the most sensitive test for detecting ypN-positive disease after NST with a sensitivity of 70% compared with
FDG-PET (63%) and MRI (61%). The accuracy was best for FDG-PET (72%) followed by axillary US (65%) and MRI (60%). The negative predictive value of axillary US, MRI and FDG-PET were 56.8%, 42.5% and 61.1%, respectively. The accuracy of current imaging modalities in predicting the axillary nodal response to treatment was 60 to 72 %.
Therefore, imaging does not necessarily preclude surgical axillary staging for patients after completion of NST [102].

In a prior report of 47 cN+ patients (as determined by SNB or image guided axillary node biopsy) on the performance of post-NST breast MRI the sensitivity and specificity of MRI to identify residual pathologic axillary lymph node disease following NST were 85.7% and 89%, respectively, while the positive and negative predictive values were 92% and 80.9%, respectively [103]. In the node positive patients MRI was able to predict with moderate sensitivity and specificity whether residual nodal disease was present. The sensitivity of MRI is not yet sufficient to replace the gold standard of pathologic or cytologic examination for diagnosis of axillary node metastases. The accuracy of MRI is not adequate to obviate either the need for staging with sentinel node biopsy or the need for completion axillary dissection in women determined to be node positive prior to neoadjuvant therapy.

Previous studies have demonstrated a high positive predictive value of PET/CT for the detection of axillary metastases (96–98%), indicating that prechemotherapy SLNB can be omitted in cases of an FDG-avid node [104, 105]. In patients with biopsy-proven ALN metastasis, FDG-PET can help delineate the extent of locoregional disease by identifying the number and location of lymph node metastases. The reported accuracy of FDG-PET for detecting axillary lymph node metastases varies from 74-95% with a sensitivity of 61- 95% and a negative predictive values from 79-95% [101]. Koolen et al.[104] reported the results of FDG-PET in detecting ALN metastases among 311 women who underwent US- guided FNA or SNB before

the initiation of NST. FDG-PET had a sensitivity, specificity, PPV, NPV, and accuracy of 82%, 92%, 98%, 53%, and 84%, respectively. The same institution also reported on the results of early PET-CT assessment of axillary response with a sensitivity, specificity, and accuracy of 16–76, 46–97, and 57–71%, respectively, for the detection of persistent node-positive disease [106]. FDG-PET may be a useful tool for monitoring disease response in the axilla, particularly among women who have triple- negative disease [106, 107].Rousseau et al. [108] confirmed that axillary response monitoring with PET/CT was possible. They also found the highest discriminative value after the first cycle of NST with a reported sensitivity, specificity, negative predictive value and accuracy of FDG PET after one course of chemotherapy were, respectively, 96, 75, 95 and 84%. However, Jung et al. [109] found no correlation between the relative decrease in SUV peak and axillary response after NST in 66 patients. A limitation of response monitoring with sequential PET/CTs is that it may only be useful when FDG-avid axillary nodes are demonstrated prior to NST. Another limitation is the possibility of increasing FDG uptake of an axillary node due to infection or inflammation [106].

1.5 Safety of omitting axillary surgery among patients with a complete pathologic response in the breast and rationale for study design

It has long been shown by MD Anderson and other groups that the breast primary response with NST highly correlates with axillary lymph node response [110, 111]. Although it is well-known that axillary lymph node metastases respond well to chemotherapy with complete eradication of academic metastases in up to 75% of cases receiving anti-HER2 to directed chemotherapy, the protocol working group decided that the safest approach would include only patients with a clinically node-negative physical examination and ultrasound examination prior to beginning of NST or neoadjuvant endocrine and radiation therapy (NRT). This is based on our review of the Breast Cancer Management Database from MD Anderson among 527 recent patients treated between 2008 to the present who presented with T1 and T2 triple negative and HER2 positive evaluating NST and 510 patients with T1 estrogen receptor positive tumors treated with up front surgery. Among patients with HER2 positive disease, 100% of cases where a complete pathologic response (n=74) was seen in the primary tumor had no evidence of axillary lymph node metastases at surgery. Similarly, among 80 patients with triple negative breast cancer receiving NST who presented with a clinically node negative axilla, 99% of cases had no axillary metastases found during axillary surgery. In the one case where there was nodal metastases, it was seen in only one lymph node and it was felt that this could easily be covered with standard breast radiotherapy. It is important to keep in mind that this protocol will select cases for a complete pathologic response using image guided biopsy, therefore the group feels that this is a safe patient group where whole breast radiation and tangents will be sufficient in preventing local regional recurrence. On the other hand among similar patients with biopsy-proven axillary node-positive disease, patients with a complete pathologic response in the breast had residual disease in the axillary nodes in approximately 27% of cases. This data is the rationale for complete omission of surgery among cases with biopsy proved pCR in the breast among initial ultrasound N0 disease and allowing those patients with initial biopsy proved N1 disease the option of omission of breast surgery when biopsy shows a pCR in the breast and repeat axillary surgical staging as about 73% of these cases will also be able to minimize/de-escalate surgery.

MD Anderson data was queried to assist in defining a cohort of hormone receptor positive breast cancers for whom the risk of sentinel lymph node involvement was sufficiently low to enable comfort with omission of sentinel lymph node biopsy (in addition to omission of

lumpectomy). Among estrogen receptor positive breast cancers, including invasive ductal cancers in patients age ≥ 40 years, no lymphovascular space invasion and an Oncotype RS ≤ 25, clinically node negative, the incidence of having pathologically node positive disease by T stage was: T1mic: 0%; T1a: 0%; T1b: 5.3%; T1c 10.4%. Further subdividing patients, the highest risk of having occult lymph node metastases in clinically node negative disease was among patients age 40-49 with tumors 1.6-2.0 cm (incidence 18.5%), whereas for all other subgroups by age and size the incidence was ≤ 10%. For all subgroups, the majority of cases with lymph node positive disease had only one lymph node involved, in half of which it was a micrometastasis (<2mm).

The Oncotype genomic test is routinely used to assist with clinical decisions regarding the need for adjuvant chemotherapy in hormone receptor positive breast cancer, in the setting of both node positive and node negative disease. As such, Oncotype can be used to define a cohort of patients in whom the results of a sentinel lymph node biopsy would be unnecessary to make decisions regarding adjuvant systemic therapy. With the advent of biological tools such as the Oncotype RS, decisions regarding the use of adjuvant chemotherapy for early stage estrogen receptor positive breast cancers are no long made based on T and N stage characteristics. For example, the NSABP B-20 study demonstrated that for estrogen receptor positive tumors with a RS 0-10, there was no difference on distance disease free survival if chemotherapy was added to tamoxifen treatment [112]. The TAILORx study examined estrogen receptor positive tumors with a RS 11-15 and similarly found no benefit in distant disease free survival with the addition of chemotherapy to anti-endocrine therapy [113]. Indeed, in a preplanned analysis of the TAILORx study, even for young women ≤ 50 years of age, differences in 9 year distant disease free survival with or without chemotherapy were minimal with a RS 11-15 (98.0% vs. 97.2%) and with a RS 16-20 (95.2% vs. 93.6%). Hortobagyi and colleagues presented compelling data at the San Antonio Breast Cancer Symposium in 2018 examining 80,605 estrogen receptor positive breast cancers reported in SEER, including 4,335 with N1mic and 6182 with N1 disease [114]. They found that for patients with a RS<18, 9-year breast cancer specific survival was >97% regardless of nodal status. In this study, for patients with RS <25, the difference in BCSS with versus without chemotherapy for node positive patients was <1% at both 5 and 9 years. In sum, this data supports the omission of chemotherapy in patients with early stage, biologically favorable breast cancers, based on Oncotype results.

This data has been discussed by our working group as well as by the Breast Hormone Receptor Positive Working Group, both of which believe that using the Oncotype RS is a safe and clinically meaningful way to risk stratify patients regarding need of chemotherapy, even in the setting of potential occult disease in clinically node negative, estrogen receptor positive breast cancers. The data presented is the rationale for allowing patients with clinically node negative breast cancer treated with neoadjuvant radiation and anti-endocrine therapy to forego axillary surgery and chemotherapy.

1.6 Circulating tumor cells (CTCs)

Circulating tumor cells (CTCs) are subpopulations of cells within heterogeneous primary tumors that have acquired motility and invasive capabilities that facilitate invasion into the bloodstream. After they invade the bloodstream, they can travel to distant sites, remaining undetected and in a quiescent state for an extended period of time, before they establish distant metastases in the bone, lung, liver or brain.[115] Circulating tumor cell presence is not associated with tumor size, grade, subtype, or ≤3 positive lymph nodes, yet Lucci et al. have demonstrated that just one or

more CTCs/tube of blood are independent predictors of relapse in chemonaive patients [116] as well as patients who received NST prior to CTC assessment (Figure 1).[117] Circulating tumor cell identification rates were not different in chemonaive (24%) and NST treated patients (26%). These data are in agreement with a 2016 pooled analysis report that included 3100 patients.[118] In addition, Krishnamurthy, et al. have also shown discordance between HER2/neu status in primary tumor and CTCs; many patients with HER2 negative primary tumors have HER2 positive CTCs (Figure 2).[119] The ongoing TREAT-CTC trial (NCT01548677) for CTC positive operable patients with HER2-negative tumors, will provide valuable information on the utility of targeted therapies directed against CTCs. We have started obtaining serial blood draws and CTC assessments at 6 months and one year post-OP (Figure 3).

1.7 Plasma circulating tumor DNA (ctDNA)

Plasma circulating tumor DNA (ctDNA) is derived from tumor necrosis, tumor cell apoptosis and/or direct secretion of DNA by tumor cells. The blood is considered as a "reservoir" where ctDNA molecules, deriving from all tumor masses in a given patient, flow through. This allows the screening for tumor-associated genomic alterations directly from the plasma of patients. These alterations can be detected in the form of point mutations, copy number variations, chromosomal rearrangements or altered methylation patterns. Circulating tumor DNA assessment holds promise for providing critical information in the NST setting. During neoadjuvant treatment, radiological changes are often difficult to observe as imaging displays a limited resolution to clearly distinguish abnormal from normal tissues. Moreover, tumor biopsies used for molecular analyses are also difficult to carry out, especially after tumor shrinkage under NST. Unlike tumor analysis, assessing ctDNA in the plasma before and after NST may overcome these problems. With a short half-life in the blood stream, the potential changes in ctDNA levels are expected to be observed earlier than in those detectable on radiological images. This allows to monitor the tumor burden almost in real- time. Unlike tumor biopsies, ctDNA can be used to detect emerging tumor clones resistant to therapy and to adjust the therapy as soon as possible. We propose to identify ctDNA mutations by using digital sequencing (DS) in a collaboration with Guardant Health or a similar platform. Guardant Health's 360® digital sequencing technology is 1000 times more accurate at mutation detection than standard next generation sequencing methodologies, and obtaining the results takes half the time of standard tissue sequencing. We have already started analyzing triple negative patient plasma ctDNA samples using Guardant Health's DS platform and have preliminary data demonstrating feasibility of ctDNA mutation detection in triple negative and HER2 positive patients. We also have done work with Thermofisher on customized ctDNA panels for breast cancer patients. We will focus on the nine genetic mutations (*TP53*, *HER1/2*, *PIKC3A*, *MET*, *ALK*, *ATM*, *BRAF*, *NOTCH 1*, and *RB1*) that are most commonly detected in breast cancer patients (Figure 4).[120] We will perform ctDNA mutation analyses before and after NST, and at six months and one year post-OP to establish and validate gene mutation signatures that can be used to predict relapse.

Figure 1. The probability of relapse-free survival in patients with CTC count ≥1 following NST (Hazard ratio 2.68; 95% CI 1.45-4.97; log rank P < 0.002).

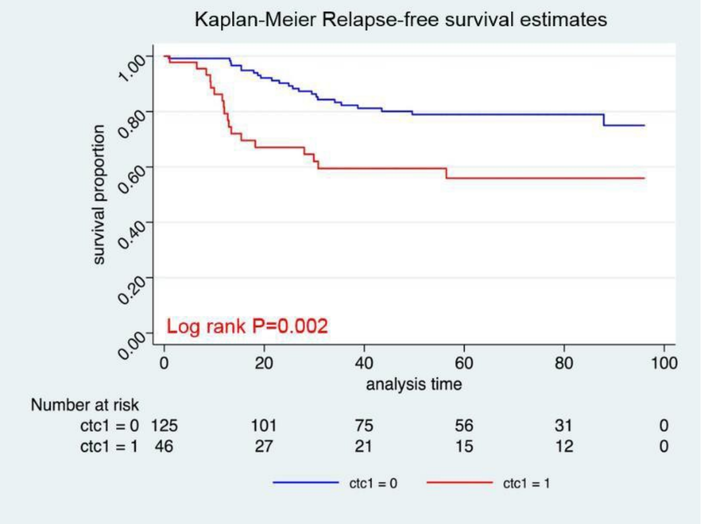

Figure 2. Circulating tumor cell HER2 measurement using FISH. Five of 79 (6.3%) patients with *HER2*-negative primary tumors had *HER2*-positive CTCs.

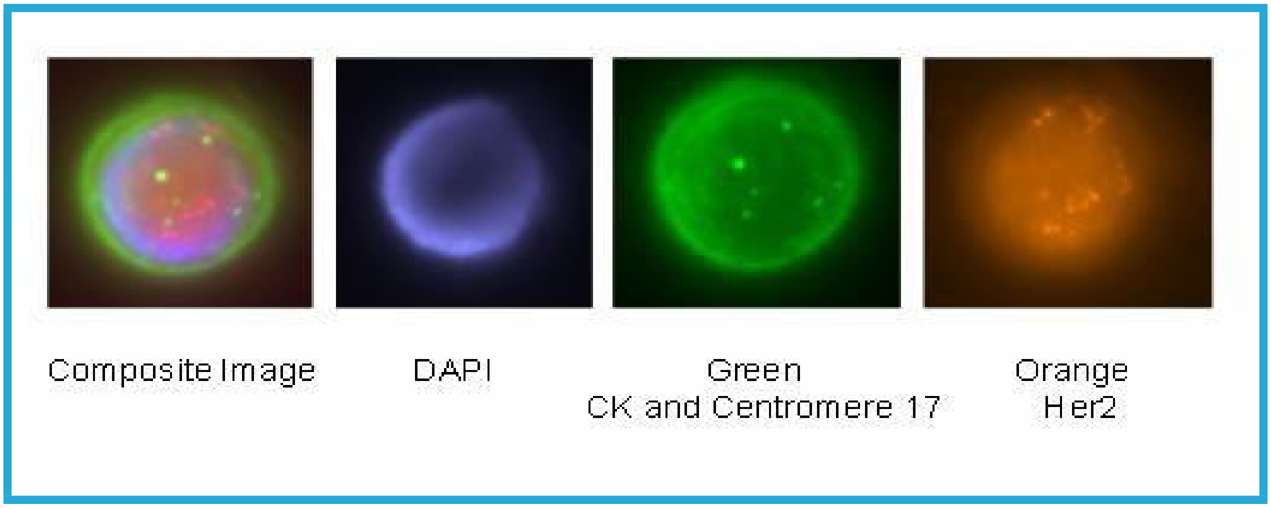

Figure 3. Serial CTC assessments. All relapses have been TNBC patients. BRST 11 and BRST 13 are ER+ with < 12 month FU. Might need more time to see progression

Acc #	Initial	6 Month	1 Year	
BRST-01	0-CTC	1-CTC		Patient relapsed and died
BRST-02	1-CTC	0-CTC		Patient relapsed
BRST-03	0-CTC		0-CTC	
BRST-04	0-CTC		113-CTC	Patient relapsed
BRST-05	0-CTC		2-CTC	Patient relapsed
BRST-06	0-CTC	0-CTC		
BRST-07	0-CTC	0-CTC	1-CTC	
BRST-08	0-CTC		0-CTC	
BRST-09	0-CTC	0-CTC		
BRST-10	0-CTC	0-CTC		
BRST-11	1-CTC	0-CTC		
BRST-12	2-CTC	0-CTC		Patient relapsed
BRST-13	2-CTC	0-CTC		

Figure 4. Nine mutations most commonly seen in breast cancer.

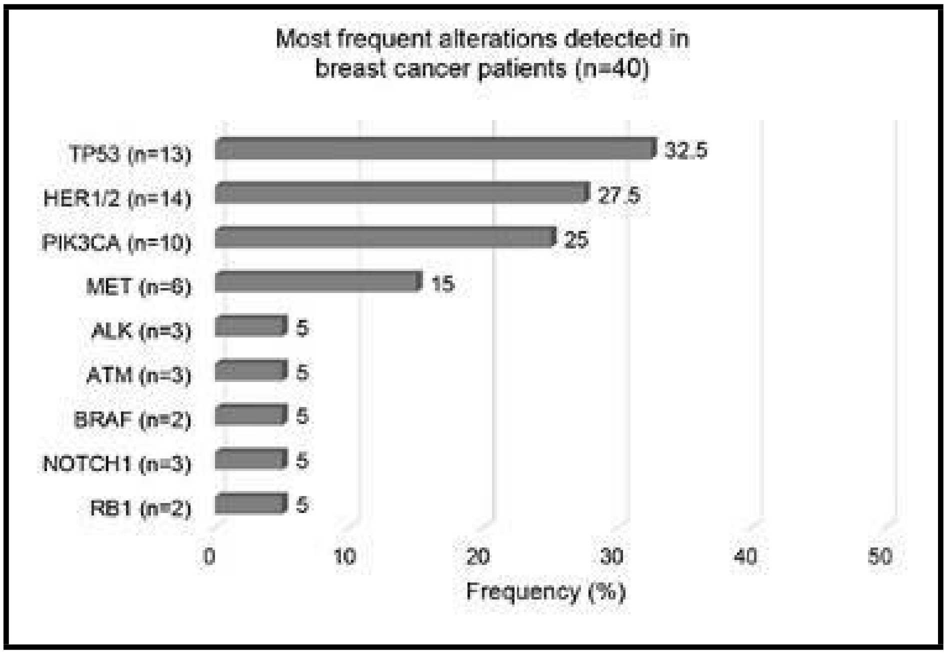

1.8 Genome-based tools to assess for response to radiation

Although breast tumors are generally responsive to radiation, there is a range in responsiveness even among breast tumors of the same biological subtype. There are efforts to utilize genomic-based tools to predict responsiveness of breast tumors to radiation. For example, investigators at the Moffitt Cancer Center have creased a validated clinical model that predicts responsiveness of tumors to radiotherapy. This tool, the genome-based model for adjusting radiotherapy dose (GARD), uses a gene-expression based radiation-sensitivity index together with the linear quadratic model [121]. Based on five distinct clinical cohorts, GARD was found to vary widely within tumor types but to also predict clinical outcome in breast cancer. This test includes 10 genes as a part of the radiosensitivity index (*AR, cJun, STAT1, PKC-beta, RelA, cABL, SUM01, PAK2, HDAC1,* and *IRF1*) and can be analyzed based on the FFPE specimen obtained at the time of biopsy. Similarly, the Adjuvant Radiotherapy Intensification Classifier (ARTIC) examines 27 genes and patient age to predict for benefit from radiation [122, 123] Whether or not GARD or ARTIC can predict response to ablative dose radiation in the setting of omission of surgery is unclear but is worth investigating as we aim to personalize local-regional treatment based on the biology of breast tumors. Scientists at MD Anderson are also similarly looking at genome-based tools specifically focused on DNA damage response and responsiveness to radiation. A patient cohort in whom radiation is given neoadjuvantly provides an ideal setting to explore if these radiosensitivity tests predict responsiveness to radiation.

1.9 Surgery alone for HER-2 positive patients

Historically, patients with HER-2 positive tumors had the highest rates of local recurrence, with rates of 10.8% at 5 years after lumpectomy and radiation [124]. However, with the advent of HER-2 directed therapy, local control and overall prognosis has greatly improved in this population. In a phase II study that enrolled patients with small (<3 cm) node negative, HER2 positive tumors treated with paclitaxel (80 mg/m2) weekly for 12 weeks plus trastuzumab for one year, the seven-year rate of local regional recurrence free survival was 99 percent in patients who received lumpectomy and radiation (95% CI 97.7-100) [125-Bellon]. This level of local control is higher than the early stage hormone positive population, for which there are multiple ongoing trials to look at recurrence rates with surgery alone in order to minimize overtreatment with radiation therapy, including IDEA (NCT02400190), PRECISION (NCT02653755), and EXPERT (NCT02889874). Therefore, there is a need to question the role of adjuvant radiation in HER-2 positive patients who are exceptional responders to systemic therapy.

2.0 Objectives

2.1 Primary

2.1.1 Cohort A: To determine the 6 mo, 1, 2, 3, and 5-year biopsy confirmed ipsilateral breast tumor recurrence rate (IBTR, invasive and/or in situ) among patients who do not undergo surgery.

2.1.2 Cohort B: To determine the pCR rate 6 months after radiation therapy based on image-guided biopsy.

2.1.3 Cohort C: To determine the 6 mo, 1, 2, 3, and 5-year ipsilateral breast tumor recurrence rate among patients who undergo surgery alone without radiation.

2.2 Secondary

2.2.1 Cohort B: To determine the 6 mo, 1, 2, 3, and 5-year biopsy confirmed ipsilateral breast tumor recurrence rate (IBTR, invasive and/or in situ) among patients who do not undergo surgery.

2.2.2 To determine the number (%) of patients where final biopsy reveals residual disease and quantify the residual disease (residual cancer burden, RCB) determined by routine pathologic examination of surgery specimens.

2.2.3 To assess baseline, 6 months, 1, 3, and 5 years decisional comfort of clinical trial participation using the Decisional Regret Scale (DRS).

2.2.4 To determine patient-reported cosmetic outcome, breast pain, and functional status using the Breast Cancer Treatment Outcomes Scale (BCTOS) at baseline, 6 months, 1, 3, and 5 years.

2.2.5 To determine the 6 mo, 1, 2, 3, and 5-year incidence of ipsilateral breast and nodal recommendation and performance of biopsy based on breast imaging follow-up.

2.2.6 Correlate “liquid biopsy” analyses (after standard routine NST, 6 months and one year post-radiotherapy or surgery) among protocol participants with pCR, utilizing circulating tumor cells (CTCs) and circulating tumor-DNA (ctDNA).

2.2.7 Among patients who decide to proceed with routine surgery, record the results of final biopsy compared with routine pathologic examination of surgery specimens.

2.2.8 To determine patient-reported quality of life using the FACT B+4 instrument at baseline, 6 months, 1, 3, and 5 years after treatment.

2.2.9 To explore if radiation genomic sensitivity scores and Oncotype performed on the initial diagnostic core biopsy specimen correlate with pCR rates in Cohort B.

2.2.10 To determine if changes in blood-based RNA Sequencing are elicited with radiation in Cohort B, measured at baseline, at the first 4-6 week follow-up after radiation, and at the 6 month post-radiation follow-up.

2.2.11 In Cohort B to determine the 3 year rate of tumor control/ progression free survival (PFS).

3.0 Patient Eligibility

3.1 Cohort A

- **Conditions for patient eligibility:** Patients on this portion of the study can receive radiation treatment at any MD Anderson Cancer Center or any outside hospital and may be enrolled prior to, during, or following neoadjuvant systemic therapy provided they meet the following eligibility and ineligibility requirements noted below:

3.1.1 Pathologically confirmed unicentric invasive breast cancer defined as radiologic clinical stage T1 or T2 (≤ 5 cm), N0 or N1 (≤ 4 abnormal axillary nodes on initial ultrasound), clinical stage M0.

3.1.2 HER2 positive (IHC 3+ and or FISH amplified) or triple receptor negative (TN, ER/PR< 10% HER2 negative (IHC 1+ or 2+ FISH non-amplified) receiving any standard routine clinical NST regimen.

3.1.3 Patient desires breast conserving therapy.

3.1.4 Age 40 years or older. This age cutoff is justified because breast cancers in women under the age of 40 are known to have a significantly higher risk of IBTR presumably due to underlying biologic differences [124, 125].

3.1.5 Female sex.

3.1.6 If the patient has a history of a prior non-breast cancer, all treatment for this cancer must have been completed prior to study registration and the patient must have no evidence of disease for this prior non-breast cancer.

3.1.7 Patient must have an initial nodal ultrasound that does not demonstrate more than four suspicious lymph nodes, any suspicious lymph nodes should be biopsied to determine if nodal metastatic disease present.

3.2 Cohort B

- **Conditions for patient eligibility:** Patients on this portion of the study will be limited to receive radiation treatment at MD Anderson Cancer Center and must be enrolled prior to any neoadjuvant systemic therapy provided they meet the following eligibility and ineligibility requirements noted below:

3.2.1 ER and/or PR positive, HER2 negative

3.2.2 Clinical stage T1N0M0, unicentric non-lobular breast cancer, no lymphovascular space invasion,

3.2.3 At least 40 years of age.

3.2.4 Oncotype ≤ 25 if age ≥ 50 years

3.2.5. Oncotype 0-20 and tumor size ≤ 1.5cm if age 40-49 years.

3.2.6 Patient agrees to take anti-estrogen therapy and is interested in breast conservation

3.2.7 Female sex.

3.2.8 If the patient has a history of a prior non-breast cancer, all treatment for this cancer must have been completed prior to study registration and the patient must have no evidence of disease for this prior non-breast cancer.

3.2.9 No history of prior radiation to the area of the breast that would require protocol-mandated treatment

3.3 Cohort C

- **Conditions for patient eligibility:** Patients on this portion of the study can receive surgical treatment at any MD Anderson Cancer Center or any outside hospital and may be enrolled prior to, during, or following neoadjuvant systemic therapy provided they meet the following eligibility and ineligibility requirements noted below:

3.3.1 Pathologically confirmed invasive breast cancer defined as radiologic clinical stage T1 or T2 (≤ 5 cm), N0, clinical stage M0.

3.3.2 HER2 positive (IHC 3+ and or FISH amplified) receiving any standard routine clinical NST regimen.

3.3.3 Patient desires breast conserving therapy.

3.3.4 Age 30 years or older.

3.3.5 Female sex.

3.3.6 If the patient has a history of a prior non-breast cancer, all treatment for this cancer must have been completed prior to study registration and the patient must have no evidence of disease for this prior non-breast cancer.

3.3.7 Patient must have an initial nodal ultrasound that does not demonstrate suspicious lymph nodes; any suspicious lymph nodes should be biopsied to determine if nodal metastatic disease present.

3.3.8 Patient must have no evidence of residual invasive tumor or DCIS on pathologic review of the lumpectomy surgical specimen

3.3.9 Patient must have no evidence of metastatic disease involving the lymph nodes on pathologic review of the lymph node surgical specimen.

3.3.10 Unifocal disease or limited multifocal disease that can be excised in a single lumpectomy specimen

3.4 Conditions for patient ineligibility for all cohorts

3.4.1 Radiologic evidence for a stage T3 or clinical stage T4 breast cancer in Cohort A/C; radiologic evidence for a stage T2-T3 or clinical stage T4 breast cancer in Cohort B.

3.4.2 Clinical or pathologic evidence for distant metastases.

3.4.3 Prior diagnosis of invasive or ductal carcinoma in situ breast cancer in the ipsilateral breast.

3.4.4 Clinical evidence of progression of disease >20% in the breast or new evidence of nodal metastases.

3.4.5 Patient is known to be pregnant.

3.4.6 Patient is participating in a NST protocol in which surgical excision of the breast and or lymph nodes are required in Cohort A/B.

Study Calendars

Cohort A

**Procedure/ Assessment**	**Visit 1** Baseline/Medical History	**Visit 2** 6 months (+/- 3 months) post Radiation Therapy	**Visit 3** 12 months (+/- 3 months) post Radiation Therapy
Inclusion/ Exclusion Criteria	X		
Informed Consent	X		
History & Physical Exam	X	X	X
Biopsy (2 to 6 weeks after neoadjuvant completion)	X		
Blood Draw*	X	X	X
**Imaging**			
Mammogram		X	X
MRI**			X

*For MD Anderson Houston Patients Only: One 10mL CellSave tube (lavender and yellow top) for CTC analysis; 2-10mL Streck tubes (black and tan top) for cDNA.
**Patients may undergo standard breast MRI alternating with mammography at 6 month periods at the discretion of their treatment team.

Long Term Follow- up †	Visit 4 18 months (+/- 3 months) post Radiation Therapy	Visit 5 24 months (+/- 3 months) post Radiation Therapy	Visit 6 30 months (+/- 3 months) post Radiation Therapy	Visit 7 36 months (+/- 3 months) post Radiation Therapy	Visit 8 42 months (+/- 3 months) post Radiation Therapy	Visit 9 48 months (+/- 3 months) post Radiation Therapy	Visit 10 54 months (+/- 3 months) post Radiation Therapy	Visit 11 60 months (+/- 3 months) post Radiation Therapy
History & Physical Exam	X	X	X	X	X	X	X	X
**Imaging**								
Mammogram	X	X	X	X	X	X	X	X
MRI**		X		X		X		X

**Patients may undergo standard breast MRI at alternating 6 month periods with mammography at the discretion of their treatment team.
† Patient/ and or their physicians may submit their routine standard history and physical examinations and imaging and or pathology reports if unable to come to their treating institutions for some of their follow-up examinations.

Cohort B

Patients will be treated with 3 months+/- 3 months of endocrine therapy, followed by ultrasound-guided evaluation. If there is ≥ 25% increase in size of the tumor on ultrasound at that time, then patients will proceed directly to surgery. If not, then patients will proceed with SABR followed by 6 months (+/- 1 month) endocrine therapy. Following this they will undergo image-guided biopsy. Patients may be evaluated for inclusion and exclusion criteria and consented at any time point prior to SABR.

If the biopsy is negative, then patients will receive additional endocrine therapy under the guidance of their medical oncologist. Regular breast surveillance imaging will be performed every 6 months. If the biopsy is positive then patients will proceed with breast conserving surgery. Endocrine therapy and regular breast imaging surveillance will be routine under the direction of the oncologic team.

Procedure/ Assessment	**Visit 1** Initial Evaluation	**Visit 2** 3 months (+/- 1 month) post Endocrine Therapy - Baseline Prior to RT	**Visit 3** 3-8 weeks post Radiation Therapy	**Visit 4** 6 months (+/- 1 month) post Radiation Therapy	**Visit 5** 12 months (+/-1) months) post Radiation Therapy
Inclusion/ Exclusion Criteria	X				
Informed Consent	X				
History	X				
Physical Exam	X		X	X	X
Blood Draw*	X***	X	X	X	X
Biopsy				X	

Imaging					
Mammogram				X	X
Ultrasound		X		X	X
MRI**					X

*For MD Anderson Houston Patients Only: One 10mL CellSave tube (lavender and yellow top) for CTC analysis; 2-10mL Streck tubes (black and tan top) for cDNA; one 10mL purple top tube
**Patients may undergo standard breast MRI alternating with mammography at 6 month periods at the discretion of their treatment team.
*** Optional pending patient availability physically on campus at MD Anderson Houston.

**Long Term Follow- up** †	**Visit 6** 18 months (+/- 3 months) post Radiation Therapy	**Visit 7** 24 months (+/- 3 months) post Radiation Therapy	**Visit 8** 30 months (+/- 3 months) post Radiation Therapy	**Visit 9** 36 months (+/- 3 months) post Radiation Therapy	**Visit 10** 42 months (+/- 3 months) post Radiation Therapy	**Visit 11** 48 months (+/- 3 months) post Radiation Therapy	**Visit 12** 54 months (+/- 3 months) post Radiation Therapy	**Visit 13** 60 months (+/- 3 months) post Radiation Therapy
History & Physical Exam	X	X	X	X	X	X	X	X
**Imaging**								
Mammogram	X	X	X	X	X	X	X	X
MRI**		X		X		X		X

**Patients may undergo standard breast MRI at alternating 6 month periods with mammography at the discretion of their treatment team.
† Patient/ and or their physicians may submit their routine standard history and physical examinations and imaging and or pathology reports if unable to come to their treating institutions for some of their follow-up examinations.

Cohort C

**Procedure/ Assessment**	**Visit 1** Baseline/Medical History	**Visit 2** 6 months (+/- 3 months) post Lumpectomy	**Visit 3** 12 months (+/- 3 months) post Lumpectomy
Inclusion/ Exclusion Criteria	X		
Informed Consent	X		
History & Physical Exam	X	X	X
Blood Draw*	X	X	X
**Imaging**			
Mammogram		X	X
MRI**			X

*For MD Anderson Houston Patients Only: One 10mL CellSave tube (lavender and yellow top) for CTC analysis; 2-10mL Streck tubes (black and tan top) for cDNA; one 10mL purple top tube.
**Patients may undergo standard breast MRI alternating with mammography at 6 month periods at the discretion of their treatment team.

**Long Term Follow- up** †	**Visit 4** 18 months (+/- 3 months) post Lumpectomy	**Visit 5** 24 months (+/- 3 months) post Lumpectomy	**Visit 6** 30 months (+/- 3 months) post Lumpectomy	**Visit 7** 36 months (+/- 3 months) post Lumpectomy	**Visit 8** 42 months (+/- 3 months) post Lumpectomy	**Visit 9** 48 months (+/- 3 months) post Lumpectomy	**Visit 10** 54 months (+/- 3 months) post Lumpectomy	**Visit 11** 60 months (+/- 3 months) post Lumpectomy
History & Physical Exam	X	X	X	X	X	X	X	X
**Imaging**								
Mammogram	X	X	X	X	X	X	X	X
MRI**		X		X		X		X

**Patients may undergo standard breast MRI at alternating 6 month periods with mammography at the discretion of their treatment team.

† Patient/ and or their physicians may submit their routine standard history and physical examinations and imaging and or pathology reports if unable to come to their treating institutions for some of their follow-up examinations.

4.0 Breast Biopsy Following NST

4.1 NST utilized by MD Anderson Breast Medical Oncology is highly standardized and individualized by the patient's tumor characteristics. Patients in this study may have any routine NST regimen recommended by their medical oncologist and all study participants will be eligible to avoid surgery if they have no pathologic evidence of disease at completion of NST. At the completion of NST patients will have their routine breast imaging and the radiologist will determine the best mechanism for biopsy. Most of the time the best breast imaging will be by stereotactic means with a minimum of 12 9G vacuum assisted biopsies. Biopsy will target the prior placed clip provided that there was no migration, and circumferentially of the remaining region which should be less than or equal to 2 cm and/or must include biopsy of any suspicious abnormality with > 90% of distortion/mass/residual microcalcifications removed. At the discretion of the radiologist, the breast biopsy can be performed by ultrasound-guided or MRI guided biopsy. The radiologist will place a marker clip in the area of the prior tumor to ensure that the areas marked for the surgeon if residual disease is obtained or for the radiation oncologist and for imaging follow-up when a pCR is identified.

5.0 Radiation Therapy

5.1 <u>Treatment/Dose Specifications</u>

5.1.1 External beam radiation therapy will be used exclusively in this study. Brachytherapy is not allowed.

5.1.2 Radiation therapy must begin within 12 weeks of completing neoadjuvant chemotherapy for Cohort A.

5.1.3 For clinically node-negative Cohort A patients, the prescription dose will be 40.05 Gy in 15 fractions delivered to the whole breast on consecutive

treatment days. A treatment day is defined as a normal business day, typically Monday – Friday excluding institutional holidays. The prescription dose for the tumor bed boost for patients assigned to HF- WBI will be 14 Gy in 7 fractions delivered on consecutive treatment days. The boost will begin on the treatment day following completion of whole breast irradiation.

5.1.4 For Cohort A patients whose axillary nodes were initially pathologically involved, the treating radiation oncologist may treat in accordance with section 5.1.3 above, or the treating radiation oncologist may treat the breast and draining nodal basins using a dose- fractionation of 50 Gy in 25 fractions followed by a tumor bed boost of 14 Gy in 7 fractions, all delivered on consecutive treatment days. The boost will begin on the treatment day following completion of whole breast irradiation.

5.1.5 For Cohort B patients, the prescription dose will be 37.5Gy in 5 fractions to the PTV_GTV and 28.5Gy in 5 fractions to the PTV_CTV delivered to the intact tumor every other day on business days. A normal business day is typically Monday-Friday, excluding institutional holidays. If treatment is delivered on a Friday, given the weekend break, the next treatment may be delivered on a Monday, at physician discretion.

5.1.6 In the event of severe acute toxicity in Cohort A during the course of radiotherapy (as determined by the treating physician), whole breast irradiation may be placed on hold for up to 3 treatment days and be replaced with the tumor bed boost to allow acute toxicity to subside. In this case, whole breast irradiation must resume upon discontinuation of the boost, and the total doses contributed from whole breast irradiation and the tumor bed boost will remain as stipulated in section 5.1.3.

5.1.7 The prescription points for whole breast irradiation, regional nodal irradiation if applicable, and the tumor bed boost will be selected at the discretion of the treating radiation oncologist to balance target coverage versus dose homogeneity.

5.1.8 Inhomogeneity corrections will be used in dose calculations.

5.2 Technical Factors

5.2.1 Radiation will be delivered using a linear accelerator with a nominal energy $\geq$ 6 MV.

5.2.2 Three-dimensional dose compensation will be used when needed to minimize dose inhomogeneity throughout the target volume using multileaf collimators and/or wedges.

5.2.3 Prone positioning may be utilized to improve normal tissue sparing when thought to be indicated by the treating radiation oncologist.

5.2.4 Respiratory gating with the deep inspiration breath hold technique may be

utilized to minimize exposure of the heart to radiation when thought to be indicated by the treating radiation oncologist.

5.3 Localization, Simulation, and Immobilization

5.3.1 The tumor bed will be located on the basis of the treatment planning computed tomography scan supplemented by preoperative imaging data when available.

5.3.2 Patients will undergo computed tomography-based simulation with an axial slice thickness no greater than 5 mm.

5.3.3 Patients will be immobilized using a breast board and vacuum-lock bag or other institution-specific immobilization techniques in standard use at the discretion of their treating physician.

5.4 Treatment Planning/Target Volumes for Cohort A

5.4.1 Whole breast irradiation will be planned and delivered with traditional tangent fields, with the superior border set at approximately the superior margin of the treated breast tissue, the medial border set at midline on the skin between the two breasts, the inferior border set 1-2 cm below in inframammary fold, and the lateral border set at approximately the mid-axillary line to ensure all palpable breast tissue is included within the fields. The posterior (deep) border of the medial and lateral tangent borders will be aligned to ensure that neither tangent field diverges into critical structures. A cardiac block may be utilized by the treating physician if it does not compromise coverage of the whole breast. Every effort should be made to minimize cardiac exposure.

5.4.2 For clinically and pathologically node-negative patients, treatment of the level I/II axillary lymph nodes with high tangent fields is allowed but not encouraged, as the risk of pathologic involvement of the low axilla is expected to be 2% or less based on institutional data. Addition of a third field to treat the supraclavicular, infraclavicular, or internal mammary lymph nodes is not allowed.

5.4.3 For clinically node-positive patients, treatment of the level I/II axillary lymph nodes, infraclavicular, supraclavicular, and internal mammary nodal basins is allowed at the discretion of the radiation oncologist. In this case, patients may be treated with any standard technique including partially wide tangents, electron-photon, intensity modulated radiation therapy, or volumetric arc therapy. All targeted nodal basins (with the exception of the supraclavicular nodes, should be contoured to generate clinical target volumes. At least 95% of each targeted nodal basins should be covered with a minimum dose of 45 Gy.

5.4.4 For the whole breast fields, the clinical target volume will be defined as biopsy clip(s) and adjacent architectural chances in breast parenchyma visualized on the treatment planning computed tomography scan. The clinical target volume will be trimmed such that it does not approach within 5 mm of the skin surface.

5.4.5 The tumor bed boost will be delivered with electrons or photons at the discretion of the treating radiation oncologist. The tumor bed boost will be delivered to the clinical target volume plus a radial margin of 2.0-3.0 cm at the discretion of the treating radiation oncologist. The minimum diameter of the tumor bed boost should be at least 6 cm. A second treatment planning computed tomography scan, with compression of the tumor bed or repositioning of the patient, may be obtained to assist in planning the tumor bed boost.

5.4.6 The treating physician should strive to minimize dose inhomogeneity within the breast. Any technique including the use of a wedge, three-dimensional dose compensation with a multi-left collimator, and/or intensity modulated radiation therapy is permitted. In treating the whole breast, use of an electron field matched to the tangent fields is allowed if needed to ensure adequate coverage of the tumor bed in the whole breast.

5.5 Treatment Planning/Target Volumes for Cohort B

5.5.1 Patient positioning will be performed based on the discretion of the treating radiation oncologist, who will determine this based on the patient's unique anatomy. An MRI for simulation purposes may be used as an adjunct to CT simulation. At attending discretion, four surface BB's may be placed on the breast at the time of simulation to assist with alignment for treatment delivery.

5.5.2 The attending physician will contour the gross tumor volume (GTV) in the treatment planning system. A planning target volume (PTV) will be created on this GTV by expanding 5mm (PTV_GTV). A uniform expansion of 2cm, subtracted off the chestwall musculature and within the first 0.5cm of the external skin surface, will be created to form the clinical target volume (CTV). An additional uniform margin of 0.5cm will be added to this to create the planning target volume (PTV_CTV). For the purposes of dosimetric coverage evaluative PTV (PTV_GTV_Eval and PTV_CTV_Eval) will be created, which is the PTV_GTV and PTV_CTV, respectively, subtracted 0.5cm from the skin surface and from the chestwall musculature.

5.5.3 Treatment may include photons, electrons, and/or proton radiation. Attempts should be made to keep the maximum dose to ≤ 120% of the prescription dose. 95% of the PTV_GTV_Eval should be covered by 99% of the prescription isodose line, Dmean 99-101%. 95% of the PTV_CTV should be covered by at least 99% of the prescription dose (Dmean 99-101%).

5.5.4 Prior to treatment delivery, the patient will be aligned to skin marks and 2D planar images (kV or MV) on the tumor bed, tumor bed clip(s), and/or surface BBs. 3D volumetric images including MRI with the MRLinac, CT on rails, or cone beam CT, will also be performed to verify patient position before radiation delivery as clinically indicated. Based on physician and physicist discretion, repeat imaging may be taken approximately mid-way through treatment delivery to verify patient positioning. If patient shifts are within 0.5 cm tolerance, then treatment will be continued. If not, then the patient will be re-imaged and re-positioned until within tolerance and treatment completed.

5.5.5 The treating physician should strive to minimize dose inhomogeneity within the breast. Any technique including the use of a wedge, three-dimensional dose compensation with a multi-left collimator, and/or intensity modulated radiation therapy is permitted.

5.6 Critical Structures

5.6.1 With respect to the lung, no more than 3 cm of lung shall be included in the tangent field as measured from the rib-lung interface to the deepest aspect of the tangent field.

5.6.2 The heart shall be excluded or the cardiac volume minimized from the tangent fields. This can be achieved through use of a cardiac block, deep inspiration breath hold, or other geometric means.

5.6.3 In Cohort B, the following dose constraints will be attempted.

- Heart V2.8 Gy <10%
- Heart V4.7 Gy<5%
- Mean Lung Dose <3.6 Gy
- Chestwall $D20_{cc}$<16.3 Gy
- Skin dose as low as possible; aim for D1cc<12 Gy; if this is not feasible aim for $D1_{cc}$<16 Gy
- Ipsilateral breast D_{mean} <5 Gy; PTV_CTV <25% total volume of ipsilateral breast
- Contralateral breast: dose as low as possible

5.7 Compliance Criteria

5.7.1 Plans will be considered acceptable if they adhere to the field borders as described in 5.4.1 and the clinical target volume is completely encompassed within the geometric projection of the tangent fields.

5.7.2 A treatment break of up to 3 treatment days is acceptable. A break of 4 or more days will be considered a deviation.

5.7.3 If the patient receives less than 90% of the intended whole breast radiation dose in Cohort A this will be considered unacceptable.

5.7.4 Tumor bed boost is mandatory in Cohort A.

5.8 Radiation Therapy Quality Assurance Reviews

All cases treated at MD Anderson will be presented for peer review in accordance with the policy of the Department of Radiation Oncology at The University of Texas MD Anderson Cancer Center. Patients treated at participating institutions will follow their specific quality assurance guidelines.

5.9 Radiation Adverse Events

Events that may occur include fatigue, skin erythema, dry or moist desquamation, hyperpigmentation, alopecia, tenderness, and swelling. Uncommon side effects include breast cellulitis or abscess and severe breast pain. In the long term, breast changes may occur including fibrosis, hyper- or hypo-pigmentation, telangiectasia, shrinkage/breast asymmetry, poor cosmesis, and edema. Uncommon side effects that may occur after completion of radiation include upper extremity lymphedema, rib fracture, pneumonitis, pulmonary fibrosis, pericarditis, ischemic heart disease, and heart failure. Adverse events on standard of care treatments will not be reported. Patients receiving partial breast irradiation will have side effects recorded, including erythema, desquamation, hyperpigmentation, pain, telangiectasis, cosmesis, rib fracture, and necrosis.

5.10 <u>Adverse Events and Serious Adverse Events Reporting Requirements</u>

5.10.1 The prompt reporting of adverse events is the responsibility of each investigator engaged in clinical research, as required by Federal Regulations. Toxicities/adverse events will be described and graded using the terminology and grading categories defined in the most current version of the NCI's Common Toxicity Criteria (CTCAE) version 4.0. The CTCAE is available at http://ctep.cancer.gov/reporting/ctc.html. Adverse events experienced by subjects will be collected from the time of the research biopsy and for 14 days following. Attribution to protocol treatment for each adverse event will be determined by the investigator and reported on the required forms.

5.10.2 All expected or unexpected adverse events related to the biopsy procedure, regardless of grade or treatment attribution, will be recorded on MD Anderson's departmental attribution log, in RedCap or in Prometheus, and that external sites should follow their local procedures for documenting their subject's adverse events.

5.10.3 This study is not a drug/biologics study. No expedited reporting of unrelated adverse events is required.

5.10.4 FNA/core biopsy is a routinely practiced procedure with expected adverse events including pain at needle placement/injection and rarely bruising/bleeding/hematoma.

5.10.5 Criteria and procedures for reporting Serious Adverse Events are described in the Data Quality Management Plan (Appendix K)

6.0 Data Monitoring/Confidentiality

6.1 The Principal Investigator will monitor the progress of the study and the safety of the participants.

6.2 This study will be conducted in accordance with all applicable privacy laws, rules, and regulations.

MD Anderson Subjects: The Principal Investigator, Study Co-Chairs, and the authorized research team will have access to the identifiable information from MD Anderson subjects entered into this study. Identifiers (such as name and medical record) will be collected but will be replaced by study numbers in the analytic file. The key linking these numbers will be retained in a secure computer file. Access to the MD Anderson subjects in this file will be limited to the Principal Investigator, Study Co- Chairs, and the authorized research team. All computer files are password-protected and stored on institution computers behind the institution firewall to further ensure database security and all records are kept confidential. Any reports or publications resulting from this study will not include any personal identifiers.

Multicenter Participating Institution Subjects: The Principal Investigator, and the authorized coordinating center monitors that ensure the quality of the conduct of the study will have access to the identifiable information from the multicenter participating institutions on this study. Initials and date of birth will be collected but will be replaced by study numbers in the analytic file. The key linking these numbers will be retained in a secure computer file. Access to the information from the participating multicenter institutions subjects in this file will be limited to the Principal Investigator, and the authorized coordinating center monitors. All computer files are password-protected and stored on institution computers behind the institution firewall to further ensure database security and all records are kept confidential. Any reports or publications resulting from this study will not include any personal identifiers. Additional information on the confidentiality related to participating multicenter institution's subjects can be found in the Data Quality Management Plan (Appendix K).

7.0 Surgery

Patients in Cohort A and B without histologic evidence of disease in the breast will not undergo breast surgery. All patients in this study with residual disease and all patients in Cohort C will undergo standard breast and nodal surgery. Patients with initial documented nodal disease and a breast pCR will have standard surgical staging of the axilla with standard targeted axillary dissection and completion dissection if residual nodal disease. Patients without initial documented nodal metastases may undergo standard sentinel lymph node biopsy if they deem clinically indicated. Per standard, surgical complications will be recorded.

8.0 Permitted Supportive Therapy

All supportive therapy indicated for optimal medical therapy during the course of standard therapy will be permitted for this study.

9.0 Tissue/Specimen Submission – For MD Anderson Houston Patients Only

1. Participating multicenter site patients are not required to contribute blood samples for this protocol.

2. We will collect blood (30mL) after NST before beginning radiation, 6 months post-radiation, and 1-year post-radiation. In Cohort B, 40ml blood will be collected before radiation, at first radiation follow-up and at 6 months and 1-year post-radiation visits. For each time point the following will be performed:
   - One 10 mL CellSave tube of blood will be used for CTC analysis. Circulating tumor cells will be enumerated and HER2 status will be determined using the CellSearch® system. Circulating tumor cell HER2 status will be measured for all patients, regardless of primary tumor HER2 status.
   - Two 10mL Streck tubes of blood will be shipped to Guardant Health or a similar platform for gene mutation analyses.
   - One 10mL purple top tube will be collected for such work as RNA sequencing.
3. Circulating tumor cell numbers and HER2 status throughout treatment and during follow up will be compared to determine if CTC values and HER2 status changes. Serial ctDNA gene mutation signature profiles will be established to determine if gene mutations arise from NST, and if specific genes predict relapse.

Blood procurement to correlate "liquid biopsy" analysis among protocol participants with pCR, utilizing CTCs and ctDNA. Blood samples will be drawn into tubes for each of the eligible subjects who grant consent. Blood samples will be hand delivered to Dr. Lucci's laboratory for CellSave and Streck tubes. Purple top tubes will be hand delivered to the Department of Radiation Oncology Biomarker Initiative Laboratory (PI Dr. Shaitelman). Preprinted labels with study identification numbers will be placed on each blood tube. A transmittal slip (multiple-copy form) will accompany each blood sample, and a copy will be retained in the Study Coordinator's office. All samples will be logged in TissueStation as per institutional requirements.

Recommendations for diagnostic core biopsies include fixation in formalin and routine processing and embedding in paraffin wax. One to two cores will be included in each cassette. Hematoxylin and eosin (H&E) stained tissue sections of the first and approximate tenth level of the paraffin blocks will be reviewed to determine the presence of residual carcinoma in situ and/or invasive carcinoma. When the first and approximate tenth H&E stained tissue sections are negative for residual tumor all the intervening unstained tissue sections of the paraffin blocks will be stained with H&E and reviewed to evaluate the tissue for presence of minimal amounts of residual tumor in the tissue. The core biopsies will be deemed to be entirely negative for residual tumor when all the ten sections from each block is negative for carcinoma in situ or invasive tumor. If tissue specimen is available per the collaborating pathologist to evaluate for genomic signatures of radiation responsiveness, exploratory studies may be performed to examine this.

10.0 Evaluation During Study

Patients will undergo weekly evaluations during radiation therapy, history and physical exam approximately 6 months (+/-3 months) after completing radiation therapy or surgery, and then every 6 months (+/-3 months) history and physical exam yearly for the 5 years following radiation therapy or surgery. The schedule for surveillance mammography is as follows: at the six month follow up visit then every 6 months for a total of 5 years. At the discretion of the treatment team, participants may undergo breast MRI at alternating 6-month periods. Patients may be followed more frequently at the discretion of the treatment team. After completing five years of follow up, patients will be off study and may be discharged to either the Institution's

Cancer Survivors Clinic or to their primary medical team.

Abnormal findings on mammography or clinical exam will be further investigated with additional imaging modalities such as ultrasound or other imaging as directed by the radiologist as per standard. Findings that remain suspicious after additional imaging should be biopsied to determine the presence or absence of ipsilateral breast tumor recurrence. Increase in size of calcifications and/or of residual mass/asymmetry, change in calcifications morphology (more aggressive morphology according to BIRADS lexicon), development of new asymmetry/mass should prompt biopsy as per standard criteria.

Patients deemed eligible for surgery will be taken off study.

10.1 Patient-Reported Outcome Evaluations (Appendix)

10.1.1 The FACT-B+4 is a validated 3-page 42 question instrument used to assess quality of life for women with breast cancer concerning physical well-being, social/family well- being, emotional well-being, and functional well-being. It will be used to assess general quality of life and will be scored according to standard guidelines that accompany the instrument.

10.1.2 Breast Cancer Treatment Outcome Scale (BCTOS) is a validated 1-page 22 question PROM survey that addresses patient cosmetic and functional outcome by comparing results to her contralateral breast and will be scored according to standard guidelines for this instrument.

10.1.3 Comfort with the baseline decision-making process will be assessed using the 6-item validated Decisional Regret Scale (DRS). Five subscales measure an individual's (1) uncertainty, (2) feeling uninformed, (3) value clarity, (4) support and (5) effective decision-making.

10.1.4 The FACT-B+4, BCTOS, and DRS will be administered at baseline and at the following timepoints measured from the date of radiation completion or surgery: 6 months, 1, 3, and 5 years (+/- 3 months annually). These questionnaires may be mailed to patients by regular mail (with provision of a self-addressed stamped envelope for return mail), or by encrypted email. Patients may also be contacted by telephone and asked the questions by the research staff and recorded for the patient.

**Patient Assessments**†	Baseline/Medical History	6 months post Radiation Therapy or Surgery	12 months post Radiation Therapy or Surgery	36 months post Radiation Therapy or Surgery	60 months post Radiation Therapy or Surgery
FACT-B+4	X*	X	X	X	X
BCTOS	X*	X	X	X	X
DRS	X*	X	X	X	X

†These questionnaires may be mailed to the patient by regular mail (with self-addressed stamped envelope for return mail), or by encrypted email.
*These may be collected at Visit 1 and/or Visit 2 for Cohort B as both are baseline prior to radiation therapy

10.2 Criteria for Removal from the Study

10.2.1 Criteria for Removal:

10.2.1.1 After the subject completes the 4th post radiation or surgery follow-up visit (5 years)

10.2.1.2 The patient may withdraw from the study at any time for any reason.

10.2.1.3 The subject's local study investigator determines that the subject cannot, or does not, adhere to the protocol plan.

10.2.1.4 Medical or psychiatric illness which in the investigator's judgment renders the patient incapable of further participation in the study.

10.2.2 All reasons for discontinuation from the study must be documented in the subject records and the study database.

11.0 Statistical Considerations

11.1 Cohort A & C

The eligible subjects for cohorts A and C are patients who have early stage breast cancer and achieve pathologic complete response (pCR) after neoadjuvant chemotherapy (NST). The Cohort A patients will receive definitive radiation therapy after they attain pCR without undergoing surgery, while the Cohort C patients will undergo surgery alone without receiving radiation if they attain pCR. The two cohorts will be run parallel, with each consisting of two phases. The first phase is to assess the feasibility of the corresponding treatment regimen. The second phase is to evaluate the ipsilateral breast tumor recurrence (IBTR, invasive and/or in situ) rate of the corresponding treatment regimen with longer follow-up.

11.1.1 Design and sample size/power

Based on historical data, it is anticipated that approximately 60% of patients will be found to have a pCR after NST demonstrated by image guided biopsy. Patients with residual disease on biopsy in Cohort A are not eligible to go on to definitive radiotherapy without standard surgery. Patients with residual disease on lumpectomy in Cohort C are not eligible to go on to definitive surgery without standard radiation. We will target approximately 50 total patients to enroll in each cohort in order to achieve 30 total patients (described below) with a pCR who will move on to definite radiotherapy or will receive surgery without radiation.

11.1.2 Feasibility Study Phase

Patients who are diagnosed with pathologically confirmed unicentric invasive breast cancer defined as clinical stage T1/T2 (≤ 5 cm on initial imaging), N0/N1 (≤ 4 abnormal nodes on initial ultrasound), M0 HER2 positive (both Cohort A and C) or triple receptor negative (Cohort A only) receiving any routine clinical NST regimen and who achieve a breast pCR after NST will be eligible to move on to

definitive radiotherapy (Cohort A) or to definitive surgery without radiotherapy (Cohort C). Patients with limited multifocal disease excised in a single lumpectomy specimen may also be eligible for Cohort C. Within each cohort, if none of the first 6 patients with pCR are to experience an IBTR during their first 6 months after they obtain a pCR, we would conclude that the corresponding proposed treatment regimen is feasible. If one or more patients out of the first patients (n≤6) have experienced IBTR anytime during the 6-month follow-up, the proposed treatment regimen (definitive radiation without surgery or definitive surgery without radiotherapy) may be considered unsafe, the corresponding cohort will be halted, and following review by the Co-PIs and the data monitoring group the corresponding cohort in this protocol will either be permanently closed or changed before considering continuation. If the true IBTR rate at 6- month for either of the treatment regimens is 2%, the probability to observe zero IBTR (out of 6 patients) within 6 months is 89%, and to observe ≥ 1 patient to have IBTR rate is 11%. If the true IBTR rate at 6-month is 15%, the corresponding probabilities are 38% and 62%, respectively.

11.1.3 Expansion Cohort Phase

The patients who are enrolled during the feasibility phase of each cohort will be rolled over into this phase. Patients will be continuously enrolled throughout the feasibility and expansion phase and enrollment to either of the two cohorts will cease if 1) any of the first 6 patients after 6-month follow-up experience an IBTR or 2) according to the formal stopping rules noted below. We will enroll additional patients whose final biopsy for NST reveals no residual disease, so that a total number of 30 patients who obtain pCR after NST will be included in this phase. The primary endpoint for this phase is ipsilateral breast tumor recurrence-free survival (IBT-RFS), defined as the time from confirmation of pCR to the time of ipsilateral breast tumor recurrence or death, whichever occurs first or the time of last contact.

We expect that definitive radiation without surgery or definitive surgery without radiation will have similar IBT-RFS compared to the current standard of care (SOC), i.e. surgery before definitive radiation. It is anticipated that patients will be enrolled at an accrual rate of 1 patient per month over 30 months for each cohort. When both cohorts are open for new patients, it will be at physician’s discretion which cohort a patient will be enrolled into once the patient gives consent to participate the study but without any preference to either of the two cohorts. Additional 60 months of follow-up is planned after the last patient is recruited into the trial. Assuming that IBT-RFS time follows an exponential distribution, and patients treated with SOC had a median IBT-RFS of 115 months corresponding to a 12-month IBTR rate of 7%.[126] We will monitor the IBT-RFS of the two cohorts separately using the method of Thall et al. [127] Let Ts and Te represent the IBT-RFS time for the standard care and definitive radiation without surgery or definitive surgery without radiation, respectively. We assume Ts|Ms and Te|Me follow an exponential distribution with respective median Ms and Me. Furthermore, we assume that the prior for Ms follows an inverse gamma distribution IG (1200, 137824) to reflect sufficient knowledge of IBT-RFS for the patients undergoing SOC. This has a mean of 115 months, and a variance of

11.0. The prior for Me is assumed to be IG (3, 300), which has the same mean of 115 months and a much larger variance of 22500 to reflect the uncertainty about the median IBT-RFS of the proposed treatment regimens.

The IBT-RFS will be monitored whenever an IBTR occurs or every six months. The corresponding cohort will be halted early if there is sufficient evidence based on the available data to show that the median IBT-RFS of the patients treated with SOC is longer than that of the patients treated with definitive radiation without surgery or definitive surgery without radiation, and following review by the Co-PIs and data monitoring group the protocol will either be permanently closed or changed before considering continuation.

The formal stopping rule is Pr(Ms > Me | data) > 0.70

Specifically, the cohort (either A or C) will be stopped early if there is greater than 70% chance that the median IBT-RFS of the patients treated with SOC is longer than that of the patients treated with definitive radiation without surgery or definitive surgery without radiation. The probability cutoff of 0.70 was chosen to obtain a reasonable and safe early stopping probability if the true median IBT-RFS for definitive radiation without surgery or definitive surgery without radiation is indeed shorter than the historical median IBT-RFS of 115 months, for example, if the true median IBT-RFS is 96 months, the early stopping probability is 69.4%.

The operating characteristics of this decision rule are summarized in Table 1 using the onearmTTE software developed at the Department of Biostatistics at M.D. Anderson.

Table 1. Operating characteristics for the design (based on 2000 simulations)

True median ipsilateral breast tumor recurrence-free survival (IBT-RFS) (month)	Pr(stop early)	Average Number of Patients Treated (25th, 75th percentiles)
60	0.982	17.7 (9, 30)
72	0.932	19.3 (10, 30)
84	0.824	20.6 (11, 30)
96	0.694	21.7 (11, 30)
108	0.580	22.6 (12, 30)
115	0.526	23.0 (12, 30)
120	0.487	23.2 (13, 30)

This monitoring will be carried out via the Clinical Trial Conduct (CTC) website (https://biostatistics.mdanderson.org/ClinicalTrialConduct) which is housed on a secure server at MDACC and maintained by the MDACC Department of Biostatistics. The protocol PI or his designated research personnel, will enter the required variables into CTC for all subjects in Cohort A and C, respectively.

Access to the website will be gained through a username and password provided by the MDACC Department of Biostatistics. Training on the use of the CTC will be provided by the biostatistical collaborator of the study, with emphasis on the importance of timely updating of follow-up times and recording of events. The monitoring rules for the IBT-RFS will be applied every six months or whenever an IBTR occurs with the probability criterion recomputed based on the most recent data available at that time. If the stopping rule is met, the MD Anderson study statistician, MD Anderson research nurse, the Multicenter Study Support's (MCSS) coordinating center team, the Cancer Network coordinating center team and study principal investigator, Dr. Kuerer, will each receive an email notification that the stopping boundary has been met. Participating Investigators outside MD Anderson will be notified by the MCSS coordinating center or Cancer Network coordinating center, of any study suspensions, closures, holds resulting from the statistical monitoring.

The analyses will be conducted separately for Cohort A and C. Patients' demographic and clinical characteristics at baseline will be summarized using descriptive statistics such as frequency distribution, mean (± s.d.) and median (range) accompanied by graphical analysis. Student t-test/Wilcoxon test and ANOVA/Kruskal-Wallis test will be used to compare continuous variables between different patient groups. The chi-square test or the Fisher's exact test will be applied to assess the association between two categorical variables.[128]

Time-to-event outcomes, including IBT-RFS and overall survival (OS), will be estimated using the Kaplan-Meier method[129] log-rank test will be performed to test the difference in time-to- event distributions between patient groups.[130] Cox proportional hazards model will be used to include multiple covariates in the time-to-event analysis.[131]

Exploratory data analysis and graphical methods will be applied to examine distributions of the biomarker data, DRS, BIS, FACT B+4, CTS and plasma ctDNA data, and to identify data error and outliers. Range check and consistency check will be applied to ensure the data quality. Standard distribution plots such as the histogram and box-plot will be applied. A more versatile BLiP plot[132] will be used to facilitate the plotting of both discrete and continuous data. T-test/ANOVA or their nonparametric counterparts, Wilcoxon rank- sum test/Kruskal- Wallis test (Wilson and Clarke, 2000) will be used to detect differences of the aforementioned data between groups. Since the data will be measured at baseline and multiple post NST time points. Linear mixed effect models for repeated measures analysis[133] will be employed to assess the change of the data over time with multi- covariates including disease characteristics (tumor stage, site, pathology), and other patient prognostic factors. Appropriate transformation of the outcome assessment values will be used to satisfy the normality assumption of linear mixed effect model. For the longitudinal binary endpoint, incidence of ipsilateral breast and nodal recommendation and performance of biopsy based on breast imaging follow-up (objective 2.2.4), multivariable logistic regression analysis using generalized estimating equations (GEE) to take the intra- patient correlation into account will be used to determine factors significantly associated with the outcome.[134]

Descriptive statistics will be used to summarize RCB. Among patients who have residual disease on final biopsy and who with routine surgery, the final biopsy will be compared with the response status determined by routine pathologic examination of surgery specimens using McNemar test. Additional analyses in furtherance of the stated objectives may be conducted as warranted.

11.2. Cohort B

The eligible subjects for this study are patients who have early stage breast cancer and receive SABR to the intact breast tumor. The purpose of this study is to evaluate the rate of pCR as assessed by biopsy following SABR + 6 months of endocrine therapy.

11.2.1 Design and Sample Size/Power

Based on historical data, it is anticipated that approximately 50% of patients will be found to have a pCR after neoadjuvant SABR. Patients with residual disease on biopsy will proceed with standard surgery. We will target approximately 20 total patients to enroll in Cohort B in order to explore the rate of pCR. Patients who withdraw consent to protocol treatment and biopsy and/or become ineligible prior to receiving any study related radiation treatment (full or partial dose) will be replaced.

If there is a relatively low probability that the pCR rate is likely to be > 30%, the proposed treatment would not be considered of interest for further study. Formally if Pr(pCR rate > 0.30 | Data) < 0.30, the cohort will be stopped. The assumed prior distribution for pCR rate is Beta (0.3, 0.7). The resulting prior mean is 0.30 for pCR rate with one patient worth of information. The cohort will be monitored in cohorts of 10 patients after a minimum of 10 patients have completed the biopsy following SABR + 6 months of endocrine therapy. Consideration will be given to stop or amend the cohort if <3 patients have a pCR per cohort of 10 evaluated. Table 2 shows the operating characteristics of the corresponding design.

Table 2. Operating characteristics for the stopping rule for pCR monitoring within Cohort B

True pCR Rate	Probability Stop Early	Average No. of patients treated
0.10	0.93	10.7
0.20	0.68	13.2
0.30	0.38	16.2
0.40	0.17	18.3
0.50	0.055	19.5
0.60	0.012	19.9

If the cohort is stopped early, this proposed treatment will not be considered of

interest for future study for this cohort of patients. If all 20 patients are accrued, we will estimate pCR rate and the corresponding 95% posterior credible interval. For example, if the proposed treatment has a true pCR rate of 55% and 11 patients who achieved pCR were observed, then the 95% credible interval would be (0.33, 0.74). Moreover, 3-year tumor control rate or progression-free survival (PFS) rate is considered as an important clinical efficacy measure. PFS is defined as the time from initiation of SABR to the time of ipsilateral breast tumor progression or death, whichever occurs first or the time of last contact. We will estimate 3-year PFS rate using Kaplan-Meir method along with 95% confidence interval. The treatment would be considered promising for further investigation if 2 or less among the 20 patients experienced tumor progression given 3-year follow-up. If the true 3-year PFS rate is 90%, the probability to observe 3 or more patients experiencing progression (out of 20 patients) within 3 years is 32%, and to observe ≤ 2 patients to have progression is 68%. If the true 3-year PFS rate is 70%, the corresponding probabilities are 96% and 4%, respectively. We will apply appropriate methods to analyze the secondary and exploratory endpoints related to this cohort.

12.0 Data and Quality Management

12.1 In addition to the statistical monitoring previously described in this study the data and quality management will be overseen by the Clinical Research Support Center's Multicenter Study Support (MCSS), Cancer Network coordinating team, and Audit teams, as described in the attached Data Quality Management Plan (DQMP) (Appendix K). MD Anderson departmental staff will adhere to the protocol and conduct the study in accordance with institutional and departmental policies and procedures.

12.2 The DQMP also describes the general procedures that document the administrative structure of this multicenter collaboration. This information includes but may not be limited to a description of the:

12.2.1 Roles and responsibilities of the lead Investigator, Dr. Kuerer, the participating site's, participating Investigators, and participating research teams, as well as the CRSC coordinating center teams.

12.2.2 Protocol development, protocol management, and data management processes (for example communication flow, regulatory requirements, confidentiality information data reporting) etc.

12.2.3 Requirements for site activation prior to screening and enrolling subjects.

12.2.4 Procedures for registering subjects and a description of the data and source document submission expectations for this study.

12.2.5 Definitions of, and instructions for reporting, serious adverse events, unanticipated problems, protocol deviations and protocol violations.

12.2.6 Quality assurance mechanisms and the consequences of poor performance.

13.0 REFERENCES

1. Induction chemotherapy plus radiation compared with surgery plus radiation in patients with advanced laryngeal cancer. The Department of Veterans Affairs Laryngeal Cancer Study Group. *N Engl J Med* 1991, 324(24):1685-1690.
2. Bartelink H, Roelofsen F, Eschwege F, Rougier P, Bosset JF, Gonzalez DG, Peiffert D, van Glabbeke M, Pierart M: Concomitant radiotherapy and chemotherapy is superior to radiotherapy alone in the treatment of locally advanced anal cancer: results of a phase III randomized trial of the European Organization for Research and Treatment of Cancer Radiotherapy and Gastrointestinal Cooperative Groups. *J Clin Oncol* 1997, 15(5):2040-2049.
3. Cooper JS, Guo MD, Herskovic A, Macdonald JS, Martenson JA, Jr., Al-Sarraf M, Byhardt R, Russell AH, Beitler JJ, Spencer S *et al*: Chemoradiotherapy of locally advanced esophageal cancer: long- term follow-up of a prospective randomized trial (RTOG 85-01). Radiation Therapy Oncology Group. *JAMA* 1999, 281(17):1623-1627.
4. Furuse K, Fukuoka M, Kawahara M, Nishikawa H, Takada Y, Kudoh S, Katagami N, Ariyoshi Y: Phase III study of concurrent versus sequential thoracic radiotherapy in combination with mitomycin, vindesine, and cisplatin in unresectable stage III non- small-cell lung cancer. *J Clin Oncol* 1999, 17(9):2692-2699.
5. Lefebvre JL, Chevalier D, Luboinski B, Kirkpatrick A, Collette L, Sahmoud T: Larynx preservation in pyriform sinus cancer: preliminary results of a European Organization for Research and Treatment of Cancer phase III trial. EORTC Head and Neck Cancer Cooperative Group. *J Natl Cancer Inst* 1996, 88(13):890-899.
6. Morris M, Eifel PJ, Lu J, Grigsby PW, Levenback C, Stevens RE, Rotman M, Gershenson DM, Mutch DG: Pelvic radiation with concurrent chemotherapy compared with pelvic and para-aortic radiation for high-risk cervical cancer. *N Engl J Med* 1999, 340(15):1137-1143.
7. Landoni F, Maneo A, Colombo A, Placa F, Milani R, Perego P, Favini G, Ferri L, Mangioni C: Randomised study of radical surgery versus radiotherapy for stage Ib-IIa cervical cancer. *Lancet* 1997, 350(9077):535-540.
8. Touboul E, Buffat L, Lefranc JP, Blondon J, Deniaud E, Mammar H, Laugier A, Schlienger M: Possibility of conservative local treatment after combined chemotherapy and preoperative irradiation for locally advanced noninflammatory breast cancer. *Int J Radiat Oncol Biol Phys* 1996, 34(5):1019- 1028.
9. Mauriac L, MacGrogan G, Avril A, Durand M, Floquet A, Debled M, Dilhuydy JM, Bonichon F: Neoadjuvant chemotherapy for operable breast carcinoma larger than 3 cm: a unicentre randomized trial with a 124-month median follow-up. Institut Bergonie Bordeaux Groupe Sein (IBBGS). *Ann Oncol* 1999, 10(1):47-52.
10. Scholl SM, Pierga JY, Asselain B, Beuzeboc P, Dorval T, Garcia-Giralt E, Jouve M, Palangie T, Remvikos Y, Durand JC *et al*: Breast tumour response to primary chemotherapy predicts local and distant control as well as survival. *Eur J Cancer* 1995, 31A(12):1969-1975.
11. Perloff M, Lesnick GJ, Korzun A, Chu F, Holland JF, Thirlwell MP, Ellison RR, Carey RW, Leone L, Weinberg V *et al*: Combination chemotherapy with mastectomy or radiotherapy for stage III breast carcinoma: a Cancer and Leukemia Group B study. *J Clin Oncol* 1988, 6(2):261-269.
12. Daveau C, Savignoni A, Abrous-Anane S, Pierga JY, Reyal F, Gautier C, Kirova YM, Dendale R, Campana F, Fourquet A *et al*: Is radiotherapy an option for early breast cancers with complete clinical response after neoadjuvant chemotherapy? *Int J Radiat Oncol Biol Phys* 2011, 79(5):1452- 1459.
13. De Lena M, Varini M, Zucali R, Rovini D, Viganotti G, Valagussa P, Veronesi U, Bonadonna

G: Multimodal treatment for locally advanced breast cancer. Result of chemotherapy-radiotherapy versus chemotherapy-surgery. *Cancer Clin Trials* 1981, 4(3):229-236.
14. Ellis P, Smith I, Ashley S, Walsh G, Ebbs S, Baum M, Sacks N, McKinna J: Clinical prognostic and predictive factors for primary chemotherapy in operable breast cancer. *J Clin Oncol* 1998, 16(1):107- 114.
15. Ring A, Webb A, Ashley S, Allum WH, Ebbs S, Gui G, Sacks NP, Walsh G, Smith IE: Is surgery necessary after complete clinical remission following neoadjuvant chemotherapy for early breast cancer? *J Clin Oncol* 2003, 21(24):4540-4545.
16. Clouth B, Chandrasekharan S, Inwang R, Smith S, Davidson N, Sauven P: The surgical management of patients who achieve a complete pathological response after primary chemotherapy for locally advanced breast cancer. *Eur J Surg Oncol* 2007, 33(8):961-966.
17. Mauri D, Pavlidis N, Ioannidis JP: Neoadjuvant versus adjuvant systemic treatment in breast cancer: a meta-analysis. *J Natl Cancer Inst* 2005, 97(3):188-194.
18. Ellis MJ, Suman VJ, Hoog J, et al. Randomized phase II neoadjuvant comparison between letrozole, anastrozole, and exemestane for postmenopausal women with estrogen receptor-rich stage 2 to 3 breast cancer: clinical and biomarker outcomes and predictive value of the baseline PAM50-based intrinsic subtype--ACOSOG Z1031. *J Clin Oncol.* 2011;29(17):2342-2349.
19. Akashi-Tanaka S, Shimizu C, Ando M, et al. 21-Gene expression profile assay on core needle biopsies predicts responses to neoadjuvant endocrine therapy in breast cancer patients. *Breast.* 2009;18(3):171-174.
20. Ueno T, Masuda N, Yamanaka T, et al. Evaluating the 21-gene assay Recurrence Score(R) as a predictor of clinical response to 24 weeks of neoadjuvant exemestane in estrogen receptor-positive breast cancer. *Int J Clin Oncol.* 2014;19(4):607-613.
21. Timmerman R, Paulus R, Galvin J, et al. Stereotactic body radiation therapy for inoperable early stage lung cancer. *JAMA.* 2010;303(11):1070-1076.
22. Tao R, Krishnan S, Bhosale PR, et al. Ablative Radiotherapy Doses Lead to a Substantial Prolongation of Survival in Patients With Inoperable Intrahepatic Cholangiocarcinoma: A Retrospective Dose Response Analysis. *J Clin Oncol.* 2016;34(3):219-226.
23. Robin TP, Raben D, Schefter TE. A Contemporary Update on the Role of Stereotactic Body Radiation Therapy (SBRT) for Liver Metastases in the Evolving Landscape of Oligometastatic Disease Management. *Semin Radiat Oncol.* 2018;28(4):288-294.
24. Chang EL, Shiu AS, Mendel E, et al. Phase I/II study of stereotactic body radiotherapy for spinal metastasis and its pattern of failure. *J Neurosurg Spine.* 2007;7(2):151-160.
25. Ho JC, Tang C, Deegan BJ, et al. The use of spine stereotactic radiosurgery for oligometastatic disease. *J Neurosurg Spine.* 2016;25(2):239-247.
26. Timmerman RD, Paulus R, Pass HI, et al. Stereotactic Body Radiation Therapy for Operable Early-Stage Lung Cancer: Findings From the NRG Oncology RTOG 0618 Trial. *JAMA Oncol.* 2018;4(9):1263-1266.
27. Nagata Y, Hiraoka M, Shibata T, et al. Prospective Trial of Stereotactic Body Radiation Therapy for Both Operable and Inoperable T1N0M0 Non-Small Cell Lung Cancer: Japan Clinical Oncology Group Study JCOG0403. *Int J Radiat Oncol Biol Phys.* 2015;93(5):989-996.
28. Chang JY, Senan S, Paul MA, et al. Stereotactic ablative radiotherapy versus lobectomy for operable stage I non-small-cell lung cancer: a pooled analysis of two randomised trials. *Lancet Oncol.* 2015;16(6):630-637.
29. Nichols E, Kesmodel SB, Bellavance E, et al. Preoperative Accelerated Partial Breast Irradiation for Early-Stage Breast Cancer: Preliminary Results of a Prospective, Phase 2 Trial. *Int J Radiat Oncol Biol Phys.* 2017;97(4):747-753.
30. Bosma S, Elkhuizen P, Bartelink H, Vijver M vd. Response to Pre-Operative Radiotherapy in Relation to Gene Expression Patterns in Breast Cancer Patients. *ASTRO's 59th Annual*

Meeting. October 2017.
31. Vasmel J CR, Houweling A, Philippens M, et al. MR-guided single dose pre-operative radiotherapy in low-risk breast cancer patients: first results. *European Society for Radiation Oncology 37 Congress*, 2018.
32. Boughey JC, McCall LM, Ballman KV, Mittendorf EA, Ahrendt GM, Wilke LG, Taback B, Leitch AM, Flippo-Morton T, Hunt KK: Tumor biology correlates with rates of breast-conserving surgery and pathologic complete response after neoadjuvant chemotherapy for breast cancer: findings from the ACOSOG Z1071 (Alliance) Prospective Multicenter Clinical Trial. *Ann Surg* 2014, 260(4):608-614; discussion 614-606.
33. Eisenhauer EA, Therasse P, Bogaerts J, Schwartz LH, Sargent D, Ford R, Dancey J, Arbuck S, Gwyther S, Mooney M *et al*: New response evaluation criteria in solid tumours: revised RECIST guideline (version 1.1). *Eur J Cancer* 2009, 45(2):228- 247.
34. Chagpar AB, Middleton LP, Sahin AA, Dempsey P, Buzdar AU, Mirza AN, Ames FC, Babiera GV, Feig BW, Hunt KK *et al*: Accuracy of physical examination, ultrasonography, and mammography in predicting residual pathologic tumor size in patients treated with neoadjuvant chemotherapy. *Ann Surg* 2006, 243(2):257-264.
35. Esserman L, Hylton N, Yassa L, Barclay J, Frankel S, Sickles E: Utility of magnetic resonance imaging in the management of breast cancer: evidence for improved preoperative staging. *J Clin Oncol* 1999, 17(1):110-119.
36. Herrada J, Iyer RB, Atkinson EN, Sneige N, Buzdar AU, Hortobagyi GN: Relative value of physical examination, mammography, and breast sonography in evaluating the size of the primary tumor and regional lymph node metastases in women receiving neoadjuvant chemotherapy for locally advanced breast carcinoma. *Clin Cancer Res* 1997, 3(9):1565-1569.
37. Londero V, Bazzocchi M, Del Frate C, Puglisi F, Di Loreto C, Francescutti G, Zuiani C. Locally advanced breast cancer: comparison of mammography, sonography and MR imaging in evaluation of residual disease in women receiving neoadjuvant chemotherapy. *Eur Radiol* 2004, 14(8):1371-1379.
38. Mumtaz H, Davidson T, Spittle M, Tobias J, Hall-Craggs MA, Cowley G, Taylor I: Breast surgery after neoadjuvant treatment. Is it necessary? *Eur J Surg Oncol* 1996, 22(4):335-341.
39. Prati R, Minami CA, Gornbein JA, Debruhl N, Chung D, Chang HR: Accuracy of clinical evaluation of locally advanced breast cancer in patients receiving neoadjuvant chemotherapy. *Cancer* 2009, 115(6):1194-1202.
40. Segara D, Krop IE, Garber JE, Winer E, Harris L, Bellon JR, Birdwell R, Lester S, Lipsitz S, Iglehart JD *et al*: Does MRI predict pathologic tumor response in women with breast cancer undergoing preoperative chemotherapy? *J Surg Oncol* 2007, 96(6):474-480.
41. Tsuboi N, Ogawa Y, Inomata T, Yoshida D, Yoshida S, Moriki T, Kumon M: Changes in the findings of dynamic MRI by preoperative CAF chemotherapy for patients with breast cancer of stage II and III: pathologic correlation. *Oncol Rep* 1999, 6(4):727- 732.
42. Wright FC, Zubovits J, Gardner S, Fitzgerald B, Clemons M, Quan ML, Causer P: Optimal assessment of residual disease after neo-adjuvant therapy for locally advanced and inflammatory breast cancer--clinical examination, mammography, or magnetic resonance imaging? *J Surg Oncol* 2010, 101(7):604-610.
43. Yeh E, Slanetz P, Kopans DB, Rafferty E, Georgian-Smith D, Moy L, Halpern E, Moore R, Kuter I, Taghian A: Prospective comparison of mammography, sonography, and MRI in patients undergoing neoadjuvant chemotherapy for palpable breast cancer. *AJR Am J Roentgenol* 2005, 184(3):868-877.
44. Huber S, Medl M, Vesely M, Czembirek H, Zuna I, Delorme S: Ultrasonographic tissue characterization in monitoring tumor response to neoadjuvant chemotherapy in locally advanced breast cancer (work in progress). *J Ultrasound Med* 2000, 19(10):677-686.

45. Peintinger F, Kuerer HM, Anderson K, Boughey JC, Meric-Bernstam F, Singletary SE, Hunt KK, Whitman GJ, Stephens T, Buzdar AU *et al*: Accuracy of the combination of mammography and sonography in predicting tumor response in breast cancer patients after neoadjuvant chemotherapy. *Ann Surg Oncol* 2006, 13(11):1443-1449.
46. Keune JD, Jeffe DB, Schootman M, Hoffman A, Gillanders WE, Aft RL: Accuracy of ultrasonography and mammography in predicting pathologic response after neoadjuvant chemotherapy for breast cancer. *Am J Surg* 2010, 199(4):477-484.
47. Schott AF, Roubidoux MA, Helvie MA, Hayes DF, Kleer CG, Newman LA, Pierce LJ, Griffith KA, Murray S, Hunt KA *et al*: Clinical and radiologic assessments to predict breast cancer pathologic complete response to neoadjuvant chemotherapy. *Breast Cancer Res Treat* 2005, 92(3):231-238.
48. Croshaw R, Shapiro-Wright H, Svensson E, Erb K, Julian T: Accuracy of clinical examination, digital mammogram, ultrasound, and MRI in determining postneoadjuvant pathologic tumor response in operable breast cancer patients. *Ann Surg Oncol* 2011, 18(11):3160-3163.
49. Schaefgen B, Mati M, Sinn HP, Golatta M, Stieber A, Rauch G, Hennigs A, Richter H, Domschke C, Schuetz F *et al*: Can Routine Imaging After Neoadjuvant Chemotherapy in Breast Cancer Predict Pathologic Complete Response? *Ann Surg Oncol* 2015.
50. Corcioni B, Santilli L, Quercia S, Zamagni C, Santini D, Taffurelli M, Mignani S: Contrast-enhanced US and MRI for assessing the response of breast cancer to neoadjuvant chemotherapy(). *J Ultrasound* 2008, 11(4):143-150.
51. Evans A, Armstrong S, Whelehan P, Thomson K, Rauchhaus P, Purdie C, Jordan L, Jones L, Thompson A, Vinnicombe S: Can shear-wave elastography predict response to neoadjuvant chemotherapy in women with invasive breast cancer? *Br J Cancer* 2013, 109(11):2798-2802.
52. Jones RL, Lakhani SR, Ring AE, Ashley S, Walsh G, Smith IE: Pathological complete response and residual DCIS following neoadjuvant chemotherapy for breast carcinoma. *Br J Cancer* 2006, 94(3):358-362.
53. Naoura I, Mazouni C, Ghanimeh J, Leymarie N, Garbay JR, Karsenti G, Sarfati B, Leduey A, Kolb F, Delaloge S *et al*: Factors influencing the decision to offer immediate breast reconstruction after mastectomy for ductal carcinoma in situ (DCIS): the Institut Gustave Roussy Breast Cancer Study Group experience. *Breast* 2013, 22(5):673-675.
54. Mazouni C, Peintinger F, Wan-Kau S, Andre F, Gonzalez-Angulo AM, Symmans WF, Meric- Bernstam F, Valero V, Hortobagyi GN, Pusztai L: Residual ductal carcinoma in situ in patients with complete eradication of invasive breast cancer after neoadjuvant chemotherapy does not adversely affect patient outcome. *J Clin Oncol* 2007, 25(19):2650-2655.
55. von Minckwitz G, Darb-Esfahani S, Loibl S, Huober J, Tesch H, Solbach C, Holms F, Eidtmann H, Dietrich K, Just M *et al*: Responsiveness of adjacent ductal carcinoma in situ and changes in HER2 status after neoadjuvant chemotherapy/trastuzumab treatment in early breast cancer--results from the GeparQuattro study (GBG 40). *Breast Cancer Res Treat* 2012, 132(3):863-870.
56. Segel MC, Paulus DD, Hortobagyi GN: Advanced primary breast cancer: assessment at mammography of response to induction chemotherapy. *Radiology* 1988, 169(1):49-54.
57. Ferranti C, Bergonzi S, Viganotti G, Piragine G, Barberini M, Bonadonna G, Coopmans De Yoldi G: [Microcalcifications in the diagnosis and follow-up after the primary chemotherapy of breast neoplasms]. *Radiol Med* 1992, 84(1-2):26-31.
58. Moskovic EC, Mansi JL, King DM, Murch CR, Smith IE: Mammography in the assessment of response to medical treatment of large primary breast cancer. *Clin Radiol* 1993, 47(5):339-344.
59. Vinnicombe SJ, MacVicar AD, Guy RL, Sloane JP, Powles TJ, Knee G, Husband JE:

Primary breast cancer: mammographic changes after neoadjuvant chemotherapy, with pathologic correlation. *Radiology* 1996, 198(2):333-340.
60. Junkermann H, von Fournier D: [Imaging methods for evaluating the response of breast carcinoma to preoperative chemotherapy]. *Radiologe* 1997, 37(9):726-732.
61. Adwani A, Lowe S, Ebbs SR: Disappearing microcalcification after neoadjuvant chemotherapy--a case report. *Eur J Surg Oncol* 2000, 26(1):98-100.
62. Matsuo K, Fukutomi T, Watanabe T, Hasegawa T, Tsuda H, Akashi-Tanaka S: Concordance in pathological response to neoadjuvant chemotherapy between invasive and noninvasive components of primary breast carcinomas. *Breast Cancer* 2002, 9(1):75-81.
63. Wu W, Kamma H, Ueno E, Fujiwara M, Satoh H, Hara H, Yashiro T, Aiyoshi Y: The intraductal component of breast cancer is poorly responsive to neo-adjuvant chemotherapy. *Oncol Rep* 2002, 9(5):1027-1031.
64. Fadul D, Rapelyea J, Schwartz AM, Brem RF: Development of malignant breast microcalcifications after neoadjuvant chemotherapy in advanced breast cancer. *Breast J* 2004, 10(2):141-145.
65. Noguera JJ, de Luis E, Alonso-Burgos A, Viteri S, Zornoza G, Pina L: [Mammographic findings in microcalcifications associated with breast cancer after neoadjuvant chemotherapy]. *Radiologia* 2007, 49(1):37-41.
66. Adrada BE, Huo L, Lane DL, Arribas EM, Resetkova E, Yang W: Histopathologic correlation of residual mammographic microcalcifications after neoadjuvant chemotherapy for locally advanced breast cancer. *Ann Surg Oncol* 2015, 22(4):1111-1117.
67. Libshitz HI, Montague ED, Paulus DD: Calcifications and the therapeutically irradiated breast. *AJR Am J Roentgenol* 1977, 128(6):1021-1025.
68. Kuerer HM, Buzdar AU, Mittendorf EA, Esteva FJ, Lucci A, Vence LM, Radvanyi L, Meric-Bernstam F, Hunt KK, Symmans WF: Biologic and immunologic effects of preoperative trastuzumab for ductal carcinoma in situ of the breast. *Cancer* 2011, 117(1):39-47.
69. Dialani V, Chadashvili T, Slanetz PJ: Role of imaging in neoadjuvant therapy for breast cancer. *Ann Surg Oncol* 2015, 22(5):1416-1424.
70. Marinovich ML, Houssami N, Macaskill P, Sardanelli F, Irwig L, Mamounas EP, von Minckwitz G, Brennan ME, Ciatto S: Meta-analysis of magnetic resonance imaging in detecting residual breast cancer after neoadjuvant therapy. *J Natl Cancer Inst* 2013, 105(5):321-333.
71. Berg WA, Madsen KS, Schilling K, Tartar M, Pisano ED, Larsen LH, Narayanan D, Ozonoff A, Miller JP, Kalinyak JE: Breast cancer: comparative effectiveness of positron emission mammography and MR imaging in presurgical planning for the ipsilateral breast. *Radiology* 2011, 258(1):59-72.
72. Morrow M, Waters J, Morris E: MRI for breast cancer screening, diagnosis, and treatment. *Lancet* 2011, 378(9805):1804-1811.
73. Turnbull LW: Dynamic contrast-enhanced MRI in the diagnosis and management of breast cancer.*NMR Biomed* 2009, 22(1):28-39.
74. Houssami N, Macaskill P, von Minckwitz G, Marinovich ML, Mamounas E: Meta-analysis of the association of breast cancer subtype and pathologic complete response to neoadjuvant chemotherapy. *Eur J Cancer* 2012, 48(18):3342-3354.
75. Chen JH, Feig B, Agrawal G, Yu H, Carpenter PM, Mehta RS, Nalcioglu O, Su MY: MRI evaluation of pathologically complete response and residual tumors in breast cancer after neoadjuvant chemotherapy. *Cancer* 2008, 112(1):17-26.
76. Hayes C, Padhani AR, Leach MO: Assessing changes in tumour vascular function using dynamic contrast-enhanced magnetic resonance imaging. *NMR Biomed* 2002, 15(2):154-163.
77. Loo CE, Straver ME, Rodenhuis S, Muller SH, Wesseling J, Vrancken Peeters MJ, Gilhuijs KG: Magnetic resonance imaging response monitoring of breast cancer during neoadjuvant

chemotherapy: relevance of breast cancer subtype. *J Clin Oncol* 2011, 29(6):660-666.
78. McGuire KP, Toro-Burguete J, Dang H, Young J, Soran A, Zuley M, Bhargava R, Bonaventura M, Johnson R, Ahrendt G: MRI staging after neoadjuvant chemotherapy for breast cancer: does tumor biology affect accuracy? *Ann Surg Oncol* 2011, 18(11):3149-3154.
79. De Los Santos JF, Cantor A, Amos KD, Forero A, Golshan M, Horton JK, Hudis CA, Hylton NM, McGuire K, Meric-Bernstam F *et al*: Magnetic resonance imaging as a predictor of pathologic response in patients treated with neoadjuvant systemic treatment for operable breast cancer. Translational Breast Cancer Research Consortium trial 017. *Cancer* 2013, 19(10):1776-1783.
80. Lobbes MB, Prevos R, Smidt M, Tjan-Heijnen VC, van Goethem M, Schipper R, Beets-Tan RG, Wildberger JE: The role of magnetic resonance imaging in assessing residual disease and pathologic complete response in breast cancer patients receiving neoadjuvant chemotherapy: a systematic review. *Insights Imaging* 2013, 4(2):163-175.
81. Mghanga FP, Lan X, Bakari KH, Li C, Zhang Y: Fluorine-18 fluorodeoxyglucose positron emission tomography-computed tomography in monitoring the response of breast cancer to neoadjuvant chemotherapy: a meta-analysis. *Clin Breast Cancer* 2013, 13(4):271-279.
82. Wahl RL, Zasadny K, Helvie M, Hutchins GD, Weber B, Cody R: Metabolic monitoring of breast cancer chemohormonotherapy using positron emission tomography: initial evaluation. *J Clin Oncol* 1993, 11(11):2101-2111.
83. Wang Y, Zhang C, Liu J, Huang G: Is 18F-FDG PET accurate to predict neoadjuvant therapy response in breast cancer? A meta-analysis. *Breast Cancer Res Treat* 2012, 31(2):357-369.
84. Kumar R, Chauhan A, Zhuang H, Chandra P, Schnall M, Alavi A: Clinicopathologic factors associated with false negative FDG-PET in primary breast cancer. *Breast Cancer Res Treat* 2006, 98(3):267- 274.
85. Groheux D, Espie M, Giacchetti S, Hindie E: Performance of FDG PET/CT in the clinical management of breast cancer. *Radiology* 2013, 266(2):388-405.
86. Berriolo-Riedinger A, Touzery C, Riedinger JM, Toubeau M, Coudert B, Arnould L, Boichot C, Cochet A, Fumoleau P, Brunotte F: [18F]FDG-PET predicts complete pathological response of breast cancer to neoadjuvant chemotherapy. *Eur J Nucl Med Mol Imaging* 2007, 34(12):1915-1924.
87. Pengel KE, Koolen BB, Loo CE, Vogel WV, Wesseling J, Lips EH, Rutgers EJ, Valdes Olmos RA, Vrancken Peeters MJ, Rodenhuis S *et al*: Combined use of (1)(8)F-FDG PET/CT and MRI for response monitoring of breast cancer during neoadjuvant chemotherapy. *Eur J Nucl Med Mol Imaging* 2014, 41(8):1515-1524.
88. Koolen BB, Pengel KE, Wesseling J, Vogel WV, Vrancken Peeters MJ, Vincent AD, Gilhuijs KG, Rodenhuis S, Rutgers EJ, Valdes Olmos RA: FDG PET/CT during neoadjuvant chemotherapy may predict response in ER-positive/HER2-negative and triple negative, but not in HER2-positive breast cancer. *Breast* 2013, 22(5):691- 697.
89. Koolen BB, Pengel KE, Wesseling J, Vogel WV, Vrancken Peeters MJ, Vincent AD, Gilhuijs KG, Rodenhuis S, Rutgers EJ, Valdes Olmos RA: Sequential (18)F-FDG PET/CT for early prediction of complete pathological response in breast and axilla during neoadjuvant chemotherapy. *Eur J Nucl Med Mol Imaging* 2014, 41(1):32-40.
90. Choi HK, Cho N, Moon WK, Im SA, Han W, Noh DY: Magnetic resonance imaging evaluation of residual ductal carcinoma in situ following preoperative chemotherapy in breast cancer patients. *Eur J Radiol* 2012, 81(4):737-743.
91. Park JS, Moon WK, Lyou CY, Cho N, Kang KW, Chung JK: The assessment of breast cancer response to neoadjuvant chemotherapy: comparison of magnetic resonance imaging and 18F- fluorodeoxyglucose positron emission tomography. *Acta Radiol* 2011, 52(1):21-28.

92. Heil J, Kummel S, Schaefgen B, Paepke S, Thomssen C, Rauch G, Ataseven B, Grosse R, Dreesmann V, Kuhn T *et al*: Diagnosis of pathological complete response to neoadjuvant chemotherapy in breast cancer by minimal invasive biopsy techniques. *Br J Cancer* 2015, 113(11):1565-1570.
93. Heil J: Diagnosis of pathological complete response by vacuum-assisted minimal invasive biopsy after neoadjuvant chemotherapy in breast cancer - Results from a prospective pilot stud. *Proceedings of the 2015 San Antonio Breast Cancer Symposium* 2015(P5-02-01).
94. Swisher AK, Abraham J, Bonner D, Gilleland D, Hobbs G, Kurian S, Yanosik MA, Vona-Davis L: Exercise and dietary advice intervention for survivors of triple-negative breast cancer: effects on body fat, physical function, quality of life, and adipokine profile. *Supportive care in cancer : official journal of the Multinational Association of Supportive Care in Cancer* 2015, 23(10):2995-3003.
95. Mainiero MB: Regional lymph node staging in breast cancer: the increasing role of imaging and ultrasound-guided axillary lymph node fine needle aspiration. *Radiol Clin North Am* 2010, 48(5):989- 997.
96. Alvarez S, Anorbe E, Alcorta P, Lopez F, Alonso I, Cortes J: Role of sonography in the diagnosis of axillary lymph node metastases in breast cancer: a systematic review. *AJR Am J Roentgenol* 2006, 186(5):1342-1348.
97. Kuerer HM, Newman LA, Fornage BD, Dhingra K, Hunt KK, Buzdar AU, Ames FC, Ross MI, Feig BW, Hortobagyi GN *et al*: Role of axillary lymph node dissection after tumor downstaging with induction chemotherapy for locally advanced breast cancer. *Ann Surg Oncol* 1998, 5(8):673-680.
98. Klauber-Demore N, Kuzmiak C, Rager EL, Ogunrinde OB, Ollila DW, Calvo BF, Kim HJ, Meyer A, Dees C, Graham M, 2nd *et al*: High-resolution axillary ultrasound is a poor prognostic test for determining pathologic lymph node status in patients undergoing neoadjuvant chemotherapy for locally advanced breast cancer. *Am J Surg* 2004, 188(4):386-389.
99. Vlastos G, Fornage BD, Mirza NQ, Bedi D, Lenert JT, Winchester DJ, Tolley SM, Ames FC, Ross MI, Feig BW *et al*: The correlation of axillary ultrasonography with histologic breast cancer downstaging after induction chemotherapy. *Am J Surg* 2000, 179(6):446-452.
100. Boughey JC, Ballman KV, Hunt KK, McCall LM, Mittendorf EA, Ahrendt GM, Wilke LG, Le-Petross HT: Axillary Ultrasound After Neoadjuvant Chemotherapy and Its Impact on Sentinel Lymph Node Surgery: Results From the American College of Surgeons Oncology Group Z1071 Trial (Alliance). *J Clin Oncol* 2015.
101. Bazan JG, White J: Imaging of the axilla before preoperative chemotherapy: implications for postmastectomy radiation. *Cancer* 2015, 121(8):1187-1194.
102. Hieken TJ, Boughey JC, Jones KN, Shah SS, Glazebrook KN: Imaging response and residual metastatic axillary lymph node disease after neoadjuvant chemotherapy for primary breast cancer. *Ann Surg Oncol* 2013, 20(10):3199-3204.
103. Javid S, Segara D, Lotfi P, Raza S, Golshan M: Can breast MRI predict axillary lymph node metastasis in women undergoing neoadjuvant chemotherapy. *Ann Surg Oncol* 2010, 17(7):1841-1846.
104. Koolen BB, Valdes Olmos RA, Elkhuizen PH, Vogel WV, Vrancken Peeters MJ, Rodenhuis S, Rutgers EJ: Locoregional lymph node involvement on 18F-FDG PET/CT in breast cancer patients scheduled for neoadjuvant chemotherapy. *Breast Cancer Res Treat* 2012, 135(1):231-240.
105. Pritchard KI, Julian JA, Holloway CM, McCready D, Gulenchyn KY, George R, Hodgson N, Lovrics P, Perera F, Elavathil L *et al*: Prospective study of 2- [(1)(8)F]fluorodeoxyglucose positron emission tomography in the assessment of regional nodal spread of disease in patients with breast cancer: an Ontario clinical oncology group study. *J Clin Oncol* 2012, 30(12):1274-

1279.
106. Koolen BB, Valdes Olmos RA, Wesseling J, Vogel WV, Vincent AD, Gilhuijs KG, Rodenhuis S, Rutgers EJ, Vrancken Peeters MJ: Early assessment of axillary response with (1)(8)F-FDG PET/CT during neoadjuvant chemotherapy in stage II-III breast cancer: implications for surgical management of the axilla. *Ann Surg Oncol* 2013, 20(7):2227-2235.
107. Straver ME, Aukema TS, Olmos RA, Rutgers EJ, Gilhuijs KG, Schot ME, Vogel WV, Peeters MJ: Feasibility of FDG PET/CT to monitor the response of axillary lymph node metastases to neoadjuvant chemotherapy in breast cancer patients. *Eur J Nucl Med Mol Imaging* 2010, 37(6):1069-1076.
108. Rousseau C, Devillers A, Campone M, Campion L, Ferrer L, Sagan C, Ricaud M, Bridji B, Kraeber- Bodere F: FDG PET evaluation of early axillary lymph node response to neoadjuvant chemotherapy in stage II and III breast cancer patients. *Eur J Nucl Med Mol Imaging* 2011, 38(6):1029-1036.
109. Jung SY, Kim SK, Nam BH, Min SY, Lee SJ, Park C, Kwon Y, Kim EA, Ko KL, Park IH *et al*: Prognostic Impact of [18F] FDG-PET in operable breast cancer treated with neoadjuvant chemotherapy. *Ann Surg Oncol* 2010, 17(1):247-253.
110. Kuerer HM, Newman LA, Smith TL, Ames FC, Hunt KK, Dhingra K, Theriault RL, Singh G, Binkley SM, Sneige N *et al*: Clinical course of breast cancer patients with complete pathologic primary tumor and axillary lymph node response to doxorubicin- based neoadjuvant chemotherapy. *J Clin Oncol* 1999, 17(2):460-469.
111. Kuerer HM, Sahin AA, Hunt KK, Newman LA, Breslin TM, Ames FC, Ross MI, Buzdar AU, Hortobagyi GN, Singletary SE: Incidence and impact of documented eradication of breast cancer axillary lymph node metastases before surgery in patients treated with neoadjuvant chemotherapy. *Ann Surg* 1999, 230(1):72-78.
112. Geyer CE, Jr., Tang G, Mamounas EP, et al. 21-Gene assay as predictor of chemotherapy benefit in HER2-negative breast cancer. *NPJ Breast Cancer.* 2018;4:37.
113. Sparano JA, Gray RJ, Makower DF, et al. Adjuvant Chemotherapy Guided by a 21-Gene Expression Assay in Breast Cancer. *N Engl J Med.* 2018;379(2):111-121.
114. Hortobagyi GN SS, Sledge GW, Winer EP, et al. Breast Cancer-specific Mortality in Patients with Node-negative and Node-positive Breast Cancer Guided by the 21-gene Assay: A SEER-Genomic Population-based Study. *San Antonio Breast Cancer Symposium*. 2018.
115. Pantel K, Alix-Panabieres C, Riethdorf S: Cancer micrometastases. *Nat Rev Clin Oncol* 2009, 6(6):339-351.
116. Lucci A, Hall CS, Lodhi AK, Bhattacharyya A, Anderson AE, Xiao L, Bedrosian I, Kuerer HM, Krishnamurthy S: Circulating tumour cells in non-metastatic breast cancer: a prospective study. *The lancet oncology* 2012, 13(7):688-695.
117. Hall C, Karhade, M., Bowman Bauldry, J., Valad, L., Kuerer, H., DeSynder, S., and Lucci, A.: Prognostic Value of Circulating Tumor Cells Identified prior to Surgical Resection in Non-Metastatic Breast Cancer Patients. *Journal of the American College of Surgeons* 2016, accepted for publication.
118. Janni W, Rack B, Terstappen LW, Pierga JY, Taran FA, Fehm T, Hall C, de Groot M, Bidard FC, Friedl TW *et al*: Pooled Analysis of the Prognostic Relevance of Circulating Tumor Cells in Primary Breast Cancer. *Clin Cancer Res* 2016.
119. Krishnamurthy S, Bischoff F, Ann Mayer J, Wong K, Pham T, Kuerer H, Lodhi A, Bhattacharyya A, Hall C, Lucci A: Discordance in HER2 gene amplification in circulating and disseminated tumor cells in patients with operable breast cancer. *Cancer Med* 2013, 2(2):226-233.
120. Schwaederle M, Husain H, Fanta PT, Piccioni DE, Kesari S, Schwab RB, Banks KC, Lanman RB, Talasaz A, Parker BA *et al*: Detection rate of actionable mutations diverse cancers

using a biopsy- free (blood) circulating tumor cell DNA assay. *Oncotarget* 2016.
121. Scott JG, Berglund A, Schell MJ, et al. A genome-based model for adjusting radiotherapy dose (GARD): a retrospective, cohort-based study. *Lancet Oncol.* 2017;18(2):202-211.
122. Sjostrom M, Chang SL, Fishbane N, Davicioni E, Zhao SG, Hartman L, Holmberg E, Feng FY, Speers CW, Pierce LJ, Malmstrom P, Ferno M, Karlsson P: Clinicogenomic Radiotherapy Classifier Predicting the Need for Intensified Locoregional Treatment After Breast-Conserving Surgery for Early-Stage Breast Cancer. J Clin Oncolo 2019, 37(35): 3340-3349.
123. Speers C, Chang SL, Pesch A, Ritter C, Olsen E, Chandler B, Moubadder L, Liu M, Cameron M, Michmerhuizen A, Wilder-Romans K, Zhao SG, Nyati S, Bartelink H, Feng FY, Pierce LJ: A Signature That May Be Predictive of Early Versus Late Recurrence After Radiation Treatment for Breast Cancer That May Inform the Biology of Early, Aggressive Recurrences. Int J Radiat Oncol Biol Phys 2020, 108(3): 686-696.
124. Arvold N, Taghian AG, Niemierko A, Abi Raad RF, Sreedhara M, Nguyen PL, Bellon JR, Wong JS, Smith BL, Harris JR: Age, breast cancer subtype approximation, and local recurrence after breast-conserving therapy. J Clin Oncol 2011, 29(29): 3885-3891.
125. Bellon JR, Guo H, Barry WT, Dang CT, Yardley DA, Moy B, Marcom PK, Albain KS, Rugo HS, Ellis M, Wolff AC, Carey LA, Overmoyer BA, Partridge AH, Hudis CA, Krop I, Burstein HJ, Winer EP, Tolaney SM: Local-regional recurrence in women with small node-negative, HER2-positive breast cancer: results from a prospective multi-institutional study (the APT trial). Breast Cancer Res Treat 2019, 176(2):303-310.
126. Swisher SK, Vila J, Tucker SL, Bedrosian I, Shaitelman SF, Litton JK, Smith BD, Caudle AS, Kuerer HM, Mittendorf EA: Locoregional Control According to Breast Cancer Subtype and Response to Neoadjuvant Chemotherapy in Breast Cancer Patients Undergoing Breast-conserving Therapy. *Ann Surg Oncol* 2016, 23(3):749- 756.
127. Thall PF, Simon RM, Estey EH: New statistical strategy for monitoring safety and efficacy in single- arm clinical trials. *J Clin Oncol* 1996, 14(1):296-303.
128. Woolson RF CW: Statistical Methods for the Analysis of Biomedical Data, 2nd Edition. . New York: John Wiley & Sons; 2002.
129. Kaplan EL MP: Nonparametric estimator from incomplete observations. *J American Statistical Association* 1958, 53:457-481.
130. Mantel N: Evaluation of survival data and two new rank order statistics arising in its consideration. *Cancer chemotherapy reports Part 1* 1966, 50(3):163-170.
131. DR C: Regression models and life tables (with discussion). *JR Statistical Soc* 1972, B(34):187-220.
132. Lee JJ TZ: A Versatile One-dimensional Distribution Plot: The BLip Plot. *The American Statistician* 1997, 51:353-358.
133. McCulloch CE SS: Generalized, Linear, and Mixed Models. New York: Wiley; 2001.
134. Linag KY ZS: Longitudinal data analysis using generalized linear models. *Biometrika* 1986, 73:13-22.

14.0 Attachments

Prioritization List (MD Anderson only)
Protocol Checklist (MD Anderson only)
FACT B-4 Patient Survey
BCTOS Patient Survey
DRS – Patient Survey
Data Quality Management Plan (for Participating Institutions outside MD Anderson)
Multicenter Serious Adverse Event Report form (for use by participating Institutions outside MD Anderson)
