# Supplementary material for: Patient-Reported Outcomes of Omission of Breast Surgery Following Neoadjuvant Systemic Therapy: A Nonrandomized Clinical Trial
Source: JAMA Netw Open. 2023 Sep 14;6(9):e2333933. doi: 10.1001/jamanetworkopen.2023.33933 (PMC10502524; doi:10.1001/jamanetworkopen.2023.33933)
Supplement: Supplement 2. — eTable 1. Associations Between Decision Regret Scale (DRS) Score and Patient Factors, Bivariate Analyses (Time Indicator Is Included in All Models) eTable 2. Associations Between Breast Cancer Treatment Outcomes Scale (BCTOS) Score and Patient Factors, Bivariate Analyses (Time Indicator Is Included in All Models) eTable 3. Associations Between Breast Cancer Treatment Outcomes Scale (BCTOS) Cosmetic Subscore and Patient Factors, Bivariate Analyses (Time Indicator Is Included in All Models) eTable 4. Associations Between Breast Cancer Treatment Outcomes Scale (BCTOS) Pain Subscore and Patient Factors, Bivariate Analyses (Time Indicator Is Included in All Models) eTable 5. Associations Between Breast Cancer Treatment Outcomes Scale (BCTOS) Edema Subscore and Patient Factors, Bivariate Analyses (Time Indicator Is Included in All Models) eTable 6. Associations Between Breast Cancer Treatment Outcomes Scale (BCTOS) Functional Subscore and Patient Factors, Bivariate Analyses (Time Indicator Is Included in All Models) eTable 7. Associations Between Functional Assessment of Cancer Therapy—Lymphedema (FACT-B+4) FACT-B Composite Score and Patient Factors, Bivariate Analyses (Time Indicator Is Included in All Models) eTable 8. Associations Between Functional Assessment of Cancer Therapy—Lymphedema (FACT-B+4) Physical Well-Being Subscore and Patient Factors, Bivariate Analyses (Time Indicator Is Included in All Models) eTable 9. Associations Between Functional Assessment of Cancer Therapy—Lymphedema (FACT-B+4) Physical Well-Being Subscore and Patient Factors, Multivariable Analyses eTable 10. Associations Between Functional Assessment of Cancer Therapy—Lymphedema (FACT-B+4) Social/Family Well-Being Subscore and Patient Factors, Bivariate Analyses (Time Indicator Is Included in All Models) eTable 11. Associations Between Functional Assessment of Cancer Therapy—Lymphedema (FACT-B+4) Social/Family Well-Being Subscore and Patient Factors, Multivariable Analysis eTable 12. Associatio [file jamanetwopen-e2333933-s002.pdf]

Supplemental Online Content

Johnson HM, Lin H, Shen Y, et al; and the Exceptional Responders Study Group. Patient-reported outcomes of omission of breast surgery following neoadjuvant systemic therapy. *JAMA Netw Open*. 2023;6(9):e2333933. doi:10.1001/jamanetworkopen.2023.33933

eTable 1. Associations Between Decision Regret Scale (DRS) Score and Patient Factors, Bivariate Analyses (Time Indicator Is Included in All Models)

eTable 2. Associations Between Breast Cancer Treatment Outcomes Scale (BCTOS) Score and Patient Factors, Bivariate Analyses (Time Indicator Is Included in All Models)

eTable 3. Associations Between Breast Cancer Treatment Outcomes Scale (BCTOS) Cosmetic Subscore and Patient Factors, Bivariate Analyses (Time Indicator Is Included in All Models)

eTable 4. Associations Between Breast Cancer Treatment Outcomes Scale (BCTOS) Pain Subscore and Patient Factors, Bivariate Analyses (Time Indicator Is Included in All Models)

eTable 5. Associations Between Breast Cancer Treatment Outcomes Scale (BCTOS) Edema Subscore and Patient Factors, Bivariate Analyses (Time Indicator Is Included in All Models)

eTable 6. Associations Between Breast Cancer Treatment Outcomes Scale (BCTOS) Functional Subscore and Patient Factors, Bivariate Analyses (Time Indicator Is Included in All Models)

eTable 7. Associations Between Functional Assessment of Cancer Therapy – Breast Cancer Plus Lymphedema (FACT-B+4) FACT-B Composite Score and Patient Factors, Bivariate Analyses (Time Indicator Is Included in All Models)

eTable 8. Associations Between Functional Assessment of Cancer Therapy – Breast Cancer Plus Lymphedema (FACT-B+4) Physical Well-Being Subscore and Patient Factors, Bivariate Analyses (Time Indicator Is Included in All Models)

eTable 9. Associations Between Functional Assessment of Cancer Therapy – Breast Cancer Plus Lymphedema (FACT-B+4) Physical Well-Being Subscore and Patient Factors, Multivariable Analyses

eTable 10. Associations Between Functional Assessment of Cancer Therapy – Breast Cancer Plus Lymphedema (FACT-B+4) Social/Family Well-Being Subscore and Patient Factors, Bivariate Analyses (Time Indicator Is Included in All Models)

eTable 11. Associations Between Functional Assessment of Cancer Therapy – Breast Cancer Plus Lymphedema (FACT-B+4) Social/Family Well-Being Subscore and Patient Factors, Multivariable Analysis

eTable 12. Associations Between Functional Assessment of Cancer Therapy – Breast Cancer Plus Lymphedema (FACT-B+4) Emotional Well-Being Subscore and Patient Factors, Bivariate Analyses (Time Indicator Is Included in All Models)

© 2023 Johnson HM et al. *JAMA Network Open.*

eTable 13. Associations Between Functional Assessment of Cancer Therapy – Breast Cancer Plus Lymphedema (FACT-B+4) Emotional Well-Being Subscore and Patient Factors, Multivariable Analysis

eTable 14. Associations Between Functional Assessment of Cancer Therapy – Breast Cancer Plus Lymphedema (FACT-B+4) Functional Well-Being Subscore and Patient Factors, Bivariate Analyses (Time Indicator Is Included in All Models)

eTable 15. Associations Between Functional Assessment of Cancer Therapy – Breast Cancer Plus Lymphedema (FACT-B+4) Functional Well-Being Subscore and Patient Factors, Multivariable Analysis

eTable 16. Associations Between Functional Assessment of Cancer Therapy – Breast Cancer Plus Lymphedema (FACT-B+4) Breast Cancer Subscore and Patient Factors, Bivariate Analyses (Time Indicator Is Included in All Models)

eTable 17. Associations Between Functional Assessment of Cancer Therapy – Breast Cancer Plus Lymphedema (FACT-B+4) Breast Cancer Subscore and Patient Factors, Multivariable Analysis

eTable 18. Associations Between Functional Assessment of Cancer Therapy – Breast Cancer Plus Lymphedema (FACT-B+4) ARM Subscore and Patient Factors, Bivariate Analyses (Time Indicator Is Included in All Models)

This supplemental material has been provided by the authors to give readers additional information about their work.

© 2023 Johnson HM et al. *JAMA Network Open.*

Abbreviations:
SE = standard error, CL = confidence limit, Ref = reference, HR = hormone receptor, HER2 = human epidermal growth factor receptor 2

eTable 1. Associations Between Decision Regret Scale (DRS) Score and Patient Factors, Bivariate Analyses (Time Indicator Is Included in All Models)

Variable		Estimate	SE	LowerCL	UpperCL	p-Value for comparison with reference group	p-Value for overall effect
Age	Per year increase	-0.3428	0.1808	-0.6972	0.0116	0.0580	0.05797
Axillary Surgery receipt	Yes	4.6827	2.5560	-0.3269	9.6923	0.0669	0.06694
	No	Ref	Ref	Ref	Ref		
Biopsy during surveillance	Yes	3.5879	6.9860	-10.1043	17.2802	0.6075	0.60754
	No	Ref	Ref	Ref	Ref		
Cancer Subtype	Triple negative	0.4631	4.4836	-8.3245	9.2507	0.9177	0.96076
	HER2+HR+	1.2006	4.8033	-8.2137	10.6148	0.8026	
	HER2+HR-	Ref	Ref	Ref	Ref		
Endocrine therapy receipt	Yes	0.9158	3.2512	-5.4564	7.2880	0.7782	0.77818
	No	Ref	Ref	Ref	Ref		
Ethnicity	Non-Hispanic	1.9721	5.7016	-9.2028	13.1471	0.7294	0.72942
	Hispanic	Ref	Ref	Ref	Ref		
Race	White	7.1136	2.6389	1.9415	12.2858	0.0070	0.00702
	Black or Asian	Ref	Ref	Ref	Ref		
Timepoint	36 months	-2.5104	6.7014	-15.6450	10.6242	0.7080	0.00038
	12 months	-10.0052	2.6775	-15.2531	-4.7573	0.0002	
	6 months	-10.4839	2.7119	-15.7992	-5.1686	0.0001	
	Baseline	Ref	Ref	Ref	Ref		
Regional Nodal radiation receipt	Yes	2.1427	2.9044	-3.5497	7.8352	0.4607	0.46066
	No	Ref	Ref	Ref	Ref		

© 2023 Johnson HM et al. *JAMA Network Open.*

eTable 2. Associations Between Breast Cancer Treatment Outcomes Scale (BCTOS) Score and Patient Factors, Bivariate Analyses (Time Indicator Is Included in All Models)

Variable		Estimate	SE	LowerCL	UpperCL	p-Value for comparison with reference group	p-Value for overall effect
Age	Per year increase	-0.0054	0.0042	-0.0136	0.0028	0.1976	0.19759
Axillary Surgery receipt	Yes	0.0775	0.0722	-0.0640	0.2189	0.2832	0.28321
	No	Ref	Ref	Ref	Ref		
Biopsy during surveillance	Yes	0.0837	0.0579	-0.0297	0.1972	0.1479	0.14793
	No	Ref	Ref	Ref	Ref		
Cancer Subtype	Triple negative	0.0731	0.0692	-0.0624	0.2087	0.2903	0.42038
	HER2+HR+	0.1157	0.0944	-0.0692	0.3007	0.2201	
	HER2+HR-	Ref	Ref	Ref	Ref		
Endocrine therapy receipt	Yes	0.0697	0.0804	-0.0879	0.2273	0.3862	0.38616
	No	Ref	Ref	Ref	Ref		
Ethnicity	Non-Hispanic	0.0188	0.0837	-0.1452	0.1828	0.8224	0.82237
	Hispanic	Ref	Ref	Ref	Ref		
Race	White	-0.1395	0.0676	-0.2719	-0.0071	0.0390	0.03898
	Black or Asian	Ref	Ref	Ref	Ref		
Timepoint	Month36	0.1989	0.0651	0.0714	0.3265	0.0022	0.00003
	Month12	0.1823	0.0618	0.0612	0.3033	0.0032	
	Month6	0.2317	0.0503	0.1330	0.3303	<.0001	
	Baseline	Ref	Ref	Ref	Ref		
Regional Nodal radiation receipt	Yes	0.0648	0.0967	-0.1247	0.2543	0.5027	0.50274
	No	Ref	Ref	Ref	Ref		

© 2023 Johnson HM et al. *JAMA Network Open.*

eTable 3. Associations Between Breast Cancer Treatment Outcomes Scale (BCTOS) Cosmetic Subscore and Patient Factors, Bivariate Analyses (Time Indicator Is Included in All Models)

Variable		Estimate	SE	LowerCL	UpperCL	p-Value for comparison with reference group	p-Value for overall effect
Age	Per year increase	-0.0128	0.0055	-0.0236	-0.0019	0.0212	0.02123
Axillary Surgery receipt	Yes	0.0997	0.1094	-0.1146	0.3140	0.3619	0.36187
	No	Ref	Ref	Ref	Ref		
Biopsy during surveillance	Yes	0.1768	0.1567	-0.1302	0.4839	0.2591	0.25906
	No	Ref	Ref	Ref	Ref		
Cancer Subtype	Triple negative	0.0897	0.0915	-0.0896	0.2691	0.3266	0.55920
	HER2+HR+	0.1298	0.1533	-0.1706	0.4302	0.3970	
	HER2+HR-	Ref	Ref	Ref	Ref		
Endocrine therapy receipt	Yes	0.0737	0.1369	-0.1947	0.3421	0.5905	0.59051
	No	Ref	Ref	Ref	Ref		
Ethnicity	Non-Hispanic	0.0248	0.1732	-0.3147	0.3642	0.8864	0.88637
	Hispanic	Ref	Ref	Ref	Ref		
Race	White	-0.1074	0.0952	-0.2941	0.0792	0.2591	0.25913
	Other	Ref	Ref	Ref	Ref		
Timepoint	36 months	0.4535	0.1475	0.1644	0.7427	0.0021	0.00001
	12 months	0.2219	0.0839	0.0575	0.3863	0.0082	
	6 months	0.3145	0.0650	0.1871	0.4419	<.0001	
	Baseline	Ref	Ref	Ref	Ref		
Regional Nodal radiation receipt	No	0.0943	0.1376	-0.1754	0.3639	0.4933	0.49329
	Yes	Ref	Ref	Ref	Ref		

© 2023 Johnson HM et al. *JAMA Network Open.*

eTable 4. Associations Between Breast Cancer Treatment Outcomes Scale (BCTOS) Pain Subscore and Patient Factors, Bivariate Analyses (Time Indicator Is Included in All Models)

Variable		Estimate	SE	LowerCL	UpperCL	p-Value for comparison with reference group	p-Value for overall effect
Age	Per year increase	-0.0160	0.0116	-0.0389	0.0068	0.1688	0.16875
Axillary Surgery receipt	Yes	0.1133	0.1484	-0.1776	0.4041	0.4453	0.44526
	No	Ref	Ref	Ref	Ref		
Biopsy during surveillance	Yes	-0.0005	0.1294	-0.2542	0.2531	0.9968	0.99677
	No	Ref	Ref	Ref	Ref		
Cancer Subtype	Triple negative	0.3709	0.1522	0.0726	0.6693	0.0148	0.00086
	HER2+HR+	0.5468	0.1554	0.2421	0.8515	0.0004	
	HER2+HR-	Ref	Ref	Ref	Ref		
Endocrine therapy receipt	Yes	0.3111	0.1557	0.0059	0.6163	0.0457	0.04571
	No	Ref	Ref	Ref	Ref		
Ethnicity	Non-Hispanic	0.0038	0.2402	-0.4670	0.4746	0.9873	0.98728
	Hispanic	Ref	Ref	Ref	Ref		
Race	White	-0.2889	0.2393	-0.7580	0.1802	0.2274	0.22739
	Black or Asian	Ref	Ref	Ref	Ref		
Timepoint	36 months	0.1558	0.1289	-0.0970	0.4085	0.2270	0.01122
	12 months	0.3235	0.1416	0.0459	0.6012	0.0224	
	6 months	0.3441	0.1091	0.1302	0.5579	0.0016	
	Baseline	Ref	Ref	Ref	Ref		
Regional Nodal radiation receipt	No	-0.0086	0.1690	-0.3399	0.3226	0.9593	0.95927
	Yes	Ref	Ref	Ref	Ref		

© 2023 Johnson HM et al. *JAMA Network Open.*

eTable 5. Associations Between Breast Cancer Treatment Outcomes Scale (BCTOS) Edema Subscore and Patient Factors, Bivariate Analyses (Time Indicator Is Included in All Models)

Variable		Estimate	SE	LowerCL	UpperCL	p-Value for comparison with reference group	p-Value for overall effect
Age	Per year increase	-0.0035	0.0056	-0.0144	0.0073	0.5234	0.52338
Axillary Surgery receipt	Yes	0.0547	0.1017	-0.1446	0.2540	0.5908	0.59077
	No	Ref	Ref	Ref	Ref		
Biopsy during surveillance	Yes	0.1831	0.1016	-0.0159	0.3822	0.0714	0.07136
	No	Ref	Ref	Ref	Ref		
Cancer Subtype	Triple negative	0.0144	0.0574	-0.0982	0.1269	0.8025	0.52608
	HER2+HR+	0.1369	0.1210	-0.1002	0.3740	0.2579	
	HER2+HR-	Ref	Ref	Ref	Ref		
Endocrine therapy receipt	Yes	0.1280	0.1163	-0.0999	0.3560	0.2709	0.27095
	No	Ref	Ref	Ref	Ref		
Ethnicity	Non-Hispanic	-0.0159	0.0748	-0.1624	0.1307	0.8319	0.83192
	Hispanic	Ref	Ref	Ref	Ref		
Race	White	-0.1636	0.0820	-0.3243	-0.0030	0.0459	0.04592
	Black or Asian	Ref	Ref	Ref	Ref		
Timepoint	36 months	0.0104	0.0717	-0.1300	0.1509	0.8843	0.03635
	12 months	0.1113	0.0674	-0.0207	0.2434	0.0985	
	6 months	0.1452	0.0682	0.0115	0.2789	0.0333	
	Baseline	Ref	Ref	Ref	Ref		
Regional Nodal radiation receipt	No	0.0736	0.1369	-0.1947	0.3418	0.5909	0.59091
	Yes	Ref	Ref	Ref	Ref		

© 2023 Johnson HM et al. *JAMA Network Open.*

eTable 6. Associations Between Breast Cancer Treatment Outcomes Scale (BCTOS) Functional Subscore and Patient Factors, Bivariate Analyses (Time Indicator Is Included in All Models)

Variable		Estimate	SE	LowerCL	UpperCL	p-Value for comparison with reference group	p-Value for overall effect
Age	Per year increase	0.0053	0.0035	-0.0015	0.0121	0.1298	0.12980
Axillary Surgery receipt	Yes	0.0519	0.0802	-0.1053	0.2092	0.5174	0.51736
	No	Ref	Ref	Ref	Ref		
Biopsy during surveillance	Yes	-0.0647	0.1102	-0.2806	0.1513	0.5573	0.55729
	No	Ref	Ref	Ref	Ref		
Cancer Subtype	Triple negative	-0.0308	0.0928	-0.2126	0.1511	0.7401	0.51183
	HER2+HR+	-0.0796	0.0853	-0.2468	0.0876	0.3509	
	HER2+HR-	Ref	Ref	Ref	Ref		
Endocrine therapy receipt	Yes	-0.0603	0.0526	-0.1634	0.0428	0.2513	0.25132
	No	Ref	Ref	Ref	Ref		
Ethnicity	Non-Hispanic	0.0776	0.0683	-0.0562	0.2114	0.2557	0.25574
	Hispanic	Ref	Ref	Ref	Ref		
Race	White	-0.1094	0.0982	-0.3020	0.0832	0.2655	0.26546
	Black or Asian	Ref	Ref	Ref	Ref		
Timepoint	36 months	0.0691	0.0767	-0.0813	0.2195	0.3678	0.03640
	12 months	0.1208	0.0459	0.0308	0.2108	0.0086	
	6 months	0.1382	0.0551	0.0303	0.2462	0.0121	
	Baseline	Ref	Ref	Ref	Ref		
Regional Nodal Regional receipt	No	0.0634	0.1016	-0.1358	0.2626	0.5327	0.53271
	Yes	Ref	Ref	Ref	Ref		

© 2023 Johnson HM et al. *JAMA Network Open.*

eTable 7. Associations Between Functional Assessment of Cancer Therapy – Breast Cancer Plus Lymphedema (FACT-B+4) FACT-B Composite Score and Patient Factors, Bivariate Analyses (Time Indicator Is Included in All Models)

Variable		Estimate	Stderr	LowerCL	UpperCL	p-Valuer for comparison with reference group	p-Value for overall effect
Age	Per year increase	0.0071	0.2889	-0.5590	0.5733	0.9803	0.98034
Axillary Surgery receipt	Yes	0.6882	3.9693	-7.0915	8.4680	0.8623	0.86234
	No	Ref	Ref	Ref	Ref		
Biopsy during surveillance	Yes	-2.3815	6.7482	-15.6077	10.8447	0.7242	0.72416
	No	Ref	Ref	Ref	Ref		
Cancer Subtype	Triple negative	-1.7327	5.6330	-12.7731	9.3078	0.7584	0.32167
	HER2+HR+	5.1289	6.4595	-7.5314	17.7892	0.4272	
	HER2+HR-	Ref	Ref	Ref	Ref		
Endocrine Therapy receipt	Yes	6.2099	4.6115	-2.8284	15.2482	0.1781	0.17810
	No	Ref	Ref	Ref	Ref		
Ethnicity	Non-Hispanic	-10.1311	2.9564	-15.9255	-4.3368	0.0006	0.00061
	Hispanic	Ref	Ref	Ref	Ref		
Race	White	-4.5386	4.4776	-13.3146	4.2374	0.3108	0.31077
	Black or Asian	Ref	Ref	Ref	Ref		
Timepoint	36 months	5.1990	3.0902	-0.8577	11.2557	0.0925	0.03856
	12 months	5.1443	3.2682	-1.2612	11.5498	0.1155	
	6 months	6.0928	2.1968	1.7871	10.3985	0.0055	
	Baseline	Ref	Ref	Ref	Ref		
Regional Nodal Radiation receipt	Yes	-1.1489	4.4724	-9.9146	7.6168	0.7973	0.79727
	No	Ref	Ref	Ref	Ref		

© 2023 Johnson HM et al. *JAMA Network Open.*

eTable 8. Associations Between Functional Assessment of Cancer Therapy – Breast Cancer Plus Lymphedema (FACT-B+4) Physical Well-Being Subscore and Patient Factors, Bivariate Analyses (Time Indicator Is Included in All Models)

Variable		Estimate	SE	LowerCL	UpperCL	p-Value for comparison with reference group	p-Value for overall effect
Age	Per year increase	-0.0216	0.0751	-0.1688	0.1257	0.7741	0.77406
Axillary Surgery receipt	Yes	1.2275	1.0185	-0.7688	3.2238	0.2281	0.22815
	No	Ref	Ref	Ref	Ref		
Biopsy during surveillance	Yes	1.0986	1.1742	-1.2027	3.4000	0.3494	0.34945
	No	Ref	Ref	Ref	Ref		
Cancer Subtype	Triple negative	0.0816	1.1457	-2.1639	2.3272	0.9432	0.00493
	HER2+HR+	2.4296	0.9481	0.5714	4.2877	0.0104	
	HER2+HR-	Ref	Ref	Ref	Ref		
Endocrine Therapy receipt	Yes	2.3768	0.7403	0.9260	3.8277	0.0013	0.00132
	No	Ref	Ref	Ref	Ref		
Ethnicity	Non-Hispanic	-1.7679	0.7788	-3.2942	-0.2416	0.0232	0.02320
	Hispanic	Ref	Ref	Ref	Ref		
Race	White	0.0521	1.2385	-2.3753	2.4796	0.9664	0.96643
	Black or Asian	Ref	Ref	Ref	Ref		
Timepoint*	36 months	1.3445	0.8347	-0.2915	2.9804	0.1072	0.00058
	12 months	1.7319	0.7868	0.1898	3.2740	0.0277	0.00058
	6 months	1.9301	0.4765	0.9962	2.8641	<.0001	
	Baseline	Ref	Ref	Ref	Ref		
Regional Nodal Radiation receipt	Yes	0.1946	1.1217	-2.0039	2.3932	0.8622	0.86224
	No	Ref	Ref	Ref	Ref		

© 2023 Johnson HM et al. *JAMA Network Open.*

eTable 9. Associations Between Functional Assessment of Cancer Therapy – Breast Cancer Plus Lymphedema (FACT-B+4) Physical Well-Being Subscore and patient Factors, Multivariable Analyses

As cancer subtype and endocrine therapy were collinear, two models were built.

Model 1

Variable		Estimate	SE	LowerCL	UpperCL	p-Value for comparison with reference group	p-Value for overall effect
Cancer Subtype	Triple negative	0.0816	1.1457	-2.1639	2.3272	0.9432	.00493
	HER2+HR+	2.4296	0.9481	0.5714	4.2877	0.0104	
	HER2+HR-	Ref	Ref	Ref	Ref		
Timepoint	36 months	1.3052	0.8348	-0.3310	2.9415	0.1179	.00058
	12 months	1.7458	0.7891	0.1991	3.2924	0.0269	
	6 months	1.9301	0.4765	0.9962	2.8641	<.0001	
	Baseline	Ref	Ref	Ref	Ref		

Model 2

Variable		Estimate	SE	LowerCL	UpperCL	p-Value for comparison with reference group	p-Value for overall effect
Endocrine Therapy receipt	Yes	2.3768	0.7403	0.9260	3.8277	0.0013	.00132
	No	Ref	Ref	Ref	Ref		
Timepoint	36 months	1.3052	0.8326	-0.3267	2.9371	0.1170	.00059
	12 months	1.7445	0.7870	0.2020	3.2869	0.0267	
	6 months	1.9301	0.4765	0.9962	2.8641	<.0001	
	Baseline	Ref	Ref	Ref	Ref		

© 2023 Johnson HM et al. *JAMA Network Open.*

eTable 10. Associations Between Functional Assessment of Cancer Therapy – Breast Cancer Plus Lymphedema (FACT-B+4) Social/Family Well-Being Subscore and Patient Factors, Bivariate Analyses (Time Indicator Is Included in All Models)

Variable		Estimate	SE	LowerCL	UpperCL	p-Value for comparison with reference group	p-Value for overall effect
Age	Per year increase	-0.0367	0.0603	-0.1549	0.0815	0.5425	0.54253
Axillary Surgery receipt	Yes	0.2058	1.0280	-1.8091	2.2206	0.8413	0.84135
	No	Ref	Ref	Ref	Ref		
Biopsy during surveillance	Yes	-0.4367	1.2468	-2.8804	2.0070	0.7261	0.72613
	No	Ref	Ref	Ref	Ref		
Cancer Subtype	Triple negative	-0.2230	1.4357	-3.0369	2.5909	0.8766	0.30596
	HER2+HR+	1.2906	1.5772	-1.8006	4.3819	0.4132	
	HER2+HR-	Ref	Ref	Ref	Ref		
Endocrine therapy receipt	Yes	1.4295	1.0313	-0.5917	3.4507	0.1657	0.16569
	No	Ref	Ref	Ref	Ref		
Ethnicity	Non-Hispanic	-2.2977	0.7133	-3.6957	-0.8996	0.0013	0.00128
	Hispanic	Ref	Ref	Ref	Ref		
Race	White	0.3387	1.4717	-2.5457	3.2231	0.8180	0.81798
	Black or Asian	Ref	Ref	Ref	Ref		
Timepoint	36 months	-0.2562	0.6114	-1.4545	0.9421	0.6752	0.41488
	12 months	-1.4652	0.9081	-3.2450	0.3146	0.1066	
	6 months	-0.4903	0.5440	-1.5565	0.5758	0.3674	
	Baseline	Ref	Ref	Ref	Ref		
Regional nodal radiation receipt	Yes	1.0534	1.0838	-1.0709	3.1777	0.3311	0.33108
	No	Ref	Ref	Ref	Ref		

© 2023 Johnson HM et al. *JAMA Network Open.*

eTable 11. Associations Between Functional Assessment of Cancer Therapy – Breast Cancer Plus Lymphedema (FACT-B+4) Social/Family Well-Being Subscore and Patient Factors, Multivariable Analysis

Variable		Estimate	SE	LowerCL	UpperCL	p-Value for comparison with reference group	p-Value for overall effect
Ethnicity	Non-Hispanic	-2.2977	0.7133	-3.6957	-0.8996	0.0013	0.00128
	Hispanic	Ref	Ref	Ref	Ref		
Timepoint	36 months	-0.5190	0.5536	-1.6040	0.5660	0.3485	0.13307
	12 months	-1.7076	0.8785	-3.4295	0.0143	0.0519	
	6 months	-0.3345	0.5595	-1.4312	0.7622	0.5500	
	Baseline	Ref	Ref	Ref	Ref		

© 2023 Johnson HM et al. *JAMA Network Open.*

eTable 12. Associations Between Functional Assessment of Cancer Therapy – Breast Cancer Plus Lymphedema (FACT-B+4) Emotional Well-Being Subscore and Patient Factors, Bivariate Analyses (Time Indicator Is Included in All Models)

Variable		Estimate	SE	LowerCL	UpperCL	p-Value for comparison with reference group	p-Value for overall effect
Age	Per year increase	0.0643	0.0403	-0.0147	0.1434	0.1105	0.11049
Axillary Surgery receipt	Yes	-0.2699	0.7852	-1.8088	1.2690	0.7310	0.73101
	No	Ref	Ref	Ref	Ref		
Biopsy during surveillance	Yes	-1.4959	1.4190	-4.2771	1.2852	0.2918	0.29178
	No	Ref	Ref	Ref	Ref		
Cancer Subtype	Triple negative	0.3395	0.7620	-1.1539	1.8329	0.6559	0.59359
	HER2+HR+	1.1017	1.0921	-1.0388	3.2421	0.3131	
	HER2+HR-	Ref	Ref	Ref	Ref		
Endocrine therapy receipt	Yes	0.8891	0.9104	-0.8953	2.6735	0.3288	0.32879
	No	Ref	Ref	Ref	Ref		
Ethnicity	Non-Hispanic	-0.5577	1.0197	-2.5563	1.4409	0.5844	0.58444
	Hispanic	Ref	Ref	Ref	Ref		
Race	White	-2.0413	0.4587	-2.9403	-1.1423	<.0001	0.00001
	Black or Asian	Ref	Ref	Ref	Ref		
Timepoint	36 months	0.1428	0.6390	-1.1097	1.3952	0.8232	0.17845
	12 months	-0.5655	0.5698	-1.6824	0.5514	0.3210	
	6 months	0.4452	0.4303	-0.3981	1.2884	0.3008	
	Baseline	Ref	Ref	Ref	Ref		
Regional nodal radiation receipt	Yes	-0.9634	0.8887	-2.7052	0.7785	0.2784	0.27837
	No	Ref	Ref	Ref	Ref		

© 2023 Johnson HM et al. *JAMA Network Open.*

eTable 13. Associations Between Functional Assessment of Cancer Therapy – Breast Cancer Plus Lymphedema (FACT-B+4) Emotional Well-Being Subscore and Patient Factors, Multivariable Analysis

Variable		Estimate	SE	LowerCL	UpperCL	p-Value for comparison with reference group	p-Value for overall effect
Race	White	-2.0413	0.4587	-2.9403	-1.1423	<.0001	0.00001
	Black or Asian	Ref	Ref	Ref	Ref		
Timepoint	36 months	0.0689	0.6365	-1.1786	1.3163	0.9139	0.18576
	12 months	-0.5985	0.5733	-1.7221	0.5251	0.2965	.
	6 months	0.4452	0.4303	-0.3981	1.2884	0.3008	.
	Baseline	Ref	Ref	Ref	Ref		

© 2023 Johnson HM et al. *JAMA Network Open.*

eTable 14. Associations Between Functional Assessment of Cancer Therapy – Breast Cancer Plus Lymphedema (FACT-B+4) Functional Well-Being Subscore and Patient Factors, Bivariate Analyses (Time Indicator Is Included in All Models)

Variable		Estimate	SE	LowerCL	UpperCL	p-Value for comparison with reference group	p-Value for overall effect
Age	Per year	-0.0823	0.0946	-0.2677	0.1031	0.3845	0.38448
Axillary Surgery receipt	Yes	-0.5235	1.3341	-3.1383	2.0912	0.6947	0.69473
	No	Ref	Ref	Ref	Ref		
Biopsy during surveillance	Yes	-1.9009	2.4685	-6.7391	2.9372	0.4412	0.44124
	No	Ref	Ref	Ref	Ref		
Cancer Subtype	Triple negative	-1.7152	1.5977	-4.8466	1.4162	0.2830	0.29346
	HER2+HR+	0.5005	2.0452	-3.5080	4.5091	0.8067	.
	HER2+HR-	Ref	Ref	Ref	Ref		
Endocrine therapy receipt	Yes	1.5610	1.6543	-1.6814	4.8033	0.3454	0.34538
	No	Ref	Ref	Ref	Ref		
Ethnicity	Non-Hispanic	-3.8140	0.9919	-5.7581	-1.8698	0.0001	0.00012
	Hispanic	Ref	Ref	Ref	Ref		
Race	White	-1.6358	1.5845	-4.7414	1.4699	0.3019	0.30191
	Black or Asian	Ref	Ref	Ref	Ref		
Timepoint	36 months	2.2254	1.3124	-0.3469	4.7977	0.0900	0.16519.
	12 months	2.1988	1.2930	-0.3354	4.7330	0.0890	
	6 months	2.4839	1.1070	0.3141	4.6536	0.0249	
	Baseline	Ref	Ref	Ref	Ref		
Regional nodal radiation receipt	Yes	-1.4164	1.4419	-4.2425	1.4097	0.3259	0.32594
	No	Ref	Ref	Ref	Ref		

© 2023 Johnson HM et al. *JAMA Network Open.*

eTable 15. Associations Between Functional Assessment of Cancer Therapy – Breast Cancer Plus Lymphedema (FACT-B+4) Functional Well-Being Subscore and Patient Factors, Multivariable Analysis

Variable		Estimate	SE	LowerCL	UpperCL	p-Value for comparison with reference group	p-Value for overall effect
Ethnicity	Non-Hispanic	-3.8140	0.9919	-5.7581	-1.8698	0.0001	0.00012
	Hispanic	Ref	Ref	Ref	Ref		
Timepoint	36 months	2.0984	1.3789	-0.6042	4.8010	0.1281	0.21151
	12 months	2.0714	1.3605	-0.5951	4.7378	0.1279	
	6 months	2.4828	1.1771	0.1757	4.7898	0.0349	
	Baseline	Ref	Ref	Ref	Ref		

© 2023 Johnson HM et al. *JAMA Network Open.*

eTable 16. Associations Between Functional Assessment of Cancer Therapy – Breast Cancer Plus Lymphedema (FACT-B+4) Breast Cancer Subscore and Patient Factors, Bivariate Analyses (Time Indicator Is Included in All Models)

Variable		Estimate	SE	LowerCL	UpperCL	p-Value for comparison with reference group	p-Value for overall effect
Age	Per year increase	0.0666	0.0915	-0.1127	0.2460	0.4666	0.46661
Axillary Surgery receipt	Yes	-0.2915	1.3854	-3.0069	2.4239	0.8333	0.83335
	No	Ref	Ref	Ref	Ref		
Biopsy during surveillance	Yes	-1.7679	1.9221	-5.5352	1.9993	0.3577	0.35767
	No	Ref	Ref	Ref	Ref		
Cancer Subtype	Triple negative	-0.3475	1.5508	-3.3869	2.6919	0.8227	0.96568
	HER2+HR+	-0.4702	1.9387	-4.2701	3.3296	0.8084	
	HER2+HR-	Ref	Ref	Ref	Ref		
Endocrine therapy receipt	Yes	-0.2556	1.6031	-3.3977	2.8865	0.8733	0.87334
	No	Ref	Ref	Ref	Ref		
Ethnicity	Non-Hispanic	-1.7570	0.7810	-3.2877	-0.2263	0.0245	0.02446
	Hispanic	Ref	Ref	Ref	Ref		
Race	White	-1.3107	0.8448	-2.9665	0.3451	0.1208	0.12079
	Black or Asian	Ref	Ref	Ref	Ref		
Timepoint	36 months	1.8189	1.1530	-0.4410	4.0787	0.1147	0.03197
	12 months	3.2267	1.1336	1.0048	5.4486	0.0044	
	6 months	1.7240	0.9600	-0.1576	3.6057	0.0725	
	Baseline	Ref	Ref	Ref	Ref		
Regional Nodal Radiation receipt	Yes	-0.3135	1.7110	-3.6669	3.0399	0.8546	0.85461
	No	Ref	Ref	Ref	Ref		

© 2023 Johnson HM et al. *JAMA Network Open.*

eTable 17. Associations Between Functional Assessment of Cancer Therapy – Breast Cancer Plus Lymphedema (FACT-B+4) Breast Cancer Subscore and Patient Factors, Multivariable Analysis

Variable		Estimate	SE	LowerCL	UpperCL	p-Value for comparison with reference group	p-Value for overall effect
Ethnicity	Non-Hispanic	-1.7570	0.7810	-3.2877	-0.2263	0.0245	0.02446
	Hispanic	Ref	Ref	Ref	Ref		
Timepoint	36 months	1.9006	1.1966	-0.4446	4.2458	0.1122	0.04652
	12 months	3.2431	1.1910	0.9087	5.5775	0.0065	
	6 months	1.8084	1.0006	-0.1527	3.7696	0.0707	
	Baseline	Ref	Ref	Ref	Ref		

© 2023 Johnson HM et al. *JAMA Network Open.*

eTable 18. Associations Between Functional Assessment of Cancer Therapy – Breast Cancer Plus Lymphedema (FACT-B+4) ARM Subscore and Patient Factors, Bivariate Analyses (Time Indicator Is Included in All Models)

Variable		Estimate	SE	LowerCL	UpperCL	p-Value for comparison with reference group	p-Value for overall effect
Age	Per year increase	0.0036	0.0177	-0.0311	0.0384	0.8371	0.83709
Axillary Surgery receipt	Yes	-0.5360	0.4137	-1.3468	0.2749	0.1951	0.19513
	No	Ref	Ref	Ref	Ref		
Biopsy during surveillance	Yes	0.1858	0.4776	-0.7503	1.1219	0.6972	0.69721
	No	Ref	Ref	Ref	Ref		
Cancer Subtype	Triple negative	0.0031	0.4296	-0.8389	0.8451	0.9943	0.61213
	HER2+HR+	0.2860	0.4191	-0.5355	1.1075	0.4951	
	HER2+HR-	Ref	Ref	Ref	Ref		
Endocrine therapy receipt	Yes	0.2844	0.2891	-0.2822	0.8509	0.3252	0.32523
	No	Ref	Ref	Ref	Ref		
Ethnicity	Non-Hispanic	-0.6850	0.1835	-1.0448	-0.3253	0.0002	0.00019
	Hispanic	Ref	Ref	Ref	Ref		
Race	White	0.4547	0.5308	-0.5856	1.4951	0.3916	0.39163
	Black or Asian	Ref	Ref	Ref	Ref		
Timepoint	36 months	-0.3413	0.4422	-1.2080	0.5255	0.4403	0.10658
	12 months	0.0131	0.2248	-0.4274	0.4536	0.9536	
	6 months	-0.6129	0.3049	-1.2106	-0.0153	0.0444	
	Baseline	Ref	Ref	Ref	Ref		
Regional Nodal Radiation receipt	Yes	-0.7791	0.4867	-1.7331	0.1749	0.1094	0.10945
	No	Ref	Ref	Ref	Ref		

© 2023 Johnson HM et al. *JAMA Network Open.*
