# Supplementary material for: Patient-Reported Outcomes of Omission of Breast Surgery Following Neoadjuvant Systemic Therapy: A Nonrandomized Clinical Trial
Source: JAMA Netw Open. 2023 Sep 14;6(9):e2333933. doi: 10.1001/jamanetworkopen.2023.33933 (PMC10502524; doi:10.1001/jamanetworkopen.2023.33933)
Supplement: Supplement 3. — Nonauthor Collaborators [file jamanetwopen-e2333933-s003.pdf]

Supplemental Online Content: Nonauthor Collaborators
*First name, last name, and suffix (if applicable) are required and will appear in PubMed.

*Group Name(s): Exceptional Responders Study Group							
***First Name and Middle Initial(s)**	***Last Name**	***Suffix (eg, Jr, III)**	Academic Degrees	Institution	Location (city, state/province, country)	Role or Contribution, eg, chair, principal investigator	Group (if more than 1 Group listed in the byline) and/or Subgroup (eg, Steering Committee)
Tanya W	Moseley		MD	The University of Texas MD Ande	Houston, Texas, USA	Exceptional responder study group, contributor	
Jessica WT	Leung		MD	The University of Texas MD Ande	Houston, Texas, USA	Exceptional responder study group, contributor	
Monica	Huang		MD	The University of Texas MD Ande	Houston, Texas, USA	Exceptional responder study group, contributor	
Rosalind P	Candelaria		MD	The University of Texas MD Ande	Houston, Texas, USA	Exceptional responder study group, contributor	
Beatriz E	Adrada		MD	The University of Texas MD Ande	Houston, Texas, USA	Exceptional responder study group, contributor	
Elsa	Arribas		MD	The University of Texas MD Ande	Houston, Texas, USA	Exceptional responder study group, contributor	
Raquel FD	van la Parra		MD, PhD	The University of Texas MD Ande	Houston, Texas, USA	Exceptional responder study group	
Kelly K	Hunt		MD	The University of Texas MD Ande	Houston, Texas, USA	Exceptional responder study group, contributor	
Isabelle	Bedrosian		MD	The University of Texas MD Ande	Houston, Texas, USA	Exceptional responder study group, contributor	
Mediget	Teshome		MD, MPH	The University of Texas MD Ande	Houston, Texas, USA	Exceptional responder study group, contributor	

Supplemental Online Content: Nonauthor Collaborators
*First name, last name, and suffix (if applicable) are required and will appear in PubMed.

***First Name and Middle Initial(s)**	***Last Name**	***Suffix (eg, Jr, III)**	Academic Degrees	Institution	Location (city, state/province, country)	Role or Contribution, eg, chair, principal investigator	Group (if more than 1 Group listed in the byline) and/or Subgroup (eg, Steering Committee)
Rosa F	Hwang		MD	The University of Texas MD Ande	Houston, Texas, USA	Exceptional responder study group, contributor	
Makesha V	Miggins		MD	The University of Texas MD Ande	Houston, Texas, USA	Exceptional responder study group, contributor	
Matthew J	Piotrowski		MD	The University of Texas MD Ande	Houston, Texas, USA	Exceptional responder study group, contributor	
Ana P	Refinetti		MD	The University of Texas MD Ande	Houston, Texas, USA	Exceptional responder study group, contributor	
Richard A	Ehlers		MD	The University of Texas MD Ande	Houston, Texas, USA	Exceptional responder study group, contributor	
Jessica	Suarez Colen		MD, MPH	The University of Texas MD Ande	Houston, Texas, USA	Exceptional responder study group, site-leader, contributor	
Catherine E	Loveland-Jones		MD, MS	MD Anderson Cancer Center at C[illegible]	Camden, New Jersey, USA	Exceptional responder study group, site-leader, contributor	
Beth Ann	Lesnikowski		MD	The Breast Institute at JFK Medic[illegible]	Atlantis, Florida, USA	Exceptional responder study group, site-leader, contributor	
Laila	Samiian		MD	Baptist MD Anderson Cancer Cen	Jacksonville, Florida, USA	Exceptional responder study group, site-leader, contributor	

Supplemental Online Content: Nonauthor Collaborators
*First name, last name, and suffix (if applicable) are required and will appear in PubMed.

***First Name and Middle Initial(s)**	***Last Name**	***Suffix (eg, Jr, III)**	Academic Degrees	Institution	Location (city, state/province, country)	Role or Contribution, eg, chair, principal investigator	Group (if more than 1 Group listed in the byline) and/or Subgroup (eg, Steering Committee)
Clayton D	Chong		MD	The Queen's Health System	Honolulu, Hawaii, USA	Exceptional responder study group, site-leader, contributor	
