# Supplementary material for: Patient-Reported Outcomes of Omission of Breast Surgery Following Neoadjuvant Systemic Therapy: A Nonrandomized Clinical Trial
Source: JAMA Netw Open. 2023 Sep 14;6(9):e2333933. doi: 10.1001/jamanetworkopen.2023.33933 (PMC10502524; doi:10.1001/jamanetworkopen.2023.33933)
Supplement: Supplement 4. — Data Sharing Statement [file jamanetwopen-e2333933-s004.pdf]

Data Sharing Statement

Johnson. Patient-Reported Outcomes of Omission of Breast Surgery Following Neoadjuvant Systemic Therapy. *JAMA Netw Open*. Published September 14, 2023. doi:10.1001/jamanetworkopen.2023.33933

Data
Data available: Yes
Data types: Deidentified participant data, Data dictionary
How to access data: Deidentified participant data and a data dictionary will be made available to others on acceptance of an official request to MD Anderson Cancer Center, Houston, TX, USA, after Institutional Review Board approval for release and explicit approval of the study investigators and a signed data usage agreement between the participating institutions as required.
When available: With publication

Supporting Documents
Document types: None

Additional Information
Who can access the data: Deidentified participant data and a data dictionary will be made available to others on acceptance of an official request to MD Anderson Cancer Center, Houston, TX, USA, after Institutional Review Board approval for release and explicit approval of the study investigators and a signed data usage agreement between the participating institutions as required.
Types of analyses: Deidentified participant data and a data dictionary will be made available to others on acceptance of an official request to MD Anderson Cancer Center, Houston, TX, USA, after Institutional Review Board approval for release and explicit approval of the study investigators and a signed data usage agreement between the participating institutions as required.
Mechanisms of data availability: Deidentified participant data and a data dictionary will be made available to others on acceptance of an official request to MD Anderson Cancer Center, Houston, TX, USA, after Institutional Review Board approval for release and explicit approval of the study investigators and a signed data usage agreement between the participating institutions as required.
